# Using deep autoencoders to identify abnormal brain structural patterns in neuropsychiatric disorders: A large‐scale multi‐sample study

**DOI:** 10.1002/hbm.24423

**Published:** 2018-10-11

**Authors:** Walter H. L. Pinaya, Andrea Mechelli, João R. Sato

**Affiliations:** ^1^ Center of Mathematics, Computing, and Cognition Universidade Federal do ABC São Bernardo do Campo SP Brazil; ^2^ Center for Engineering, Modeling and Applied Social Sciences Universidade Federal do ABC São Bernardo do Campo SP Brazil; ^3^ Department of Psychosis Studies Institute of Psychiatry, Psychology & Neuroscience, King's College London London UK

**Keywords:** autism spectrum disorder, computational psychiatry, deep autoencoder, deep learning, schizophrenia, structural MRI

## Abstract

Machine learning is becoming an increasingly popular approach for investigating spatially distributed and subtle neuroanatomical alterations in brain‐based disorders. However, some machine learning models have been criticized for requiring a large number of cases in each experimental group, and for resembling a “black box” that provides little or no insight into the nature of the data. In this article, we propose an alternative conceptual and practical approach for investigating brain‐based disorders which aim to overcome these limitations. We used an artificial neural network known as “deep autoencoder” to create a normative model using structural magnetic resonance imaging data from 1,113 healthy people. We then used this model to estimate total and regional neuroanatomical deviation in individual patients with schizophrenia and autism spectrum disorder using two independent data sets (*n* = 263). We report that the model was able to generate different values of total neuroanatomical deviation for each disease under investigation relative to their control group (*p* < .005). Furthermore, the model revealed distinct patterns of neuroanatomical deviations for the two diseases, consistent with the existing neuroimaging literature. We conclude that the deep autoencoder provides a flexible and promising framework for assessing total and regional neuroanatomical deviations in neuropsychiatric populations.

## 1 INTRODUCTION

Structural magnetic resonance imaging (sMRI) enables the in vivo investigation of the morphological features of the human brain. There is much hope that this tool will help elucidate the neuroanatomical correlates of neuropsychiatric disease, leading to improved detection and treatment (Abou‐Saleh, 2006; Klöppel et al., 2012). However, despite the very large number of scientific publications in this area over the past two decades, the use of sMRI in real‐world clinical decision‐making remains very limited. One of the reasons is that the vast majority of existing studies have used traditional mass‐univariate analytical methods which are sensitive to gross and localized differences in the brain. These techniques are not optimal for detecting neuroanatomical alterations in neuropsychiatric disorders which tend to be subtle and spatially distributed (Durston, 2003; Ellison‐Wright, Glahn, Laird, Thelen, & Bullmore, 2008).

Machine learning—an area of artificial intelligence concerned with the development of algorithms and techniques to learn to perform tasks from examples—provides an alternative analytical approach for estimating neuroanatomical alterations from neuroimaging data (Orrù, Pettersson‐Yeo, Marquand, Sartori, & Mechelli, 2012; Sabuncu, Konukoglu, & Initiative, 2015; Vieira, Pinaya, & Mechelli, 2017). As an inherently multivariate approach, machine learning is sensitive to distributed and subtle differences between experimental groups. However, to develop a machine learning system capable of performing categorization tasks with high reliability, the model must be able to perform accurate mapping of the input data to the desired output in most of the possible space of new samples. Due to the high dimensionality of the data, this usually demands a large number of cases in each experimental group (Nieuwenhuis et al., 2012; Whelan & Garavan, 2014). In practice, this can be challenging, for example when comparing specific clinical sub‐groups who are difficult to recruit in large numbers (e.g., patients with schizophrenia who did and did not respond to a specific treatment). Besides this limitation, some machine learning algorithms (e.g., deep neural networks) have been criticized for resembling a “black box” due to the difficulty of interpreting their inner workings. For example, even when an algorithm allows detection of patients and controls with high levels of accuracy, it can be difficult to establish which specific features of the data informed the categorization decision. Therefore, even in the presence of a successful algorithm, we may gain little or no mechanistic understanding of the disease under investigation. This limits the translational applicability of the findings, since the development of new treatments is normally informed by the underlying mechanisms.

In this article, we adopt an alternative conceptual and practical approach for investigating neuropsychiatric disorders which try to overcome the above limitations. Instead of developing a system for classifying individuals into different groups (e.g., psychiatric patients and healthy subjects), we use neuroimaging data from disease‐free individuals to define the normal range of neuroanatomical variability in the absence of illness. Patients with patterns of brain anatomy which fall outside this normal range would then be identified as outliers (Marquand, Rezek, Buitelaar, & Beckmann, 2016; Mourão‐Miranda et al., 2011; Sato, Rondina, & Mourão‐Miranda, 2012). A further advantage of this approach, which is often referred to as “anomaly detection”, is that it allows the identification of the pathological patterns which underlie the disease under investigation.

To implement this approach, we used the so‐called autoencoder—an artificial neural network which comprises of two components. The first component, that is, the “encoder”, learns to codify the input data in a latent code that is known as latent representation. As part of this step, the data are being compressed resulting in a reduction of dimensionality. The second component, that is, the “decoder”, learns to use the latent representation to reconstruct the input data as close as possible to the original. Therefore, an autoencoder is an artificial neural network designed to output a reconstruction of its input. Due to the constrained size of the latent code, the autoencoder is forced to learn about the underlying structure of the data to create a good reconstruction. To achieve this, during training, the model tries to preserve as much of the relevant information as possible, while intelligently discarding redundancy parts. With the advance of deep learning (LeCun, Bengio, & Hinton, 2015), it is possible to create and train deep autoencoders (i.e., autoencoders with several hidden layers between the input and output layers) capable of learning increasingly complex encoding‐decoding functions. Here the appeal is that the model learns efficient representations of the data such that the original input can be reconstructed in full. In the recent literature, a number of studies have applied deep autoencoders for data denoising (Feng, Zhang, & Glass, 2014; Xie, Xu, & Chen, 2012). These applications estimated the amount of noise by calculating the difference between the reconstructed and inputted data, and then used this estimation to remove the effects of noise from the data.

In this study, we used neuroimaging data from disease‐free individuals to create a deep autoencoder for detecting and elucidating neuroanatomical deviations in individual patients. First, we trained a model with morphometric data from healthy controls from a large‐scale data set: the Human Connectome Project (HCP; Van Essen et al., 2013). The resulting model learns to encode the healthy patterns from the input data and then, from the encoded representation, tries to reconstruct the input data as close as possible to the original. After training this model, we used it to encode and reconstruct the data from two public data sets with psychiatry patients. These data sets composed of patients with schizophrenia (SCZ) and autism spectrum disorder (ASD); in addition, each data set included a healthy control (HC) group composing of disease‐free individuals. The difference between the original input data and the reconstructed output was captured by a “deviation metric” which provided a measure of neuroanatomical alteration in a given individual. For each data set, we compared the mean deviation metric of the patient and the respective healthy control groups. Next, we compared the performance of the normative model against a traditional classifier, using support vector machines. Finally, we analyzed the regional distribution of the reconstruction error and derived the most altered regions for each patient group. We hypothesized that (a) the autoencoder would generate different deviation metrics in patients and controls, with higher mean deviation metrics in the former relative to the latter, and that (b) the autoencoder would reveal different patterns of neuroanatomical deviations for SCZ and ASD, consistent with the existing neuroimaging literature on these disorders.

## 2 METHODS

### 2.1 Data description

The data used in this study were obtained from three public data sets: Human Connectome Project (HCP) data set, Northwestern University Schizophrenia Data and Software Tool (NUSDAST) data set, and Autism Brain Imaging Data Exchange (ABIDE) data set. The NUSDAST data set was obtained using the SchizoConnect (http://schizconnect.org/), a virtual database for public schizophrenia neuroimaging data. The ABIDE data set was acquired from the Neuroimaging Informatics Tools and Resources Clearinghouse (NITRC) image repository (http://www.nitrc.org/). Finally, the HCP data set was acquired from the data management platform called ConnectomeDB (https://db.humanconnectome.org). Detailed information about these data sets and their acquisition parameters is presented in the Supporting Information.

### 2.2 Subjects

In this study, we used sMRI data from 1,113 healthy controls taken from the “1200 Subjects Data Release (S1200 Release, March 2017)” which is part of the HCP data set (see http://www.humanconnectome.org/documentation/S1200/ for technical information). We also analyzed sMRI data from two further clinical data sets including the NUSDAST data set, which composed of healthy controls and patients with SCZ, and the ABIDE data set (http://fcon_1000.projects.nitrc.org/indi/abide/abide_I.html), which composed of HC subjects and ASD patients balanced for age and sex. From these two clinical data sets, we identified and selected those subjects within the same age range of the HCP data set (from 22 to 37 years old). This resulted in 40 healthy controls and 35 patients with SCZ from the NUSDAST data set and 105 healthy controls and 83 subjects with ASD from the ABIDE data set, who were included in the present investigation.

### 2.3 MRI processing

We used the FreeSurfer data from the 1,113 healthy controls taken from the HCP data set (Glasser et al., 2013). These data—including cortical thickness and anatomical structural volume—have already been extracted using the Freesurfer pipeline version 5.3.0 and made available to the scientific community from the HCP. For the NUSDAST and ABIDE data sets, we used the same FreeSurfer pipeline (version 5.3.0) to estimate the cortical thickness and anatomical structural volumes from the T1 weighted images. This estimation was performed using the “recon‐all” command (see Fischl, 2012, Fischl et al., 2002 for more information). The cortical surface of each hemisphere was then parcellated according to the Desikan–Killiany atlas (Desikan et al., 2006) and the anatomical volumetric measures were obtained via a whole brain segmentation procedure (Fischl et al., 2002). This procedure allowed us to calculate the cortical thickness for each of the 68 cortical subregions (34 per hemisphere) and the volume of 36 neuroanatomical structures; therefore, the total number of subregions/structures being investigating was 104.

### 2.4 Deep autoencoder training

**Figure 1:**
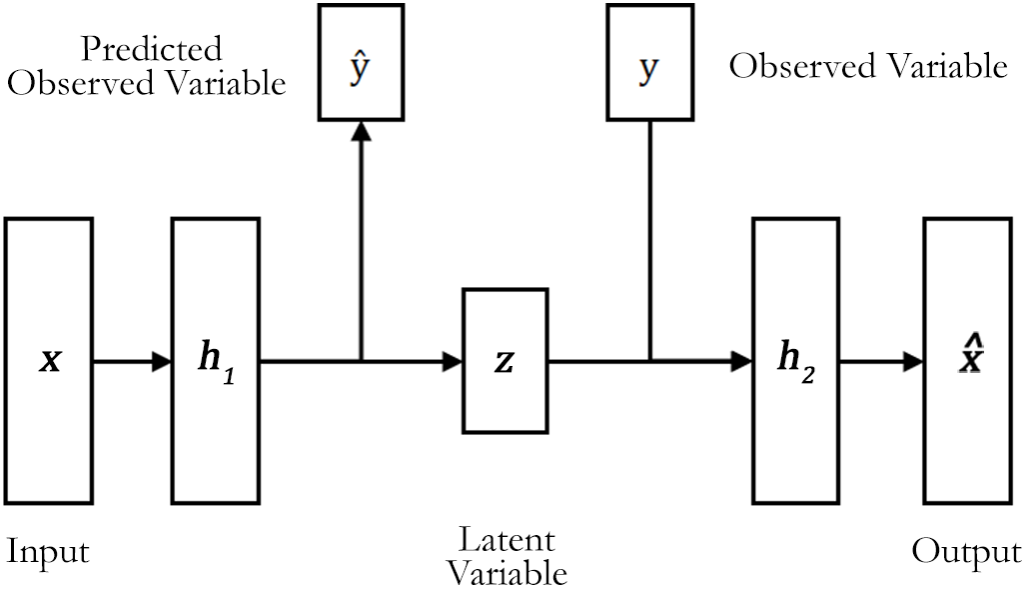
The semi‐supervised deep autoencoder structure. During the training, the deep autoencoder learns to reconstruct the input data and to predict the observed variables *y*, in this case, the subject's age and sex

We created a deep autoencoder that learns to encode and decode brain data using the healthy subjects from the HCP data set (Figure 1). This autoencoder had three hidden layers (h_1_, z, and h_2_). To improve the generalization of the model and avoid overfitting, we applied an L2 regularization (regularization parameter = 1 × 10^−3^) that penalized high values in the network's weights and facilitated diffuse weight vectors as solutions. To mitigate the network's internal covariate shift, the h_1_, z, and h_2_ layers were formed using scaled exponential linear units (SELUs; Klambauer, Unterthiner, Mayr, & Hochreiter, 2017). The activation function of these units allows for faster and more robust training, that is, less training epochs to reach convergence, and a strong regularization scheme (Klambauer et al., 2017). We initialized the SELU units using the appropriated initializer (Klambauer et al., 2017). The output layer was formed by linear units initialized with Glorot initialization, also known as Xavier initialization (Glorot & Bengio, 2010), using weight parameters sampled from a uniform distribution.

The deep autoencoder was trained using all subjects from the HCP data set. In our model, we used a similar approach to a denoising autoencoder (Vincent, Larochelle, Bengio, & Manzagol, 2008) to improve the model robustness. This involved (a) partially corrupting the brain data during training using an additive Gaussian noise (mean = 0 and standard deviation [*SD*] = 0.1); (b) presenting this corrupted data to the autoencoder, and (c) using a loss function to make the model recover the original noise‐free data. This loss function was composed by the mean squared error between the reconstruction of the corrupted input data and the desired output. This metric mainly guided the optimizer (i.e., the neural network's trainer) to adjust the autoencoder parameters during training. This approach enables the model to learn to distill important features from the data while minimizing the influence of noise (Vincent, Larochelle, Lajoie, Bengio, & Manzagol, 2010).

The training process was performed with 2,000 training epochs, that is, the autoencoder processed the whole data set 2,000 times. As an optimizer, we used a gradient‐based method with adaptative learning rates called Adam (Kingma & Ba, 2014). We specified the initial learning rate of the optimizer as 0.05 with an exponential learning rate decay over each epoch (reaching 0.0005 at the end of the training epochs). Finally, the training was configured as mini‐batch gradient descent, using mini‐batches with a size of 64 samples.

In our study, the model was trained by using a semi‐supervised approach. In contrast with the usual approach used in the classification of neuroimaging data, in which the influence of potential confounding variables is removed from the data, we incorporated such confounding variables in our model. This approach allowed our autoencoder to create reconstructions of each subject based on the available information. Similar to Cheung, Livezey, Bansal, and Olshausen (2014), we added information about our samples (in our case, age and sex values) in the structure of the model. Given a subject brain data x and the corresponding age *y*_age_ and sex *y*_sex_, we considered these variables to be elements of the high‐level representation of the brain data input. In particular, we incorporated supervised learning within the model to enable learning of age and sex. Within this semi‐supervised framework, the remaining latent variable *z* must account for the remaining variations of the input data.

The final loss function to train the deep autoencoder is defined as the sum of four separate cost terms (Equation (1)).

(1) $$\text{Loss}={\left(x-\hat{x}\right)}^{2}+\text{Crossentropy}\left(y_{\mathrm{age}},{\hat{y}}_{\mathrm{age}}\right)+\text{Crossentropy}\left(y_{\mathrm{sex}},{\hat{y}}_{\mathrm{sex}}\right)+\text{XCov}\;$$

The first term is the previously mentioned reconstruction cost for an autoencoder measured by the mean squared error formula. The second term is a supervised cost for the prediction of age. In this study, we used a common cost function for deep neural networks—the cross‐entropy between the predictions and the true values. This cost guides the training of the neural network to a solution where the output ${\hat{y}}_{\mathrm{age}}$ (being part of $\hat{y}$ in Figure 1) is as close as possible to the true age *y*_age_. To implement this, we used a classification scheme where each class corresponds to one of the possible ages (i.e., we had 16 classes, indicating ages from 22 to 37). The third term is a standard supervised cost for prediction of sex computed in a similar way to age. These supervised costs ensure that the encoder tries to learn the features related to the confounding variables. Finally, the fourth term XCov is the unsupervised cross‐covariance cost which guides the training to select solutions that disentangle the confounding variables (i.e., age and sex) from the other latent features of the data.

The training data (HCP data set) was normalized; this involved subtracting the mean from every input feature and then dividing the resulting value by the *SD* of the feature (known as zero mean unit variance normalization). This normalization was also applied to the test set (i.e., NUSDAST and ABIDE data sets) using the same parameters, mean and *SD*, from the training set to avoid biased results. We applied these feature scaling to standardize the range value of data and to adjust it to near to zero. This standardization improves the convergence speed of the optimization algorithm during the training of the model (LeCun, Bottou, Orr, & Müller, 2012). Furthermore, it allows the combination of different metrics from the same input modality (e.g., subcortical volume and cortical thickness from structural data), as well as the comparison of deviation metrics derived from different input modalities (e.g., structural vs. functional data). The age and sex variables were transformed to a one‐hot coding for the classification scheme.

### 2.5 Analysis of data sets with psychiatry patients

After training using the HCP data set, we defined the average squared reconstruction error along all brain features as a metric of brain deviation of each subject (Equation (2)).

(2) $$\text{Deviation metric}=\frac{1}{\text{Number of regions}}\sum_{i=1}^{\text{Number of regions}}{\left(x_{i}-{\hat{x}}_{i}\right)}^{2}$$

where *x*_*i*_ is the original value of the brain region *i*, ${\hat{x}}_{i}$ is the deep autoencoder reconstructed value of the brain region *i*, and number of regions is the number of cortical subregions and neuroanatomical structures used (i.e., number of regions = 104).

Then, we used the model to measure the quantity of deviation of the brain data from the NUSDAST and ABIDE data sets based on what was learned from the HCP sample. Since the deviation metric (based on mean squared error) did not follow a normal distribution and presented a number of outliers, we used a nonparametric test, known as two‐tailed Mann–Whitney *U* test, to verify whether the medians of deviation metric are significantly different between healthy controls and patients for each clinical group. To avoid the effects of different sites, scanners, and populations, we restricted statistical comparisons to patient and control groups from the same data set.

### 2.6 Comparison with traditional machine learning classification

Normative methods differ from traditional machine learning classification in several aspects. For example, the data used to train the model are different. In normative models, subjects' categories are not necessary (unsupervised learning), while in traditional classification, it is necessary to specify the classes of each participant (supervised learning). Another difference is what the model learns during training. In traditional classification, the model learns about the values of the features that best discriminate the categories. On the other hand, normative approaches learn the values of features that are considered a typical observation. Even with these distinct characteristics, the normative approach can be adapted to perform classification once assuming patients as outliers (Mourão‐Miranda et al., 2011). Once we set a limit value in the normative deviation metric, we can categorize subjects in HC and patient groups, and, finally, use performance metrics, like accuracy, to compare methods.

In our study, to compare the performance of our normative model against a traditional classification approach, we performed a machine learning analysis of both clinical data sets using Support Vector Machines (SVM; Cortes & Vapnik, 1995). First, we used the data from the ABIDE and NUSDAST data sets as input to the SVM model with the features normalized using the mean and *SD* from the Human Connectome Project. The rationale for using these normalized features was to ensure the consistency of the input data between the autoencoder and the traditional classification model. Also, we used a bootstrap resampling method to estimate the performance of the classifier and quantify its uncertainty using confidence intervals (CI) (DiCiccio & Efron, 1996; Jain, Duin, & Mao, 2000). This involved (a) determining the size of the training set as 70% of the total number of subjects in the data set (resulting in 53 training samples in NUSDAST and 132 training samples in ABIDE); (b) randomly sampling (with replacement) the subjects to create a bootstrap training set; and (c) using all subjects not included in the training set to create a test set.

Having defined the training and test sets, we trained a linear SVM classifier to discriminate between the HC and patient categories. The first step of the training was to define the soft margin (C) hyperparameter, which controls the trade‐off between having zero training errors and allowing misclassifications. In our study, we chose the value of C by performing a grid search using a cross‐validation scheme based on the training set. In brief, using stratified 10‐fold cross‐validation, we divided the training set into 10 parts with the same proportion of HC subjects and patients. We then used nine parts to compose a new training set, and the remaining part was used as the validation set. With these sets defined, we chose one C value from the search space, which was defined as {2^−15^, 2^−13^, 2^−11^, 2^−13^, …, 2^11^, 2^13^, 2^15^} consistent with previous studies (Hsu, Chang, & Lin, 2003). Next, we trained the model on the new training set and computed its balanced accuracy using the validation set. This process was performed 10 times using the same C value across all possible different choices of validation set. Then, we performed this process again with all other possible C values. In the end, we selected the C value that had the higher cross‐validated mean balanced accuracy. With this C value, we trained a linear SVM model again using the whole training data set and, finally, we computed the probabilities of each subject in the test set to belong to the patient group. This approach, including the use of stratified 10‐fold cross‐validation to minimize bias, is consistent with recommended practice (Salvador et al., 2017). The implementation of the SVM classifiers was performed in Python (version 3.6) using the Scikit‐learn library (version 0.19.2; Pedregosa & Varoquaux, 2011).

In the final step, the probabilities of each subject in the test set to belong to the patient group were used to estimate the performance of the classifier. In the present study, we used the area under the receiver operating characteristic curve (AUC‐ROC) as a performance metric for the comparison with the normative approach. With the AUC‐ROC, it is possible to estimate how well the classifier performs without having to explicitly define a threshold value for deciding whether a subject should be classified as HC subject or patient. After obtaining the AUC‐ROC, we repeated the whole process but this time with a new bootstrap training set and test set. This process was repeated 1,000 times to create a distribution of the performance of the classifier. From this distribution, we reported the median performance of the SVM and its CI.

Similar to the classifier evaluation, we computed the AUC‐ROC and its CI for the normative method. In this case, we created bootstrap training sets from the HCP data set, sampling (with replacement) 1,113 subjects to train the normative model. After training, we normalized the clinical data sets using the mean and the *SD* from the original HCP data set (to ensure consistency between autoencoder and the traditional classification). Then, we calculated the deviation metric of all subjects, using these deviation metrics and the actual label of the subjects, we computed the AUC‐ROC. This process was repeated 1,000 times to create a distribution of the performance of the normative approach. From this distribution, we reported the median performance and its CI.

### 2.7 Patterns of neuroanatomical deviations

We investigated the reconstruction error of each brain region in the two clinical samples (SCZ and ASD) using the deep autoencoder. We compared the values of the reconstruction error in patients against HC subjects using the Mann–Whitney *U* test to check for statistically significant regional deviations. A Bonferroni correction for multiple comparisons would have been inappropriate because statistical inferences in homotopic or adjacent regions were most likely to be correlated rather than independent. In the absence of any established procedure, we controlled for false positive rates by using a conservative statistical threshold of *p* < .01 which yield an expected false positive rate of 1%. Finally, we calculated Cliff's delta (Cliff, 1993) absolute value to measure the magnitude of neuroanatomical deviations. Here Cliff's delta value measures how often the deviation metric values in one distribution (i.e., patient group) are larger than the values in a second distribution (i.e., HC group).

### 2.8 Performance evaluation of different network configurations

In this study, the number of neurons per layer was chosen using the training/validation data from the HCP data set. This involved executing a 10‐fold cross‐validation process where the training set was divided into two groups: training and validation set. Thus, we adopted a grid search to select the optimal number of neurons (i.e., among 10, 25, 50, 75, and 100) in each hidden layer. We decided to use a second hidden layer with fewer units than the first layer to constrain the latent variables of the deep autoencoder. We defined the optimum model structure as the one that presented the lowest average reconstruction error at the validation folds during the cross‐validation process. After determining the optimum values, the deep autoencoder was trained again with the best configuration and using both training and validation set. Then, the deep autoencoder analysis was performed on the others data sets (i.e., test sets).

### 2.9 Experiments

We conducted the experiments in Python using the Tensorflow v.1.4 (Abadi et al., 2016) and Keras v.2.1 (https://keras.io/) libraries. We used the same random seed in all our calculations to ensure the starting weights and cross‐validation fold division was equivalent in every set of experiments.

## 3 RESULTS

### 3.1 Performance evaluation for different number of neurons

**Figure 2:**
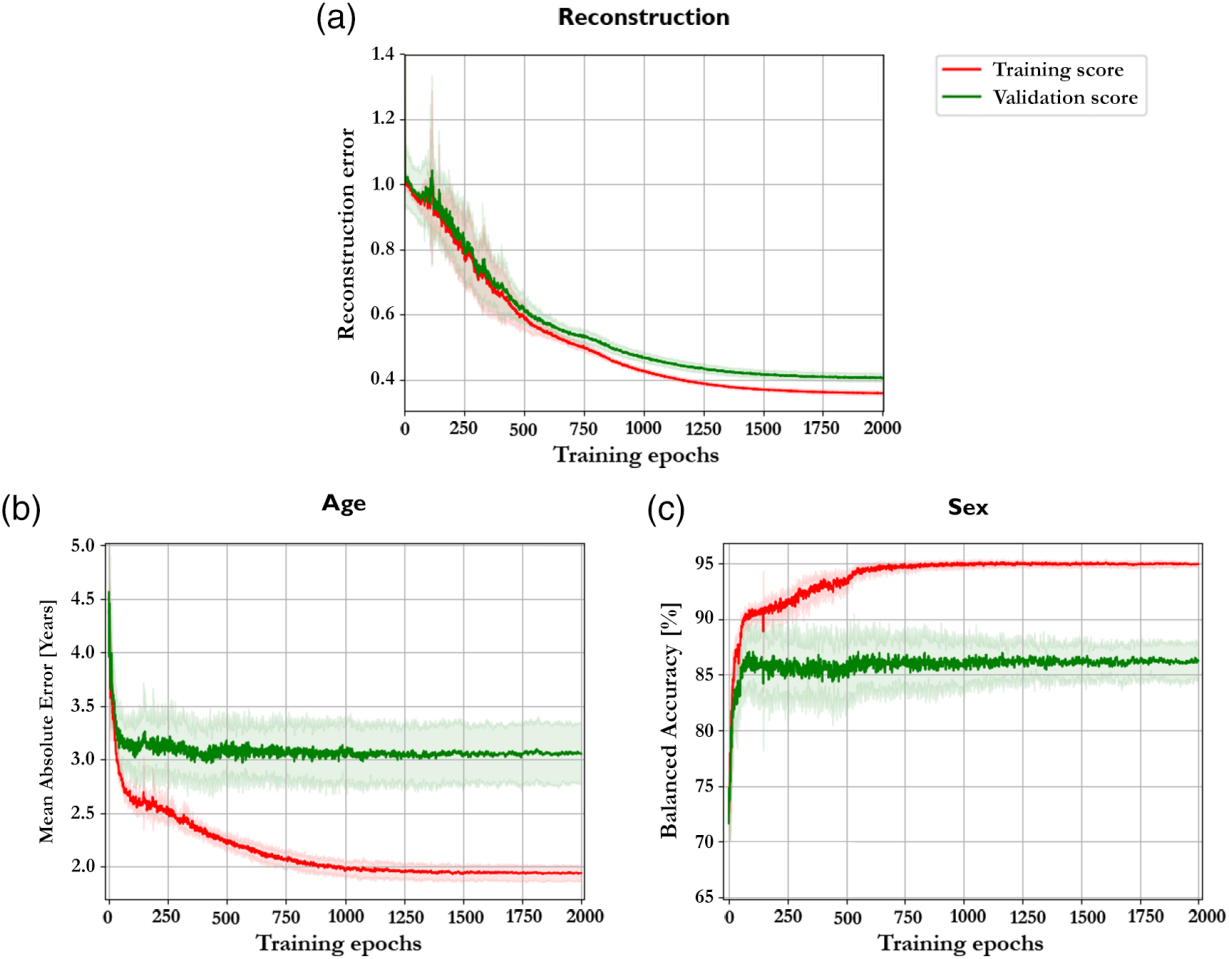
(a) The mean learning curve of the best structure (100–75–100) along the 10‐fold cross‐validation. (b) The mean absolute error curve of age prediction of the best configuration along the 10‐fold cross‐validation. (c) The balanced accuracy curve of sex prediction of the best configuration along the 10‐fold cross‐validation [Color figure can be viewed at http://wileyonlinelibrary.com]

We executed a cross‐validation process on the HCP data to determine the best number of neurons for the layers of our deep autoencoder. We obtained the best performance from the structure with the 104 ➔ 100 ➔ 75 ➔ 100 ➔ 104 configurations (input data ➔ h1 layer ➔ z‐layer ➔ h2 layer ➔ reconstruction) with mean reconstruction error of 0.40 ± 0.01 (the cross‐validation performance of all structures is presented in the Supporting Information). This configuration also presented an age prediction with a mean absolute error of 3.05 ± 0.28 years and a sex prediction with a mean balanced accuracy of 86.25% ± 1.69%. Figure 2 depicts the average learning curve of the best configuration and the evolution of the age and sex predictions performance. The average learning curve of the validation and training sets indicates that 2,000 training epochs and the actual configuration of hyperparameters (including regularization coefficient) appeared to be sufficient for model convergence without falling into overfitting.

### 3.2 Comparison of deviation metrics for patients and healthy controls

In this analysis, we used the deep autoencoder structure with three hidden layers and the 104–100–75–100–104 configurations. We performed the training on the whole HCP data set. After 2,000 training epochs, we obtained a mean reconstruction error of 0.32 on the training set, and we applied the trained model to the others data sets. The deep autoencoder yielded a mean deviation metric of 0.97 ± 0.23 for the HC group and 1.14 ± 0.28 for the SCZ group from the NUSDAST data set (Cliff's delta = 0.4142). The deep autoencoder was also applied to the ABIDE data set, obtaining a mean deviation metric of 1.09 ± 0.30 for the HC group and 1.27 ± 0.40 for the ASD group (Cliff's delta = 0.2764).

**Figure 3:**
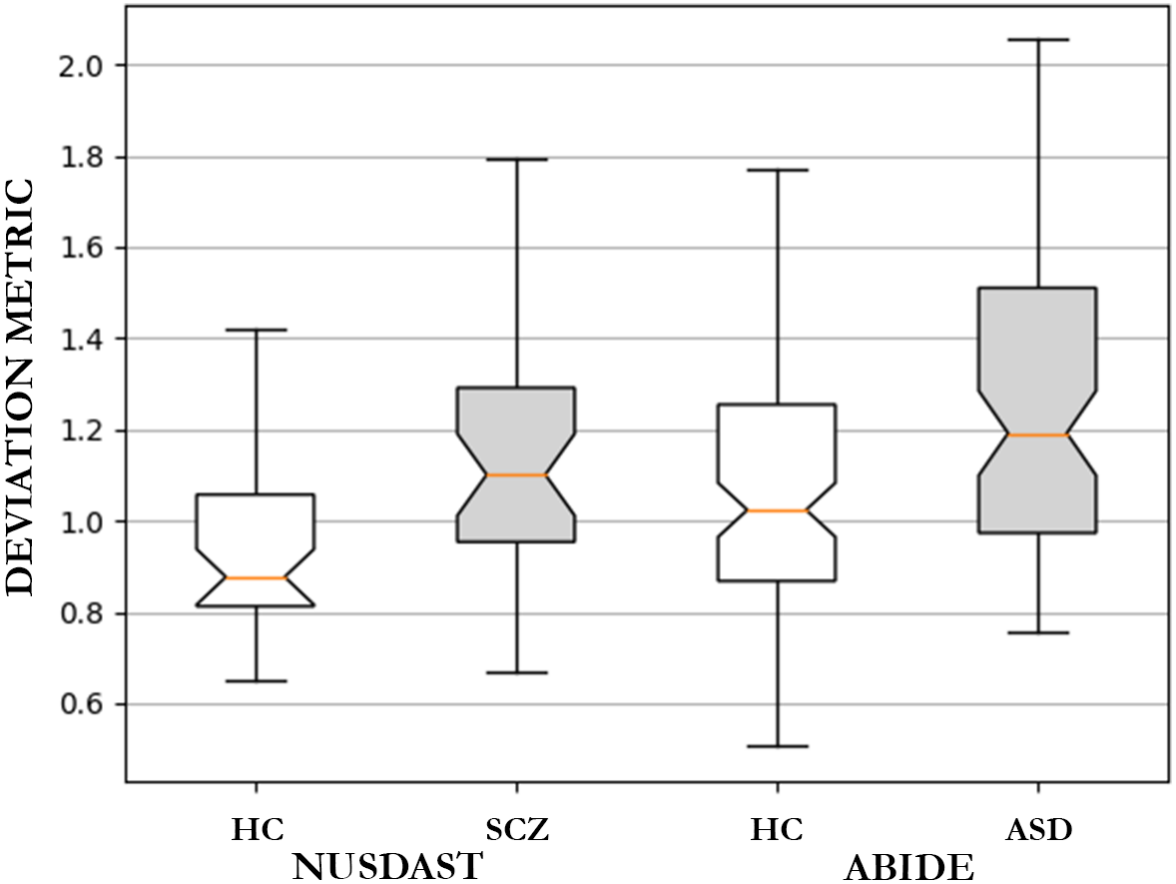
Boxplot of the deviation metric (mean squared reconstruction error) from the patients with schizophrenia group and the healthy controls subjects (NUSDAST data set) and from patients with autism spectrum disorder and the corresponding healthy control group (ABIDE data set). ASD = autism spectrum disorder; HC = healthy controls; SCZ = schizophrenia [Color figure can be viewed at http://wileyonlinelibrary.com]

Figure 3 shows the boxplot indicating the median deviation metric of each group; violin plots are also presented in the Supporting Information. As expected, in the NUSDAST data set, the deviation metric was significantly higher for the SCZ groups than the corresponding HC groups with the Mann–Whitney *U* test presenting a statistically significant difference (*p* = .001). Likewise, the ASD group presented a higher mean deviation metric than the corresponding HC group with the Mann–Whitney *U* test presenting a statistically significant difference (*p* < .001).

### 3.3 Prediction of age and sex for patients and healthy controls

In addition to the estimation of deviation metrics, the trained model predicts the age and sex of each individual using a semi‐supervised framework (see “Deep autoencoder training” section for detail). For the NUSDAST data set, the model predicted age with a mean absolute error (MAE) of 3.40 years in the HC group and 3.57 years in the SCZ group. For the same data set, the model also predicted sex with accuracies of 75.00% in the HC group and 62.28% in the patient group. For the ABIDE data set, the model predicted the age with an MAE of 4.02 years in the HC group and 3.83 years in the ASD group. Here the model also predicted sex with accuracies of 79.04% in the HC group and 78.31% in the patient group, respectively.

### 3.4 Comparison with traditional classifiers

In the NUSDAST data set, the linear SVM obtained a median AUC‐ROC = 0.637 (95% CI = [0.486, 0.766]), whereas using the deviation metric of the normative approach, we obtained an AUC‐ROC = 0.707 (95% CI = [0.662, 0.751]). In the ABIDE data set, the SVM obtained a median AUC‐ROC = 0.569 (95% CI = [0.462, 0.659]), while the normative approach resulted in an AUC‐ROC = 0.639 (95% CI = [0.611, 0.666]). Based on these results, therefore, the performance of our normative model appeared to be comparable to that of traditional classifiers. Other metrics of performance of the classifiers are presented in the Supporting Information.

### 3.5 Reconstruction error in individual regions

To derive the most altered regions for each patient group, we investigated the reconstruction error of each brain region (violin plots and the comparison between original vs. reconstructed values for each brain region and data set are presented in the Supporting Information). Using the Mann–Whitney *U* test, we verified which region had different median values of reconstruction error between healthy subjects and patients. We then measured Cliff's delta absolute value to quantify the effect size of the pathological changes on the reconstruction error for each region. For each data set, the brain structures showing a statistically significant difference are shown in Table 2. The full list of regions with *p* values and effect sizes is presented in the Supporting Information.

## 4 DISCUSSION

In this study, we used a deep autoencoder to map brain data from healthy subjects to a latent representation and then map this back to reconstruct the brain data used as input. The resulting model was then applied to two independent data sets, each including healthy subjects as well as neuropsychiatric patients. In each data set, the model performed better (i.e., it yielded a smaller reconstruction error corresponding to a smaller deviation metric) when applied to brain data from healthy controls than when applied to brain data from patients. Consistent with our first hypothesis, therefore, the model was effective in generating different deviation metrics in healthy controls and patients. Furthermore, we were able to evaluate the contribution of each brain region to the overall reconstruction error of each subject. This procedure revealed statistically significant alterations in several regions that were previously reported in the neuropsychiatric neuroimaging literature. Consistent with our second hypothesis, the autoencoder revealed different patterns of neuroanatomical deviations for SCZ and ASD when compared to healthy controls from the respective data sets.

During the training phase, which used data corrupted by a Gaussian noise, the deep autoencoder learned the most robust representations of healthy people in its multilevel representations (Vincent et al., 2008). From the existing neuroimaging literature, we know that neuropsychiatric populations show alterations in cortical thickness and regional volume relative to healthy people (Ecker et al., 2013; Qiu et al., 2011; Shepherd, Laurens, Matheson, Carr, & Green, 2012). However, since individuals with neuropsychiatric disease were not present in the training set, the deep autoencoder did not learn to map these neuropathological alterations. As expected this resulted in a larger difference between the reconstructed output and the original input when the model was applied to patients relative to when it was applied to healthy people. In other words, each patient group presented a higher mean reconstruction error, indicating higher levels of neuroanatomical deviations, than the HC group from the same data set.

In the present study, we also compared our normative approach with traditional machine learning classification. This revealed that the performance of the two approaches was comparable, with the normative median performance falling within the classifier's confidence interval in both clinical data sets. However, even with similar performances, both methods did not achieve high performance. Using the bootstrap resampling method, our normative approach showed modest AUC‐ROC values between 0.611 and 0.751, while the values shown by the classifier were not significantly different from the random guessing. This pattern of results differs from previous machine learning studies, which have typically reported higher classification accuracies between HC subjects and patients with SCZ and ASD (Kim, Calhoun, Shim, & Lee, 2016; Rozycki et al., 2017; Uddin et al., 2011). However, we note that most of these previous studies used different types of features, such as voxel‐based values or regional functional MRI activations. There were, however, a few studies that performed classification using regional volume and thickness. In Salvador et al. (2017), for example, the author's classified 128 patients with SCZ and 127 HC subjects using a number of structural features, including cortical volume and thickness; similar to our study, the use of SVM classifiers resulted in modest performance, with accuracies around 60%. In Pinaya et al. (2016), using 143 patients with SCZ and 83 HC subjects, the SVM classifier achieved a balanced accuracy of 68.1%. Using 22 children with ASD and 16 HC subjects, Jiao et al. (2010) were able to achieve an AUC‐ROC of 0.93, however, the very low number of subjects may have inflated the estimate of performance (Schnack & Kahn, 2016). In light of these previous studies, therefore, we speculate that the use of regional features may explain the low discriminant performance in our investigation. Due to the dimensionality reduction that occurs during the preprocessing, a significant amount of structural information about the subject's brain may be lost. Such information could be useful for the discrimination between the categories, as suggested by the results of previous studies that used different types of features. In the present study, we chose regional features as input as their low dimensionality would allow us to perform more tests with our limited computational resources. Future studies could expand our investigations by evaluating how the normative approach behaves with different data modalities, such as voxel‐based values or regional functional activation. Finally, is worth to mention that the performed comparison is not a standard approach used in classifiers comparison. Due to the different natures of both methods, it was not possible to test the models in the same conditions (e.g., the same subjects in the training set).

By analyzing the brain data reconstructions, we were also able to consider how much each region differed from its normative value for each patient group. In contrast with from previous studies using normative approaches (Mourão‐Miranda et al., 2011; Sato et al., 2012), the deep autoencoder is capable of generating an individualized brain map that indicates the contribution of each region to the deviation metric of each subject. This information can provide insight into the pathological mechanism which underlies an illness, although it does not completely solve the issue of the interpretability of the model. Below we discuss the main neuroanatomical findings for each diagnostic group in turn.

In patients with SCZ relative to healthy controls, the lateral ventricles were among the regions with the highest difference in the deviation metric (Cliff's delta: left = 0.410; right = 0.328). Increased lateral ventricular size is one of the most consistently reported neuroanatomical abnormalities in schizophrenia (Rimol et al., 2010; Shenton, Dickey, Frumin, & McCarley, 2001; Shepherd et al., 2012). Interestingly, the ventricles were not significantly different between groups in the mass‐univariate analysis using the original volumes (left: Mann–Whitney *U* test; *p* = .349; Cliff's delta = 0.052; right: Mann–Whitney *U* test; *p* = .365; Cliff's delta = 0.047). The apparent inconsistency can be explained by the multivariate nature of our machine learning model. While standard mass‐univariate techniques consider each brain structure as an independent unit, multivariate methods may be additionally based on inter‐regional correlations. An individual region may therefore display high discriminative power due to two possible reasons: (a) a difference in volume/thickness between groups in that region; (b) a difference in the correlation between that region and other areas between groups. Thus, discriminative brain networks are best interpreted as a spatially distributed pattern rather than as individual regions.

Another region showing a statistically significant difference between SCZ and healthy controls was the right superior temporal cortex. This region is also a common finding in neuroimaging studies of schizophrenia, which typically report volume reduction (Shepherd et al., 2012). Alteration of the right superior temporal cortex has been associated with severity of positive symptoms in schizophrenia (Walton et al., 2017). Based on recent studies (Honea, Crow, Passingham, & Mackay, 2005; Shepherd et al., 2012), this alteration usually occurs in both hemispheres, however in the present investigation the left superior temporal cortex did not express a statistical significant group difference in deviation (Mann–Whitney *U* test; *p* = .118; Cliff's delta = 0.160), and did not show a statistically significant effect in the mass‐univariate analysis (Mann–Whitney *U* test; *p* = .027; Cliff's delta = 0.259).

Statistically significant differences in deviations between the SCZ and HC groups were also found in the left precentral cortex. Previous studies suggested that reductions in this regions are part of the neurobiological mechanisms underlying the onset of the illness (Rimol et al., 2010; Shepherd et al., 2012; Zhou et al., 2005). Finally, the left ventral diencephalon was the brain structure with the most different deviation between HC and SCZ groups (Cliff's delta = 0.417). In contrast, this structure was not among the significant structures detected in our mass‐univariate analysis (Mann–Whitney *U* test; *p* = .135; Cliff's delta = 0.148). The ventral diencephalon in Freesurfer includes several structures: hypothalamus with mammillary body, subthalamic, lateral geniculate, medial geniculate and red nuclei, substantia nigra, and surrounding white matter. Even though some of these regions have been reported in studies of patients with schizophrenia (Klomp, Koolschijn, Hulshoff Pol, Kahn, & Van Haren, 2012), they are not a common finding in meta‐analyses and reviews.

There were a few regions that were found to be significantly different in the mass‐univariate analysis but not with respect to the deviation metric; these included, among others, the third ventricle (Mann–Whitney *U* test in deviation metric analysis; *p* = .033; Cliff's delta = 0.247) and the left insular cortex (Mann–Whitney *U* test in deviation metric analysis; *p* = .076; Cliff's delta = 0.192). These regions have often been reported in meta‐analyses and systematic reviews of the neural basis of the disorder (Shepherd et al., 2012).

With respect to patients with ASD relative to healthy controls, the choroid plexus, cuneus, putamen, and cerebellum cortex were found to have significantly different deviations between groups. Differences on the right occipital lobe (specifically the right cuneus), the left putamen, and the cerebellum cortex are also consistent with previous studies (Cauda et al., 2011; Nickl‐Jockschat et al., 2012; Stanfield et al., 2008). These regions were not significant in the mass‐univariate analysis, however, their reconstruction values were affected by the multivariate nature of the model. Studies have indicated that visual perception in ASD patients differs from that of healthy controls and that this can be explained in terms of neuroanatomical differences in occipital areas (Nickl‐Jockschat et al., 2012). In addition alterations of the basal ganglia have been found to correlate with impaired motor performance or repetitive and stereotyped behavior in ASD patients (Nickl‐Jockschat et al., 2012). Surprisingly, the left choroid plexus was the structure with the highest different deviation between groups; however, this structure was not significantly different between groups in the univariate analysis. Once again, this inconsistency could be explained by the fact that multivariate methods can detect significant effects due to two possible reasons: (a) a difference in volume/thickness between groups in that region; (b) a difference in the correlation between that region and other areas between groups.

Taken collectively, these findings suggest that our approach was sensitive to the underlying neuropathological features of the two diseases under investigation. It should be noted, however, that the *SD* of the estimated deviation metrics tended to be high, suggesting high individual variability within each group. This observation may restrict the possible use of this metric to discriminate patients with a neuropsychiatric disease from healthy people at the individual level. This aspect of our findings could be explained by the clinical heterogeneity of our neuropsychiatric samples which is likely to be associated with neuroanatomical heterogeneity. Such clinical and neuroanatomical heterogeneity represents a challenge not only for the approach presented in the present manuscript but also for the field of machine learning applied to neuroimaging as a whole (Klöppel et al., 2012). Finally, we compared each clinical group against their HC group without modeling differences in acquisition protocols and populations; this means that our results do not allow a direct comparison between the two clinical groups under investigation. However, this was not the purpose of the present study, which aimed at creating a deep autoencoder that could be used to compare patients and healthy controls.

The use of a deep neural network framework enabled us to use flexible configurations and model the age and sex variables in a comprehensive and straightforward way. However, we note that this is not a standard approach for the neuroimaging research which tends to adopt strategies for dealing with potential confounding variables such as age and sex. The first strategy involves balancing the groups to be compared with respect to potential confounding variables, whereas the second strategy involves “regressing out” the variability in the data is associated with these variables to minimize their potential influence (Falahati et al., 2016; Linn, Gaonkar, Doshi, Davatzikos, & Shinohara, 2016). Further analysis is needed to investigate the use of semi‐supervised training to deal with potential confounding influences. In this study, we made sure that each comparison was carried out between groups balanced for age and sex (refer to Table 1 for detail) to minimize the impact of this issue.

**Table 1:** Demographic information for the subjects from the Human Connectome Project, Northwestern University schizophrenia data and software tool and Autism Brain Imaging Data Exchange data sets

	HCP (*n* = 1,113)	NUSDAST		*p*	ABIDE		*p*
		HC (*n* = 40)	SCZ (*n* = 35)		HC (*n* = 105)	ASD (*n* = 83)	
Age, y				.180			.607
Mean ± *SD*	28.8 ± 3.7	26.7 ± 4.13	25.5 ± 3.92		27.0 ± 3.9	27.3 ± 4.1	
Range	22–37	22–37	22–36		22–37	22–36	
Sex, *n* (%)				.398			.922
Men	493 (44%)	25 (62%)	26 (74%)		92 (88%)	74 (89%)	
Women	606 (56%)	15 (48%)	9 (26%)		13 (12%)	9 (11%)	

We used Student's *t* test and the chi‐square test to test for significant differences in age and sex between healthy controls and patients.

Abbreviations: ABIDE = Autism Brain Imaging Data Exchange; ASD = autism spectrum disorder; HC = healthy control; HCP = Human Connectome Project data set; NUSDAST = Northwestern University schizophrenia data and software tool; SCZ = schizophrenia.

**Table 2:** Regions that presented a statistically significant difference in reconstruction error between groups for each data set (*p* ≤ .01, Mann–Whitney *U* test)

NUSDAST	Effect size	ABIDE	Effect size
Left ventral diencephalon	0.4171	Left choroid plexus	0.2496
Left lateral ventricle	0.4100	Right cuneus	0.2448
Right superior temporal	0.3871	Left putamen	0.2280
Right lateral ventricle	0.3285	Left cerebellum cortex	0.2216
Left precentral	0.3185	–	–

Abbreviations: ABIDE = Autism Brain Imaging Data Exchange; NUSDAST = Northwestern University schizophrenia data and software tool.

Although the deep autoencoder was successful in identifying different neuropathological patterns for SCZ and ASD, it should not be assumed that our model is capable of detecting all abnormalities in all brain‐based disorders. For example, a neuroanatomical reduction might be a marker of neuropathology in patients with a specific disease, while also being present in some disease‐free individuals as a result of normal neuroanatomical heterogeneity; such reduction would be difficult to detect using our outlier detection model. Another limitation of our investigation is that subtle differences in head motion may have influenced the estimation of the deviation metrics. In neuroimaging, patients may present higher head motion than healthy controls during scanning (Van Dijk, Sabuncu, & Buckner, 2012; Reuter et al., 2015; Savalia et al., 2017); this may interact with the segmentation of the images increasing the risk of artifactual positive or negative findings (see Mechelli, Price, Friston, & Ashburner [2005] for review). In our investigation, therefore, differences in head motion undetectable by visual inspection might be responsible for the higher *SD* of the deviation metric in patients relative to healthy controls. On the other hand, it is also possible that this difference in *SD* reflected a higher degree of neuroanatomical variation in patients relative to controls, consistent with the heterogeneous clinical presentation of the two diseases under investigation.

Another possible source of artifacts in our investigation relates to the preprocessing of the images. Usually, automatic preprocessing systems can provide spurious results (e.g., bad gray and white matter segmentation). This problem is even more frequent in samples with significant ventricular enlargement (Bhalla & Mahmood, 2015; McCarthy et al., 2015), such as SCZ. However, further actions to try to minimize this effect could also introduce subjective bias from the quality evaluator. In our investigation, we therefore chose to not correct preprocessing step by visual assessment to guarantee a fully automatized and reproducible approach. Finally, due to the nonlinear nature of the model, our method does not allow one to establish the direction of the alterations (i.e., increase vs. decrease in volume/thickness) when comparing two groups that were not included in the training process. This means that, in our study, we were unable the direction of the alterations in patients with SCZ and ASD since none of the data used for testing were used for training the autoencoder. One could infer the direction of the deviation by comparing a sample from the test sets (NUSDAST and ABIDE) against the training set (HCP). This however would introduce possible confounds related to effects of different sites, scanners, and populations. To avoid such confounds, we decided to sacrifice the ability to specify the direction of the alterations and compare groups that were part of the same data set.

## 5 CONCLUSIONS

In conclusion, the use of a deep autoencoder enabled us to detect different patterns of neuroanatomical alteration between neuropsychiatric patients and healthy controls on the basis of their reconstruction error. The model was also able to detect distinct patterns of neuroanatomical deviations in SCZ and ASD, indicating consistent performance across different psychiatric disorders. These results suggest that the deep autoencoder can be used to measure the overall deviation metric of an individual and elucidate which regions are the most different compared to healthy group (i.e., a normative range). The deep autoencoder provides a flexible and promising framework which could be applied to different neuroimaging modalities (e.g., functional MRI) and different types of preprocessing (e.g., voxel‐based morphometry) in future studies.

## Supporting information

Additional supporting information may be found online in the Supporting Information section at the end of the article.

Supporting Information
