# Supplementary material for: Using deep autoencoders to identify abnormal brain structural patterns in neuropsychiatric disorders: A large‐scale multi‐sample study
Source: Hum Brain Mapp. 2018 Oct 11;40(3):944–54. doi: 10.1002/hbm.24423 (PMC6492107; doi:10.1002/hbm.24423)
Supplement: Supplementary file 1 — Supporting Information [file HBM-40-944-s001.docx]

**Using deep autoencoders to identify abnormal brain structural patterns in neuropsychiatric disorders: a large-scale multi-sample study**

Walter H. L. Pinaya * ^a b c^, Andrea Mechelli ^c^, João R. Sato ^a^

* ^a^ Center of Mathematics, Computation, and Cognition. Universidade Federal do ABC, Santo André, Brazil.

* ^b^ Center for Engineering, Modeling and Applied Social Sciences. Universidade Federal do ABC, Santo André, Brazil.

^c^ Department of Psychosis Studies, Institute of Psychiatry, Psychology & Neuroscience, King’s College London, London, UK.

* ^a^ Rua Arcturus, 03 - Jardim Antares, São Bernardo do Campo - SP, CEP 09.606-070, Brazil.

* ^b^ Rua Arcturus, 03 - Jardim Antares, São Bernardo do Campo - SP, CEP 09.606-070, Brazil.

^c^ Institute of Psychiatry, Psychology and Neuroscience, King’s College London, De Crespigny Park, London SE5 8AF, UK.

**Corresponding Author**: Walter H. L. Pinaya

**Phone**: +55 11 97123 0508

**Email address:** [walhugolp@gmail.com](mailto:walhugolp@gmail.com)

**Supplementary material**

Contents

[1. Information about the datasets 3](#_Toc524606033)

[2. MRI Acquisition 5](#_Toc524606034)

[3. Performance of different deep autoencoder configurations 6](#_Toc524606035)

[4. Comparison between deep autoencoder and linear method 7](#_Toc524606036)

[5. Contribution of each input feature to the generation of the reconstructed data 11](#_Toc524606037)

[6. Violin plot of the deviation metric 15](#_Toc524606038)

[7. Violin plot of reconstruction error of each region for the NUSDAST dataset 16](#_Toc524606039)

[8. Violin plot of reconstruction error of each region for the ABIDE dataset 20](#_Toc524606040)

[9. Statistical significance and effect sizes of each region from the NUSDAST dataset 24](#_Toc524606041)

[10. Statistical significance and effect sizes of each region from the ABIDE dataset 26](#_Toc524606042)

[11. Mean original and reconstructed values of each region 28](#_Toc524606043)

[12. Mass-univariate analysis of the NUSDAST dataset 63](#_Toc524606044)

[13. Mass-univariate analysis of the ABIDE dataset 65](#_Toc524606045)

[14. Performance of the SVM classifiers 67](#_Toc524606046)

[References 68](#_Toc524606047)

# Information about the datasets

In this study, we used three datasets: the Human Connectome Project, the Northwestern University Schizophrenia Data and Software Tool (which is part of the Schizoconnect database), and the Autism Brain Imaging Data Exchange. Information about the three datastes is povided below.

*Human Connectome Project*

The Human Connectome Project consortium led by Washington University, University of Minnesota, and Oxford University (the WU-Minn HCP consortium) is undertaking a systematic effort to characterize human brain connectivity and function in a large population of healthy adults [Van Essen et al., 2013]. The HCP aims to enable detailed comparisons between brain circuits, behavior, and genetics at the level of individual subjects. In this study, we use part of data from HCP database (<https://db.humanconnectome.org>).

*Northwestern University Schizophrenia Data and Software Tool*

The NUSDAST database is a repository of schizophrenia neuroimaging data that shares sMRI data, genotyping data, and neurocognitive data as well as analysis tools to the schizophrenic research community. In this study, we use part of data from NUSDAST database (http://central.xnat.org/REST/projects/NUDataSharing). As such, the investigators within NUSDAST contributed to the design and implementation of NUSDAST and/or provided data but did not participate in analysis or writing of this report.

*SchizoConnect Project*

The SchizConnect project [Wang et al., 2016] is an initiative that allows combining of neuroimaging data from different databases via mediation to form compatible mega-datasets with high levels of accuracy and fidelity. The NUSDAST data used in this article was obtained from the SchizConnect database (http://schizconnect.org). As such, the investigators within SchizConnect contributed to the design and implementation of SchizConnect and/or provided data but did not participate in analysis or writing of this report.

*Autism Brain Imaging Data Exchange*

The Autism Brain Imaging Data Exchange (tinyurl.com/fcon1000-abide) started as an effort involving 17 international sites dedicated to aggregating and sharing previously collected data. This dataset is composed of resting state functional magnetic resonance imaging, anatomical and phenotypic datasets from individuals with autism spectrum disorder and age-matched typical controls [Di Martino et al., 2014].

# MRI Acquisition

*Human Connectome Project*

The HCP T1-weighted images were collected on a customized Siemens Skyra 3T scanner using a 32-channel head coil. Two separate averages of the T1w image are acquired using the 3D MPRAGE sequence with 0.7mm isotropic resolution (FOV=224 mm, matrix=320, 256 sagittal slices in a single slab), TR=2400 ms, TE=2.14 ms, flip angle =8°.

*Northwestern University Schizophrenia Data and Software Tool*

The NUSDAST T1-weighted images were collected on a Siemens MAGNETOM VISION IMA. The neuroimages are acquired using 3D MPRAGE sequence TR=9.7 ms, TE=4 ms, flip=10°, ACQ=1, 256x256 matrix, 1 mm in-plane resolution, 128 slices, slice thickness 1.25 mm.

*Autism Brain Imaging Data Exchange*

The ABIDE T1-weighted images were collected on multiple sites (twenty different sites in total). The acquisition parameters of each site are available at http://fcon_1000.projects.nitrc.org/indi/abide/abide_I.html.

# Performance of different deep autoencoder configurations

Table 1 – Average reconstruction error of each tested configurations for the deep autoencoder. These values were obtained from the 10-fold cross-validation process performed using the data of the HCP dataset. The configuration chosen for further analyses is highlighted in bold. Details of the procedure followed to evaluate different neural network configuration is presented in Section “2.7 Performance evaluation of different network configurations” of the main document.

| **Configuration** | **Reconstruction error Mean ± S.D.** |
| --- | --- |
| 25-10-25 | 0.610 ± 0.043 |
| 50-10-50 | 0.592 ± 0.029 |
| 75-10-75 | 0.580 ± 0.034 |
| 100-10-100 | 0.591 ±0.043 |
| 50-25-50 | 0.503 ±0.013 |
| 75-25-75 | 0.502 ± 0.017 |
| 100-25-100 | 0.540 ±0.101 |
| 75-50-75 | 0.441 ±0.011 |
| 100-50-100 | 0.482 ± 0.144 |
| **100-75-100** | **0.405 ±0.013** |

# Comparison between deep autoencoder and linear method

In this study, we also performed the computation of the deviation metric using a well-known linear method, the Principal Components Analysis (PCA). Similar to the autoencoder, PCA is capable of performing (i) an encoding process where the input data are represented in the principal components space; and (ii) a decoding process where the representation is transformed back to the input space. Usually, PCA is used to perform dimensionality reduction tasks. This involves transforming the data into the principal components space (using the eigenvectors of the data covariance/correlation matrix). Following this transformation, only a part of the principal components dimensions – the ones that are responsible for explaining most of the variance in the input data (identified by the absolute value of the eigenvalues) - are used to represent the data.

In order to compare the performance of PCA and that of the deep autoencoder, we followed the following procedure. First, we trained the PCA model to map the normalized input data to the principal components space using the whole HCP data. Next, we applied the learned transformation on each subject data from the clinical datasets. Then, we used a representation of the subjects’ data with reduced dimensionality (here we selected only the n principal components that are responsible for the most variance of the input data). Next, we transformed this reduced dimension representation back to the input space. Finally, we calculated the deviation metric using a similar approach to that used for the deep autoencoder (i.e. using the mean squared error between the input data and the reconstruction).

We defined the number of principal components used in the PCA based on the configuration of the deep autoencoder model with the smallest reconstruction error during the cross-validation. This model had 75 artificial neurons in its most abstract layer (the encoded representation); for this reason, when representing the input data into the principal components space, we used 75 dimensions to perform our comparison.

The PCA method obtained a reconstruction error of 0.0355 for the HCP dataset. Figure 1 shows the explained variance of each principal component of the PCA method. Using 75 principal components, the data representation contains (or “explains”) 96.64% of the variance of the input data. The PCA obtained a deviation metric of 0.1183±0.0440 for the SCZ sample and a value of 0.1275±0.0369 for the HC sample of the NUSDAST dataset (p-value = 0.1453; Mann–Whitney U test). In the ABIDE balanced sample, the metric was 0.1241±0.0442 for the patients with ASD and 0.1430±0.0550 for the HC group (p-value = 0.0112; Mann–Whitney U test). For each dataset, we also computed the effect size of the difference between the patient and control groups using Cliff’s delta absolute value. The PCA method achieved an effect size of 0.1428 for the NUSDAST dataset (deep autoencoder’s effect size = 0.4142) and an effect size of 0.1940 for the ABIDE dataset (deep autoencoder’s effect size = 0.2764).


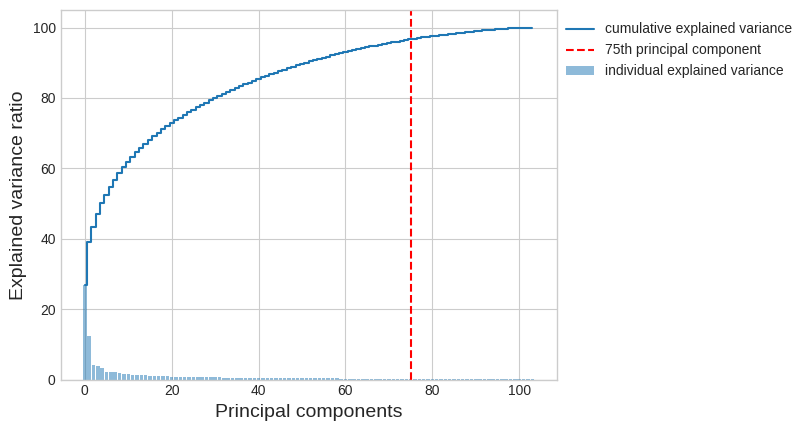


Figure 1 – Cumulative explained variance of the PCA model on the Human Connectome Project dataset. The vertical bars represent the individual explained variance of each principal component.

Therefore, in the analysis of disease-free subjects from the HCP dataset, the PCA approach achieved a smaller reconstruction error than the deep autoencoder. However, even with a better reconstruction error in disease-free subjects, the performance of the PCA approach was worse when it came to differentiating between the patient and control groups within each clinical dataset. In particular, while the PCA approach showed significantly different deviation metric values between the patient and control groups in the ABIDE dataset, it did not do so in the NUSDAST groups. Furthermore, for each clinical dataset, the PCA approach achieved a smaller effect size, indicating lower differentiation between the patient and control groups on the basis of their deviation metrics, than the deep autoencoder. These results might be explained by the capacity of the non-linear function that is incorporated in the deep autoencoder but not the PCA approach; alternatively, they might be explained by the inclusion of potential confounding variables (i.e., age and sex) in the deep autoencoder but not the PCA approach. One limitation of this comparison is that the results are likely to be influenced by the choice of the number of principal components in the PCA model. In particular, the choice of a suboptimal hyperparameter can lead to a loss of information or the introduction of random noise. While there are a plethora of approaches for calculating the optimal number of principal components [Dray, 2008; Valle et al., 1999], none of them is universally recognised as the gold standard. The use of principal components equal to the number of neurons in the most abstract layer of the deep autoencoder might not be the most appropriate, however there is no standard approach to perform a comparison between these two methods.

# Contribution of each input feature to the generation of the reconstructed data

One of the drawbacks of deep artificial neural networks is the difficulty of interpreting its internal computations. This lack of interpretability is the main reason why deep artificial networks are usually referred as “black box” models. In order to address this limitation, several studies have developed different methods Here we used one of these existing methods, known as guided backpropagation with SmoothGrad technique [Smilkov et al., 2017]. This method uses the network gradients to assign an “importance” value to individual elements of the autoencoder (in our case, the input features); this importance value is meant to reflect the influence of each element on the final loss function. Importance values are used to generate the so-called “saliency maps”. In particular, the SmoothGrad technique adds noise in the inputted data and generates the saliency map several times; this leads to the computation of an average saliency map which is thought to provide the most robust measure (for more technical details, see Smilkov et al., 2017).

We used the above method to investigate the importance of each input feature (including the age and sex variables) to the generation of the reconstructed data. This allowed us to estimate the contribution of each brain region to the group classification decision, as well as the importance of each brain region for predicting the age and sex of the subjects. The interpretation of this method involved four main steps. First, we trained the deep autoencoder model using the whole normalized HCP dataset. Second, we used the trained model to generate the reconstructed values of each sample from the HCP dataset. Third, using the guided backpropagation with SmoothGrad, we computed the network gradients based on the inputted data to generate the saliency map. Here the SmoothGrad technique was applied to 50 corrupted saliency maps in order to generate the final saliency map in each subject. Finally, we averaged the saliency map of all subjects within the same group to derive group-level saliency maps. A similar process was performed to determine the importance of the brain regions for predicting the age and sex. In this case the gradients were calculated based on their respective outputs loss function. Figure 2 and 3 show the mean saliency map of the reconstructed brain regions and the predicted age and sex.


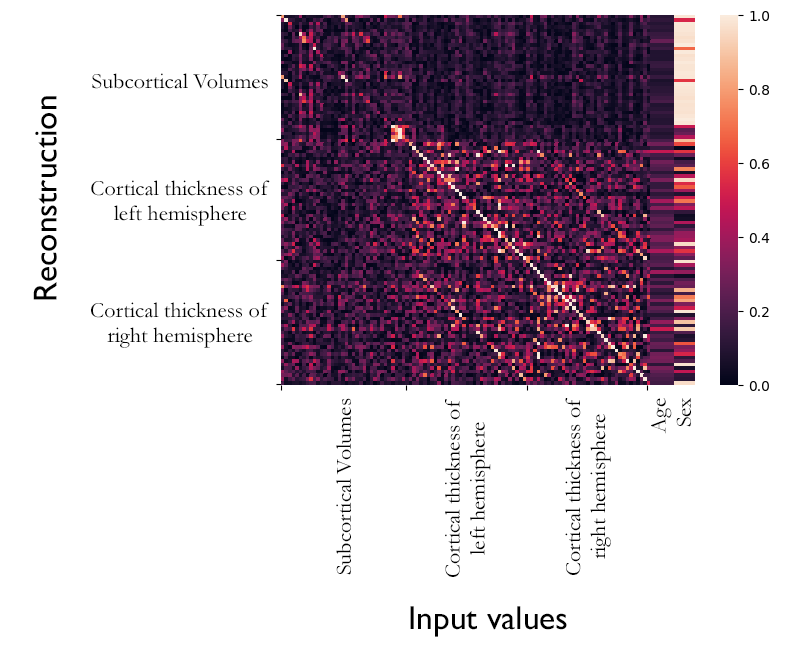


Figure 2 – Mean saliency map of the reconstructed brain regions. The map was normalized to have values between 0 and 1. Each row of the saliency map represents an output unit of the deep autoencoder (i.e., a reconstructed brain region). The columns represent each input feature. The last two columns correspond to the influence of sex and age on the reconstruction of the brain regions.


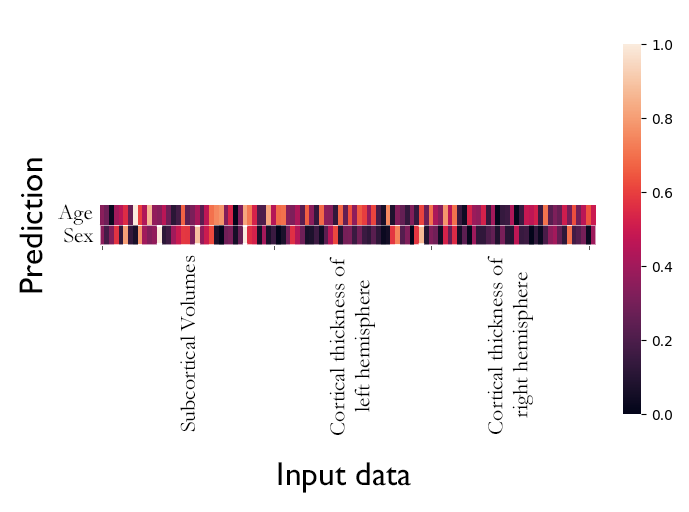


Figure 3 - Mean saliency map of the predicted age and sex. The map was normalized to have values between 0 and 1. The columns represent each input feature.

Based on the mean salience maps, we were able to conclude that the model did not perform a trivial reconstruction of input feature or, in other words, it did not just copy the inputted data through the layers to generate the reconstructed data. This conclusion was based on the observation that the mean saliency map did not show an identity matrix pattern, where main diagonal has value 1 and all others elements have zero value. Instead, as shown in Figures 2 and 3, there was were diffused patterns in relation to the inputted data, especially in the case of cortical thickness. Figure 2 also shows that sex had relative high influence on the reconstruction of the volume of subcortical structures. The reason for the influence of sex on volumetric measures might be that the structural volumes of the subjects were not normalized by the total intracranial volume.

The most influent regions for predicting sex were: optic chiasm volume, left amygdala volume, left cerebellum cortex volume, left temporal pole thickness, left caudate volume, 3^rd^ ventricle volume, left superior frontal thickness, right superior temporal thickness, right putamen volume, left middle temporal thickness, right lateral ventricle volume, right inferior lateral ventricle volume, left frontal pole thickness, left rostral middle frontal thickness, and left cerebellum cortex volume. In contrast, the most influent input features for predicting age were: left pallidum volume, brain stem volume, anterior corpus callosum volume, optic chiasm volume, right hippocampus volume, right caudal middle frontal thickness, left rostral anterior cingulate thickness, right pallidum volume, posterior corpus callosum, right precuneus thickness, left insula thickness, left caudal middle frontal thickness right putamen volume, right entorhinal thickness, and left caudal anterior cingulate thickness.

# Violin plot of the deviation metric

**
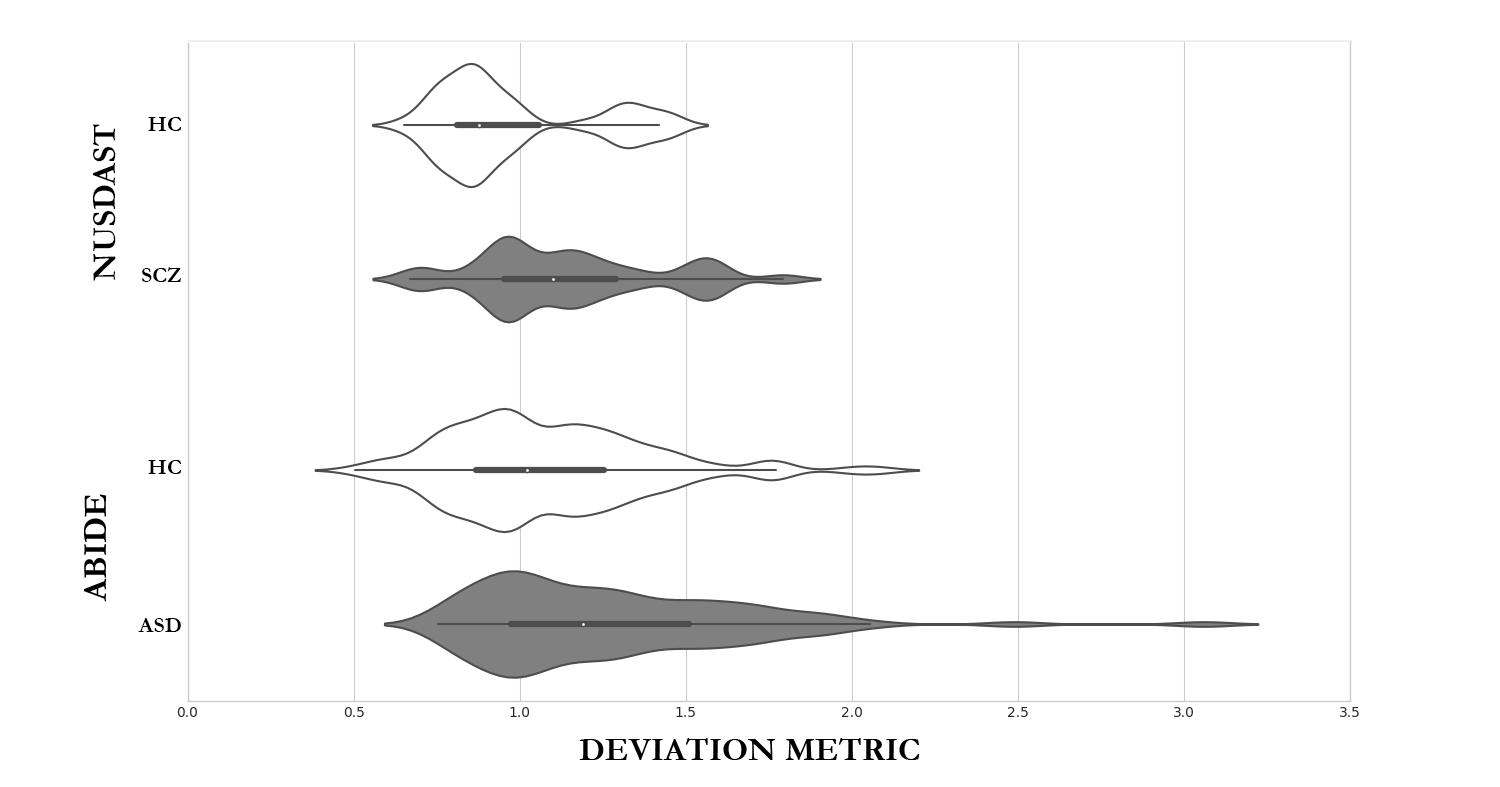
**

Figure 4 - Violin plot of the deviation metric of each group of each dataset. The median and the Interquartile range are presented.

# Violin plot of reconstruction error of each region for the NUSDAST dataset

**
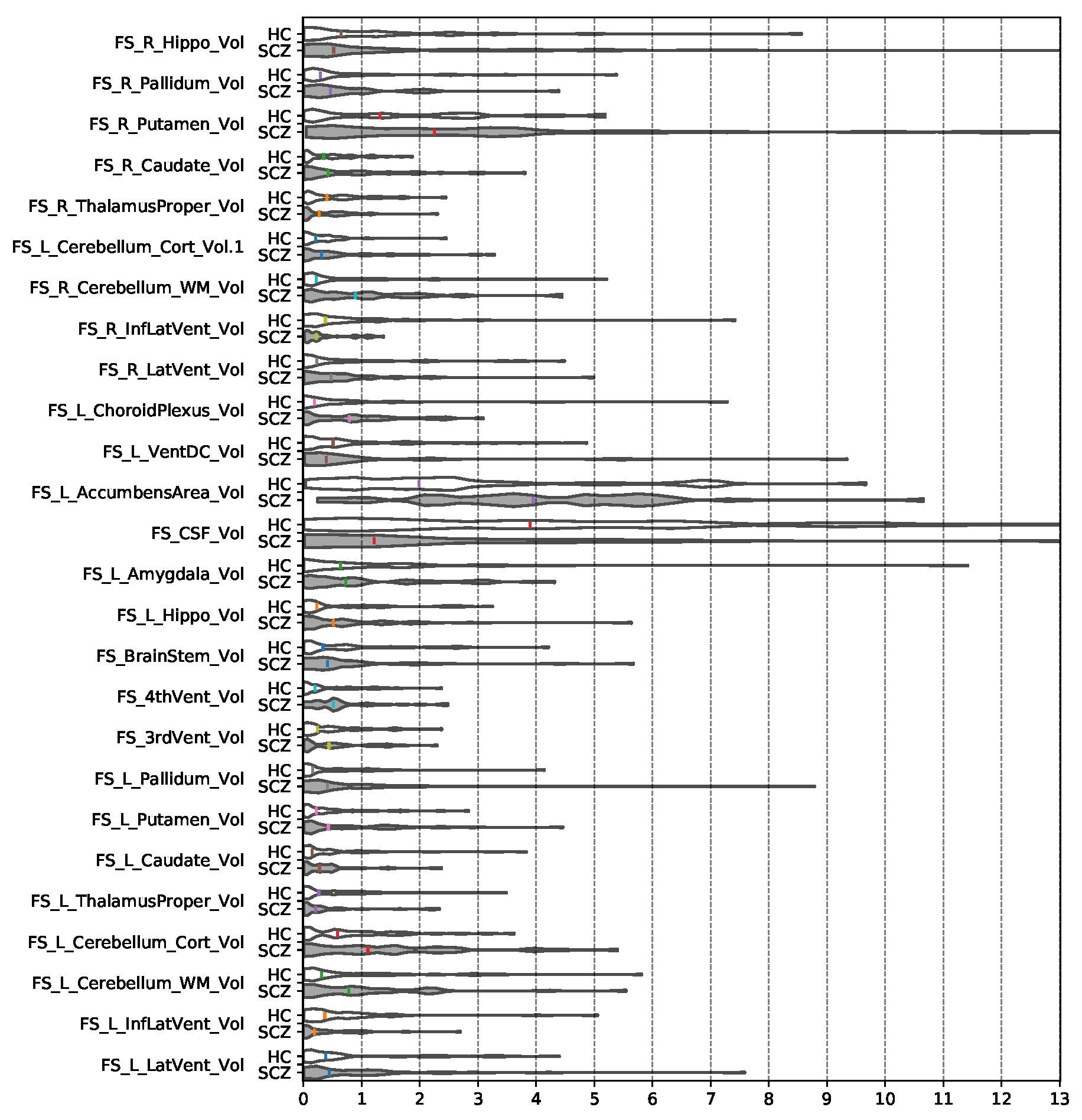
**

**Continuation of violin plot of reconstruction error of each region for the NUSDAST dataset**

**
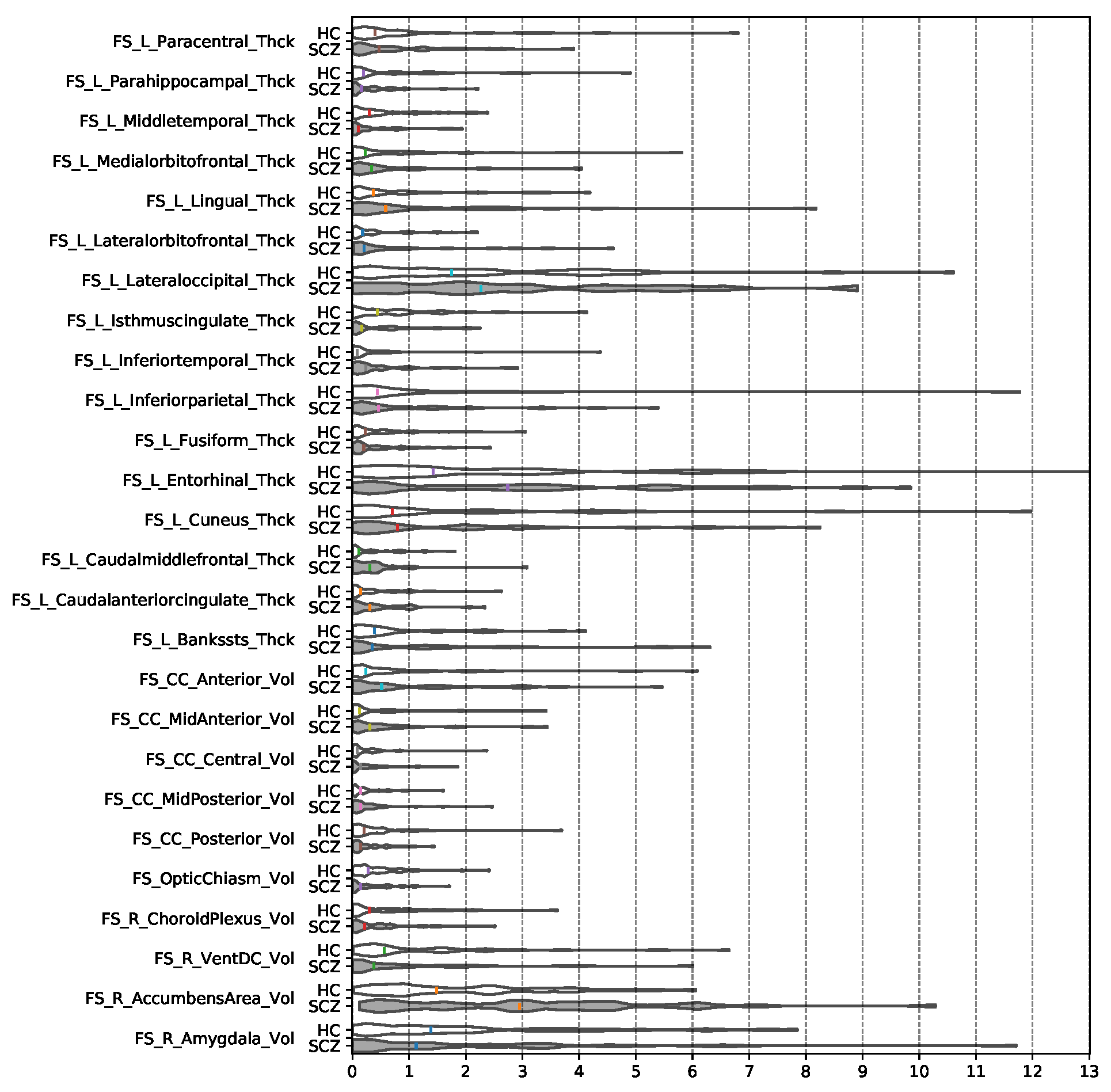
**

**Continuation of violin plot of reconstruction error of each region for the NUSDAST dataset**

**
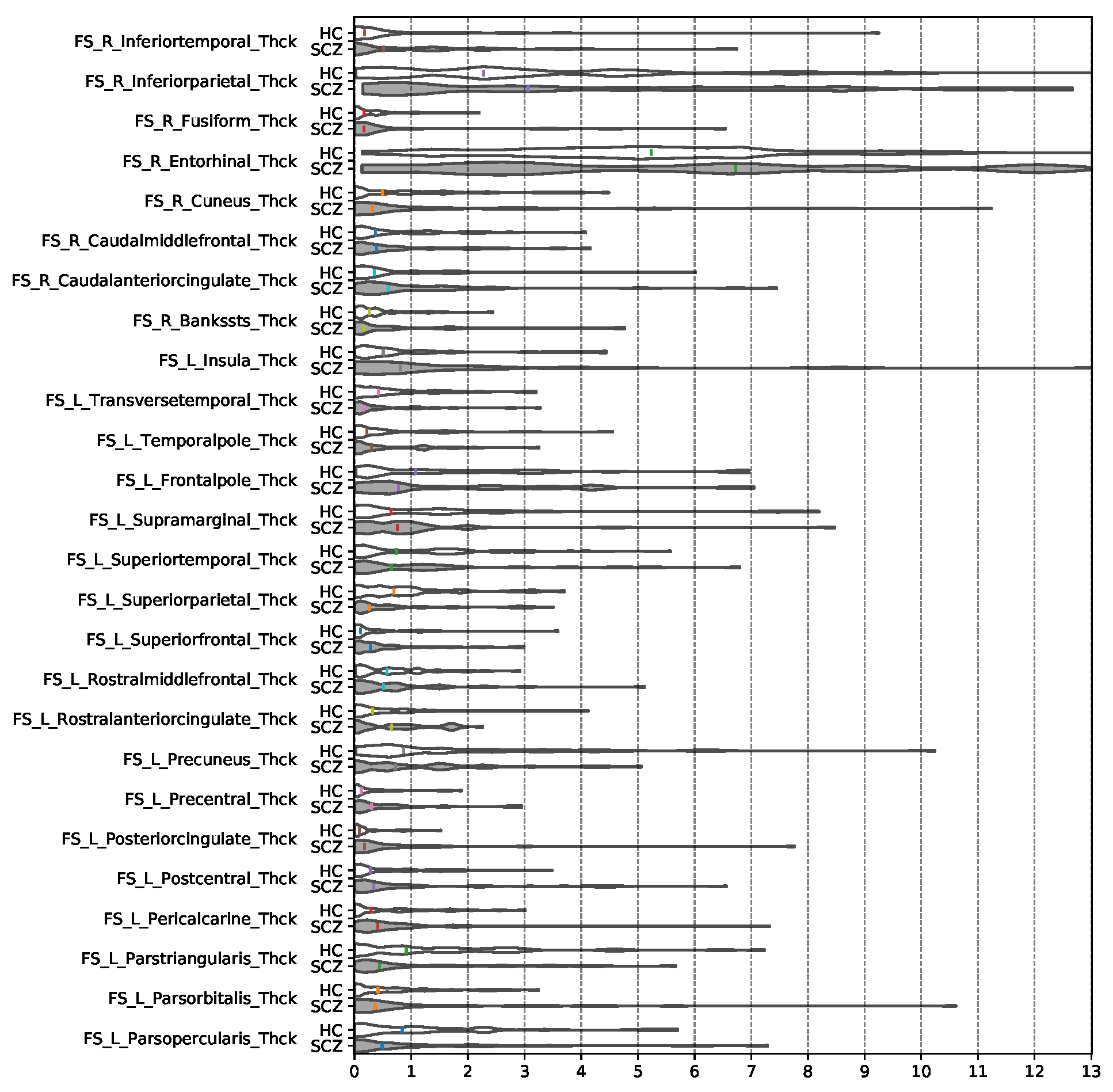
**

**Continuation of violin plot of reconstruction error of each region for the NUSDAST dataset**

**
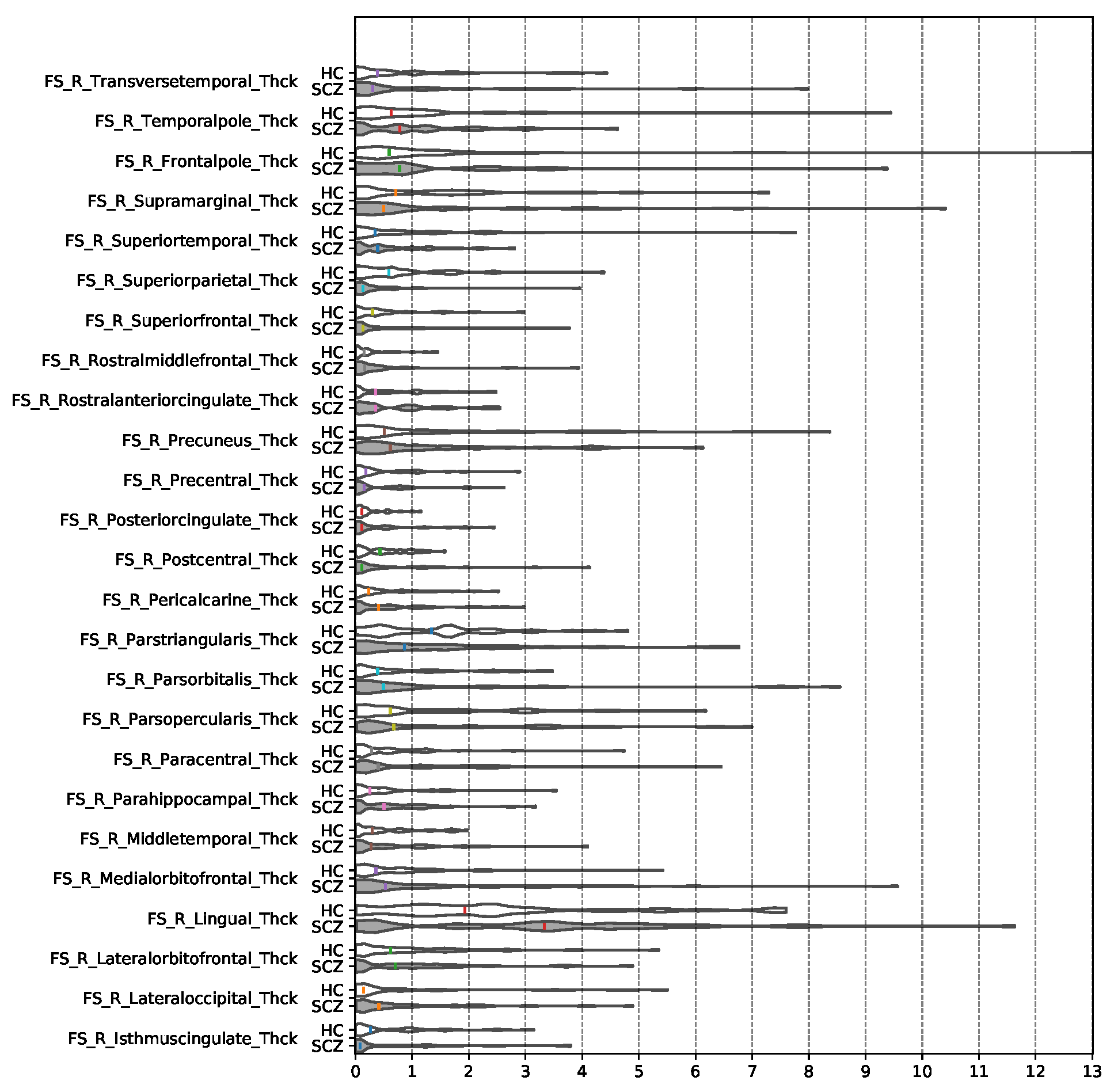
**

Figure 5 - Violin plot of the reconstruction error of each brain regions analyzed by the deep autoencoder using the NUSDAST dataset. The medians of the distributions are indicated by the red line.

# Violin plot of reconstruction error of each region for the ABIDE dataset

**
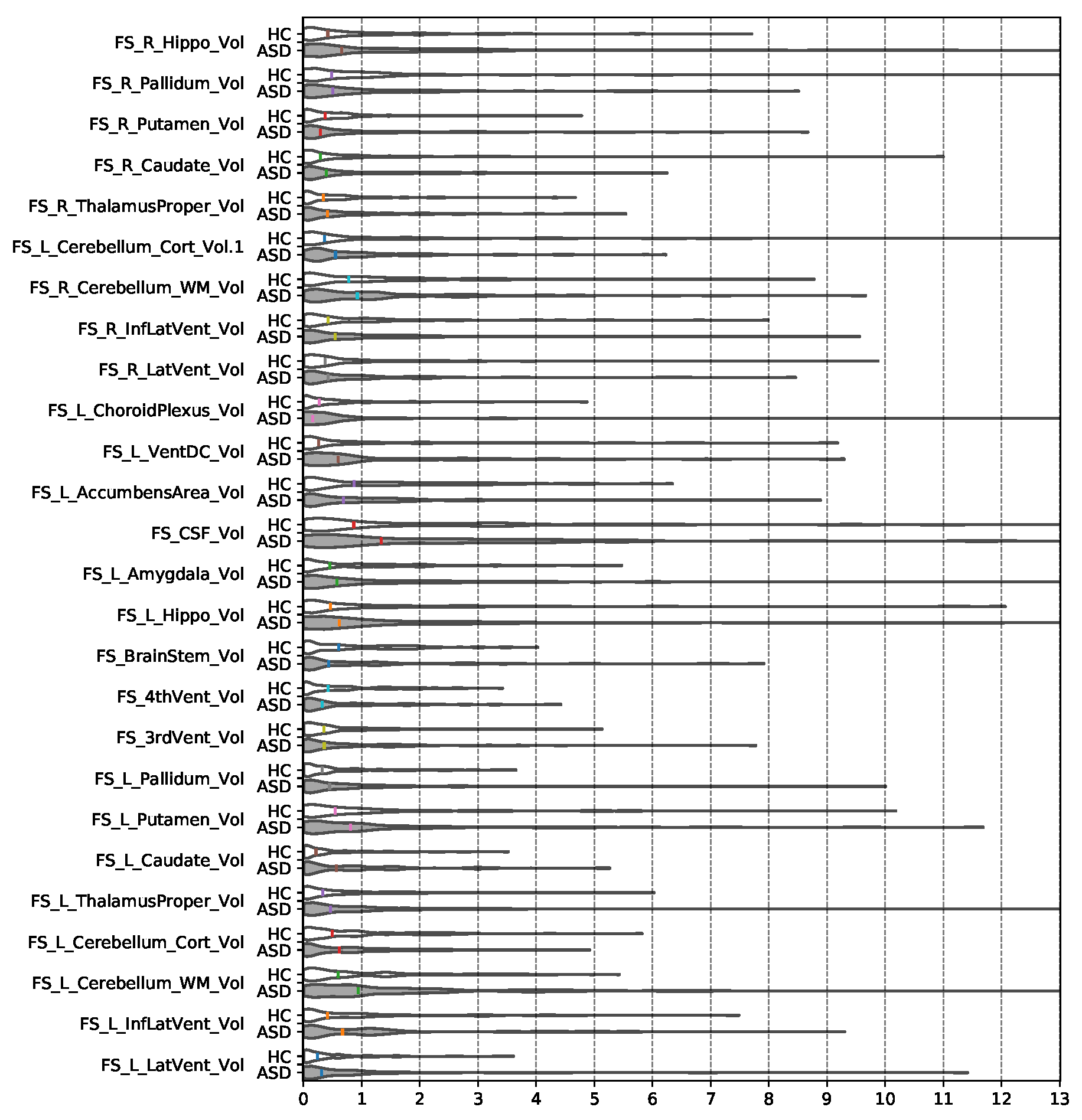
**

**Continuation of violin plot of reconstruction error of each region for the ABIDE dataset**

**
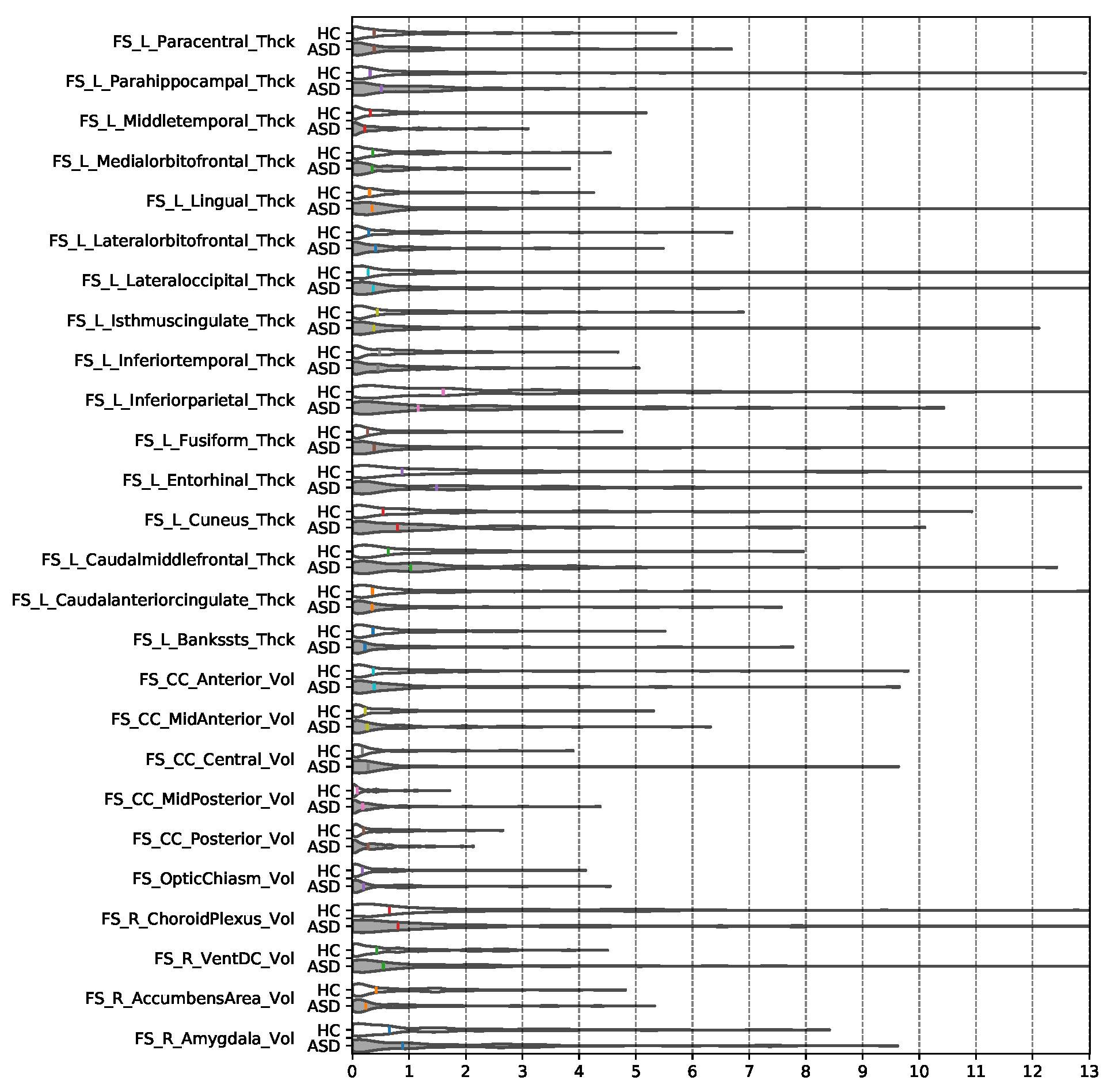
**

**Continuation of violin plot of reconstruction error of each region for the ABIDE dataset**

**
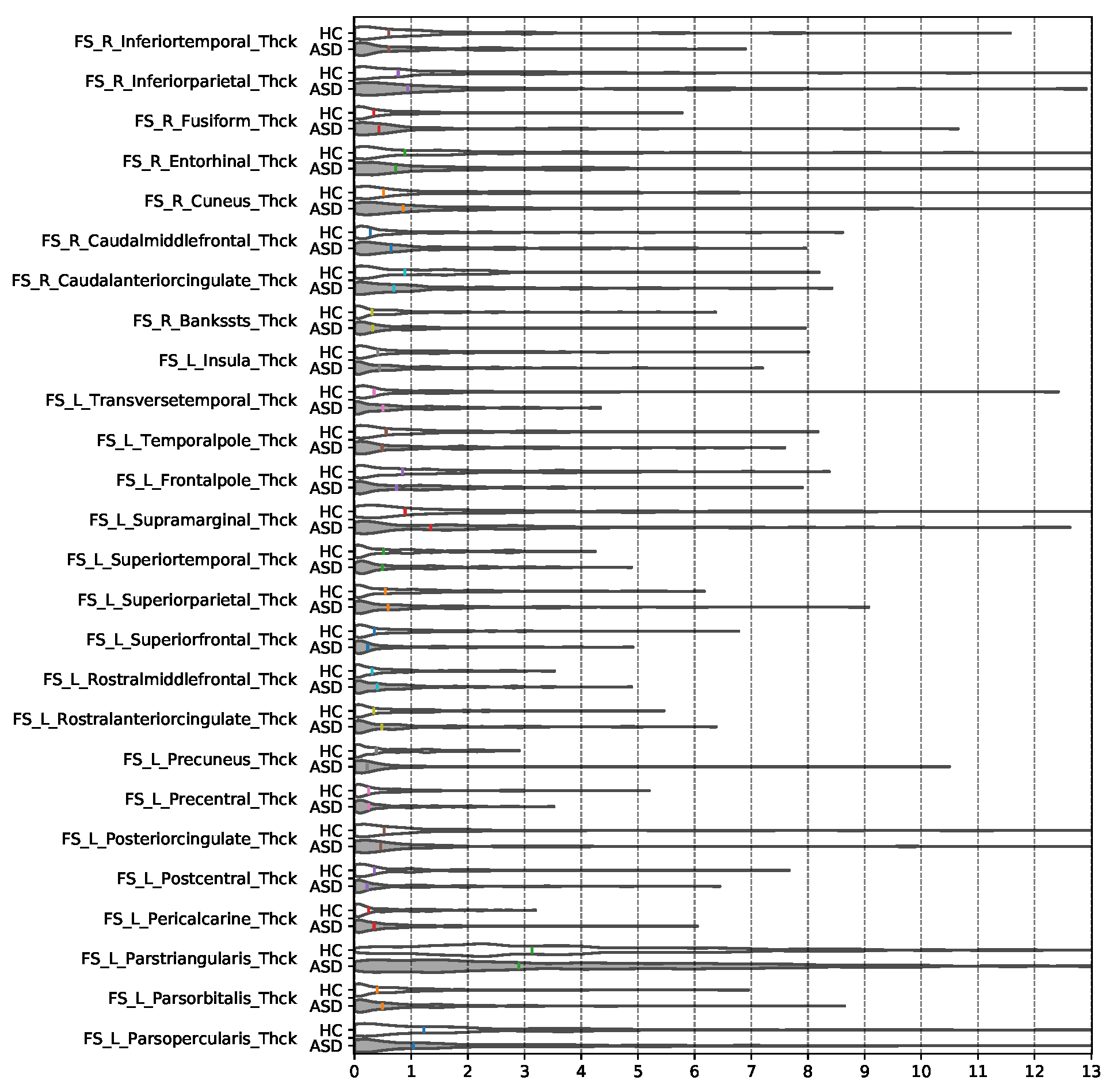
**

**Continuation of violin plot of reconstruction error of each region for the ABIDE dataset**

**
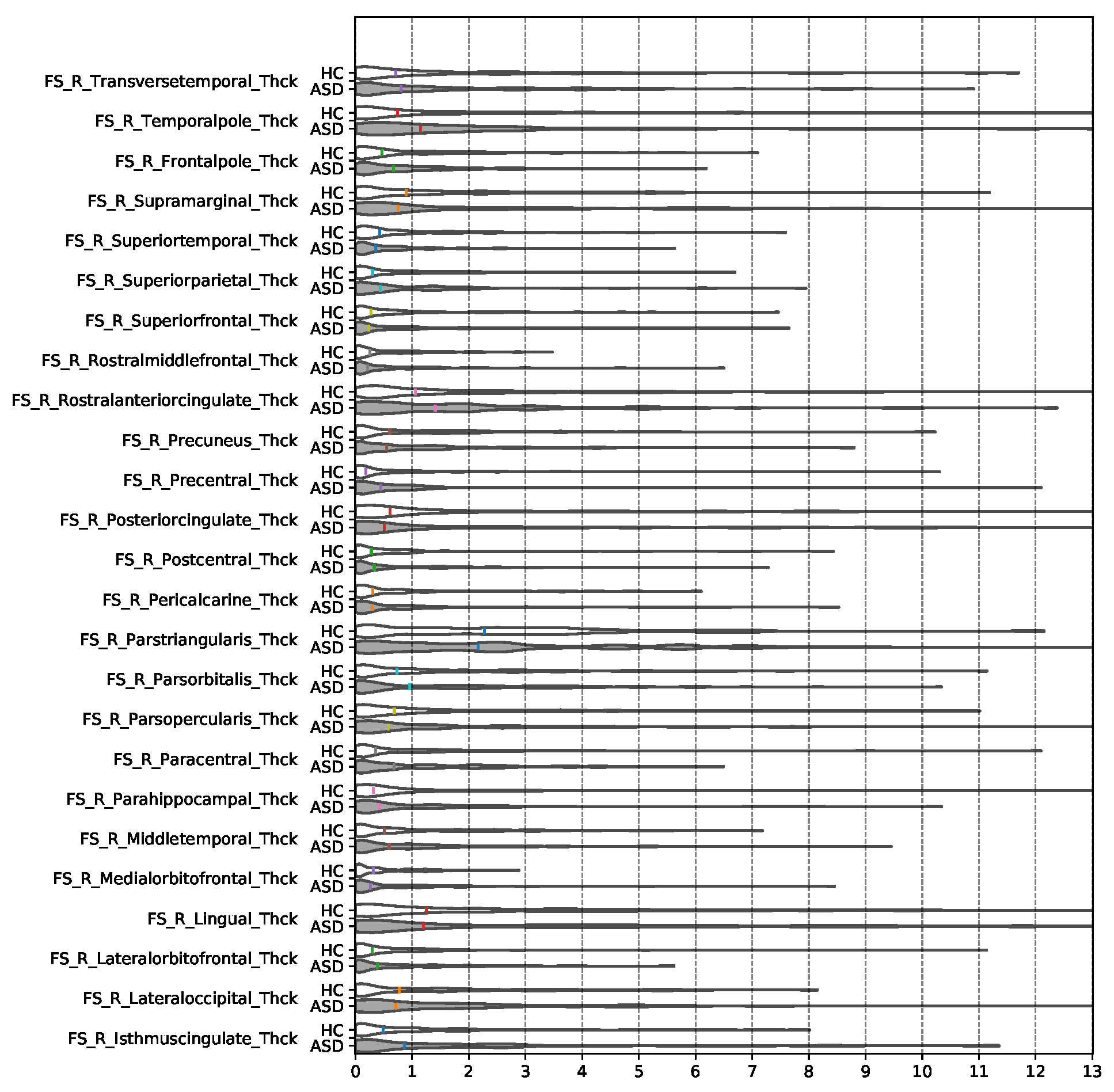
**

Figure 6 - Violin plot of the reconstruction error of each brain regions analyzed by the deep autoencoder using the ABIDE dataset. The medians of the distributions are indicated by the red line.

# Statistical significance and effect sizes of each region from the NUSDAST dataset

Table 2 - Statistical significance measured by the Mann-Whitney U test and effect size measured by Cliff’s delta absolute value based on the comparison of the reconstruction error of each brain of the groups from NUSDAST dataset. The significant regions (alpha >= 0.01) are highlighted in bold.

| **Regions** | **p-value** | **Effect size** | **Regions** | **p-value** | **Effect size** |
| --- | --- | --- | --- | --- | --- |
| **Left-Lateral-Ventricle** | **.0011** | **.4100** | lh_parsopercularis | .4472 | .0185 |
| Left-Inf-Lat-Vent | .2302 | .1000 | lh_parsorbitalis | .1502 | .1400 |
| Left-Cerebellum-White-Matter | .0276 | .2585 | lh_parstriangularis | .4640 | .0128 |
| Left-Cerebellum-Cortex | .1080 | .1671 | lh_pericalcarine | .1358 | .1485 |
| Left-Thalamus-Proper | .0256 | .2628 | lh_postcentral | .1603 | .1342 |
| Left-Caudate | .1876 | .1200 | lh_posteriorcingulate | .3413 | .0557 |
| Left-Putamen | .4014 | .0342 | **lh_precentral** | **.0090** | **.3185** |
| Left-Pallidum | .0515 | .2200 | lh_precuneus | .0403 | .2357 |
| 3rd-Ventricle | .0334 | .2471 | lh_rostralanteriorcingulate | .0637 | .2057 |
| 4th-Ventricle | .2958 | .0728 | lh_rostralmiddlefrontal | .1552 | .1371 |
| Brain-Stem | .0161 | .2885 | lh_superiorfrontal | .4014 | .0342 |
| Left-Hippocampus | .2849 | .0771 | lh_superiorparietal | .0966 | .1757 |
| Left-Amygdala | .2400 | .0957 | lh_superiortemporal | .1181 | .1600 |
| CSF | .2778 | .0800 | lh_supramarginal | .3730 | .0442 |
| Left-Accumbens-area | .0104 | .3114 | lh_frontalpole | .3297 | .0600 |
| **Left-VentralDC** | **.0009** | **.4171** | lh_temporalpole | .3891 | .0385 |
| Left-choroid-plexus | .4640 | .0128 | lh_transversetemporal | .3374 | .0571 |
| **Right-Lateral-Ventricle** | **.0074** | **.3285** | lh_insula | .0765 | .1928 |
| Right-Inf-Lat-Vent | .1763 | .1257 | rh_bankssts | .1290 | .1528 |
| Right-Cerebellum-White-Matter | .1003 | .1728 | rh_caudalanteriorcingulate | .4767 | .0085 |
| Right-Cerebellum-Cortex | .0226 | .2700 | rh_caudalmiddlefrontal | .0515 | .2200 |
| Right-Thalamus-Proper | .1429 | .1442 | rh_cuneus | .4388 | .0214 |
| Right-Caudate | .0549 | .2157 | rh_entorhinal | .2672 | .0842 |
| Right-Putamen | .1763 | .1257 | rh_fusiform | .1429 | .1442 |
| Right-Pallidum | .0157 | .2900 | rh_inferiorparietal | .4097 | .0314 |
| Right-Hippocampus | .1060 | .1685 | rh_inferiortemporal | .1290 | .1528 |
| Right-Amygdala | .3452 | .0542 | rh_isthmuscingulate | .1453 | .1428 |
| Right-Accumbens-area | .2707 | .0828 | rh_lateraloccipital | .2433 | .0942 |
| Right-VentralDC | .0130 | .3000 | rh_lateralorbitofrontal | .0692 | .2000 |
| Right-choroid-plexus | .0561 | .2142 | rh_lingual | .3452 | .0542 |
| Optic-Chiasm | .2144 | .1071 | rh_medialorbitofrontal | .0765 | .1928 |
| CC_Posterior | .0403 | .2357 | rh_middletemporal | .2602 | .0871 |
| CC_Mid_Posterior | .3182 | .0642 | rh_parahippocampal | .4514 | .0171 |
| CC_Central | .4388 | .0214 | rh_paracentral | .0948 | .1771 |
| CC_Mid_Anterior | .3689 | .0457 | rh_parsopercularis | .0845 | .1857 |
| CC_Anterior | .0895 | .1814 | rh_parsorbitalis | .3144 | .0657 |
| lh_bankssts | .2534 | .0900 | rh_parstriangularis | .3810 | .0414 |
| lh_caudalanteriorcingulate | .4472 | .0185 | rh_pericalcarine | .2534 | .0900 |
| lh_caudalmiddlefrontal | .2144 | .1071 | rh_postcentral | .1763 | .1257 |
| lh_cuneus | .1682 | .1300 | rh_posteriorcingulate | .0412 | .2342 |
| lh_entorhinal | .2602 | .0871 | rh_precentral | .3610 | .0485 |
| lh_fusiform | .1904 | .1185 | rh_precuneus | .2742 | .0814 |
| lh_inferiorparietal | .4851 | .0057 | rh_rostralanteriorcingulate | .2958 | .0728 |
| lh_inferiortemporal | .2885 | .0757 | rh_rostralmiddlefrontal | .1629 | .1328 |
| lh_isthmuscingulate | .0130 | .3000 | rh_superiorfrontal | .3491 | .0528 |
| lh_lateraloccipital | .1876 | .1200 | rh_superiorparietal | .4138 | .0300 |
| lh_lateralorbitofrontal | .1060 | .1685 | **rh_superiortemporal** | **.0020** | **.3871** |
| lh_lingual | .1791 | .1242 | rh_supramarginal | .4978 | .0014 |
| lh_medialorbitofrontal | .1080 | .1671 | rh_frontalpole | .4014 | .0342 |
| lh_middletemporal | .1992 | .1142 | rh_temporalpole | .3491 | .0528 |
| lh_parahippocampal | .0504 | .2214 | rh_transversetemporal | .3069 | .0685 |
| lh_paracentral | .3891 | .0385 | rh_insula | .4556 | .0157 |

# Statistical significance and effect sizes of each region from the ABIDE dataset

Table 3 - Statistical significance measured by the Mann-Whitney U test and effect size measured by Cliff’s delta absolute value based on the comparison of the reconstruction error of each brain of the groups from ABIDE dataset. The significant regions (alpha >= 0.01) are highlighted in bold.

| **Regions** | **p-value** | **Effect size** | **Regions** | **p-value** | **Effect size** |
| --- | --- | --- | --- | --- | --- |
| Left-Lateral-Ventricle | .2145 | 0674 | lh_parsopercularis | .4166 | .0180 |
| Left-Inf-Lat-Vent | .1636 | .0834 | lh_parsorbitalis | .3812 | .0258 |
| Left-Cerebellum-White-Matter | .0886 | .1149 | lh_parstriangularis | .1822 | .0772 |
| **Left-Cerebellum-Cortex** | **.0046** | **.2216** | lh_pericalcarine | .3699 | .0283 |
| Left-Thalamus-Proper | .4731 | .0059 | lh_postcentral | .2603 | .0547 |
| Left-Caudate | .0990 | .1096 | lh_posteriorcingulate | .1072 | .1057 |
| **Left-Putamen** | **.0037** | **.2280** | lh_precentral | .3988 | .0219 |
| Left-Pallidum | .0957 | .1112 | lh_precuneus | .2559 | .0559 |
| 3rd-Ventricle | .1475 | .0892 | lh_rostralanteriorcingulate | .0217 | .1718 |
| 4th-Ventricle | .4957 | .0010 | lh_rostralmiddlefrontal | .2799 | .0497 |
| Brain-Stem | .3802 | .0260 | lh_superiorfrontal | .1773 | .0788 |
| Left-Hippocampus | .2021 | .0710 | lh_superiorparietal | .1649 | .0830 |
| Left-Amygdala | .1438 | .0905 | lh_superiortemporal | .3478 | .0334 |
| CSF | .3078 | .0428 | lh_supramarginal | .3578 | .0311 |
| Left-Accumbens-area | .1378 | .0928 | lh_frontalpole | .4082 | .0199 |
| Left-VentralDC | .1724 | .0804 | lh_temporalpole | .1738 | .0800 |
| **Left-choroid-plexus** | **.0017** | **.2496** | lh_transversetemporal | .3300 | .0375 |
| Right-Lateral-Ventricle | .0966 | .1107 | lh_insula | .3792 | .0263 |
| Right-Inf-Lat-Vent | .1801 | .0779 | rh_bankssts | .2533 | .0566 |
| Right-Cerebellum-White-Matter | .3203 | .0398 | rh_caudalanteriorcingulate | .1551 | .0864 |
| Right-Cerebellum-Cortex | .1710 | .0809 | rh_caudalmiddlefrontal | .3222 | .0394 |
| Right-Thalamus-Proper | .0547 | .1362 | **rh_cuneus** | **.0020** | **.2448** |
| Right-Caudate | .2241 | .0646 | rh_entorhinal | .1164 | .1015 |
| Right-Putamen | .0180 | .1784 | rh_fusiform | .4817 | .0040 |
| Right-Pallidum | .2456 | .0586 | rh_inferiorparietal | .2542 | .0563 |
| Right-Hippocampus | .2551 | .0561 | rh_inferiortemporal | .1643 | .0832 |
| Right-Amygdala | .0222 | .1711 | rh_isthmuscingulate | .4699 | .0065 |
| Right-Accumbens-area | .3021 | .0442 | rh_lateraloccipital | .0104 | .1966 |
| Right-VentralDC | .0688 | .1263 | rh_lateralorbitofrontal | .2700 | .0522 |
| Right-choroid-plexus | .1360 | .0935 | rh_lingual | .1703 | .0811 |
| Optic-Chiasm | .3999 | .0217 | rh_medialorbitofrontal | .2718 | .0517 |
| CC_Posterior | .3003 | .0446 | rh_middletemporal | .4072 | .0201 |
| CC_Mid_Posterior | .4527 | .0102 | rh_parahippocampal | .3967 | .0224 |
| CC_Central | .1616 | .0841 | rh_paracentral | .0740 | .1231 |
| CC_Mid_Anterior | .1513 | .0878 | rh_parsopercularis | .0217 | .1718 |
| CC_Anterior | .2298 | .0630 | rh_parsorbitalis | .3174 | .0405 |
| lh_bankssts | .3528 | .0322 | rh_parstriangularis | .3628 | .0299 |
| lh_caudalanteriorcingulate | .0613 | .1314 | rh_pericalcarine | .4527 | .0102 |
| lh_caudalmiddlefrontal | .2709 | .0520 | rh_postcentral | .3040 | .0437 |
| lh_cuneus | .1251 | .0979 | rh_posteriorcingulate | .2674 | .0529 |
| lh_entorhinal | .0562 | .1351 | rh_precentral | .4357 | .0139 |
| lh_fusiform | .1112 | .1038 | rh_precuneus | .0445 | .1447 |
| lh_inferiorparietal | .3031 | .0439 | rh_rostralanteriorcingulate | .4517 | .0104 |
| lh_inferiortemporal | .2098 | .0687 | rh_rostralmiddlefrontal | .1939 | .0736 |
| lh_isthmuscingulate | .4613 | .0084 | rh_superiorfrontal | .4314 | .0148 |
| lh_lateraloccipital | .3853 | .0249 | rh_superiorparietal | .3031 | .0439 |
| lh_lateralorbitofrontal | .3116 | .0419 | rh_superiortemporal | .2665 | .0531 |
| lh_lingual | .4860 | .0031 | rh_supramarginal | .1290 | .0963 |
| lh_medialorbitofrontal | .1112 | .1038 | rh_frontalpole | .4817 | .0040 |
| lh_middletemporal | .4806 | .0042 | rh_temporalpole | .2612 | .0545 |
| lh_parahippocampal | .3164 | .0407 | rh_transversetemporal | .0227 | .1702 |
| lh_paracentral | .2937 | .0462 | rh_insula | .3874 | .0244 |

# Mean original and reconstructed values of each region

Here, for each dataset, we reported the mean original and reconstructed values in each brain region and in each group, with the error bars representing the standard deviation. For each dataset, the healthy control group is shown in black and the patient group is shown in red. A dashed horizontal line is used to help compare the original and reconstructed values. The original values were normalized using the Human Connectome Project statistics (mean and standard deviation).


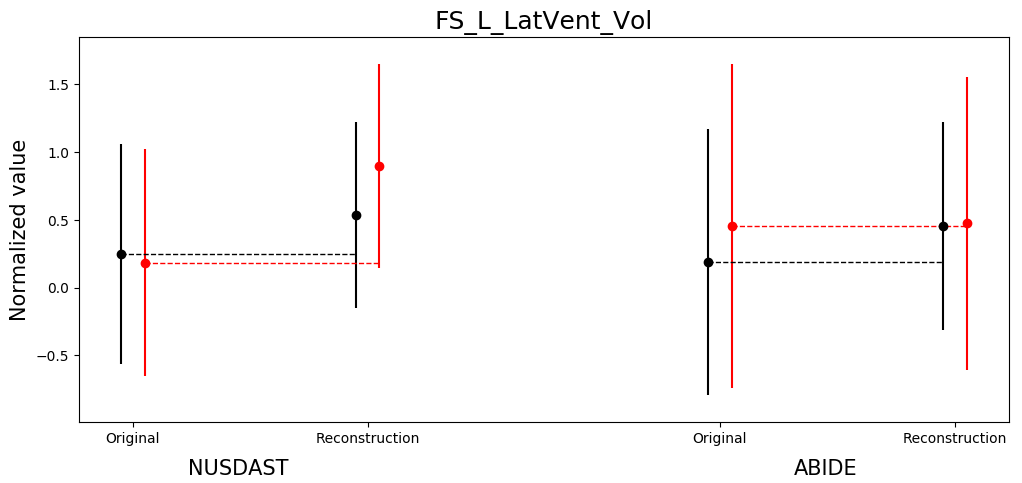

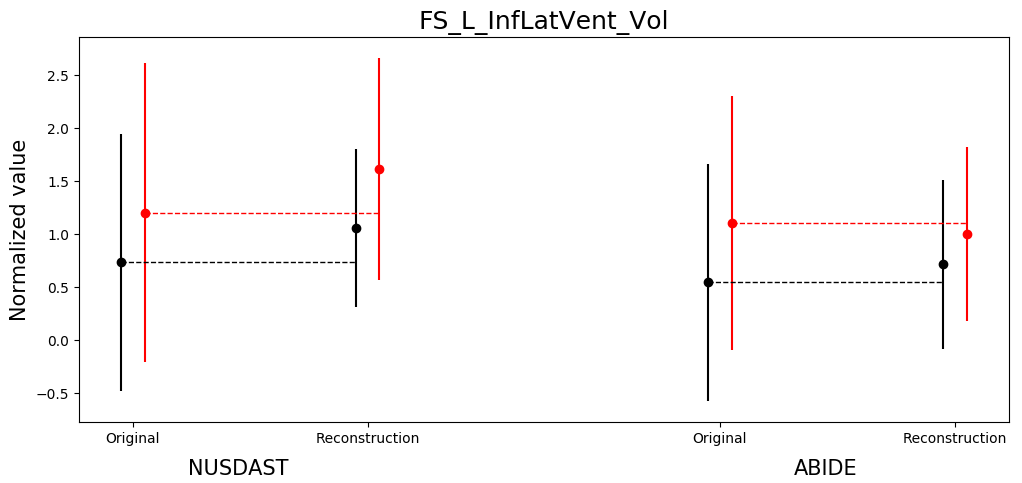

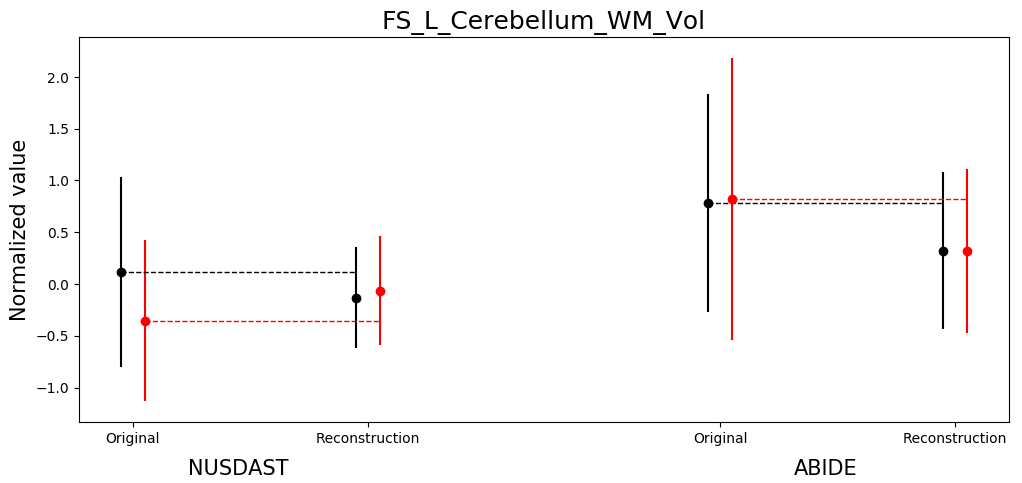

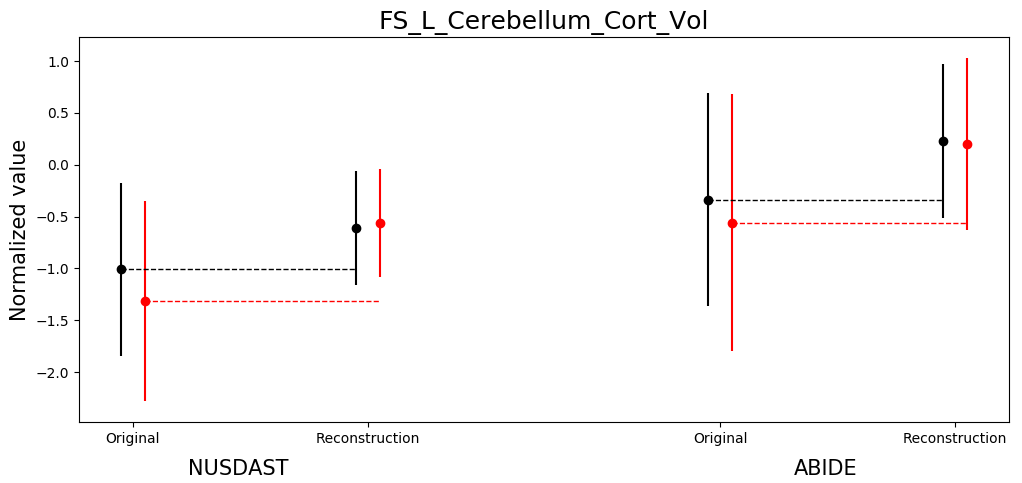

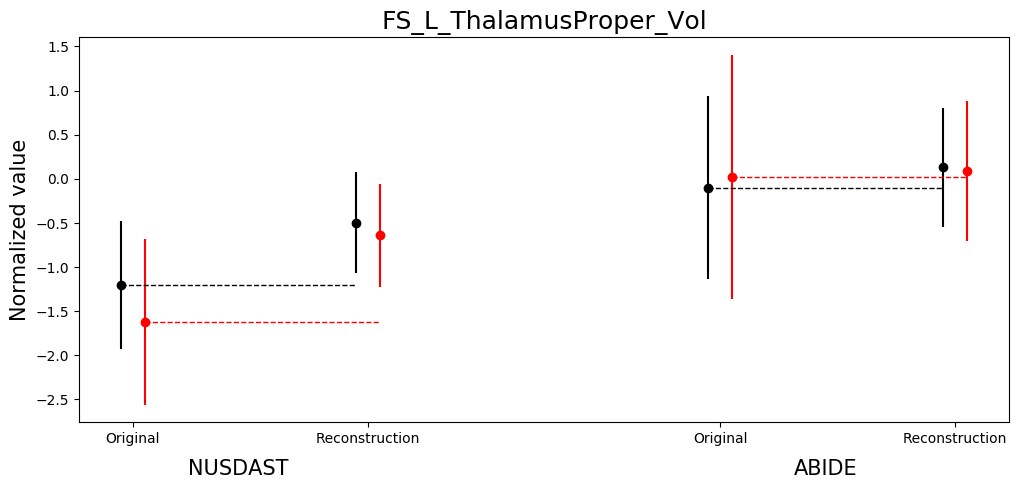

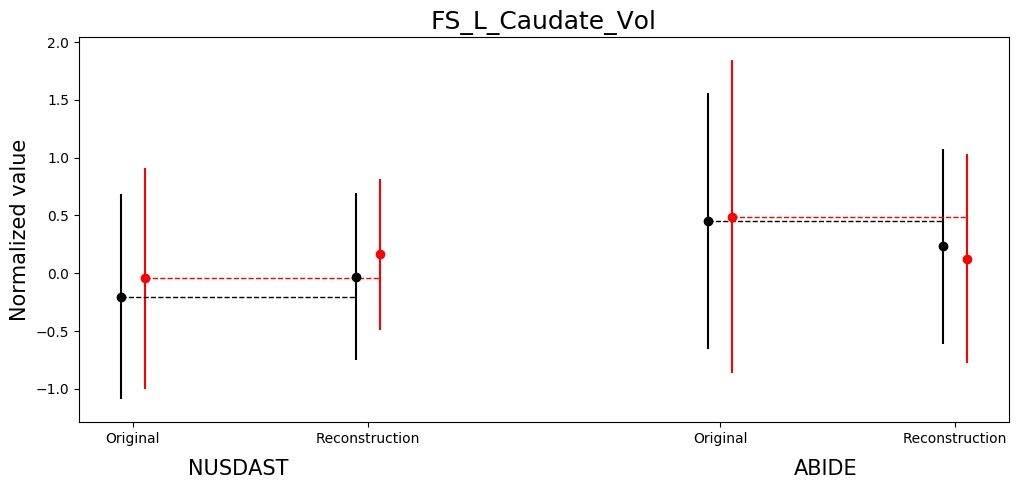

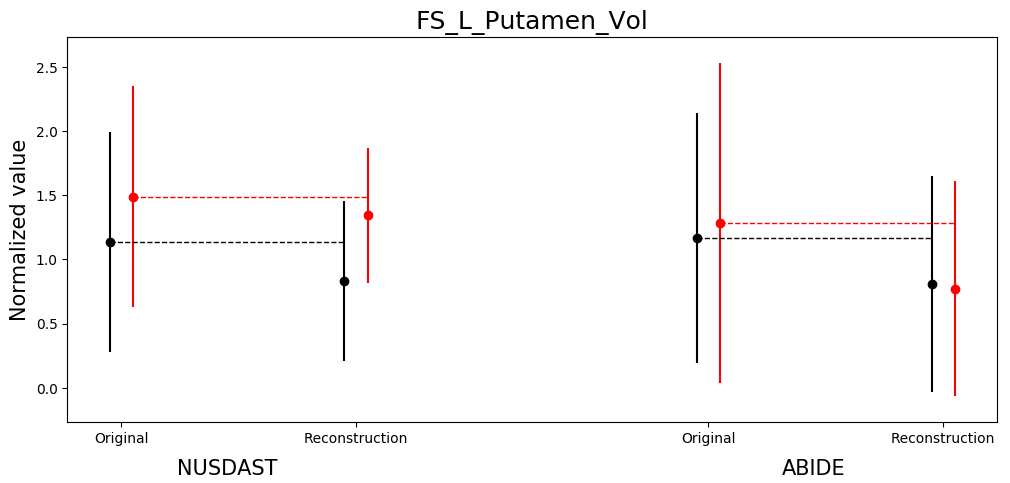

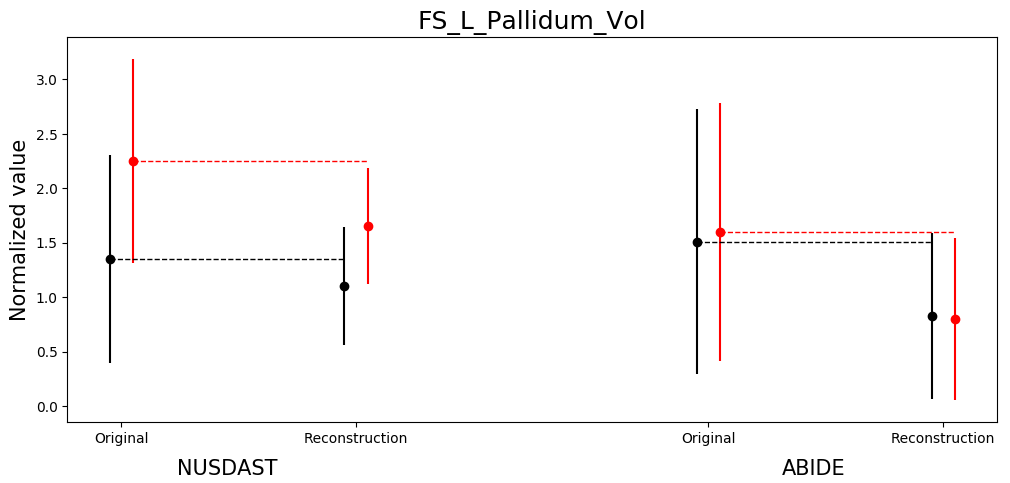

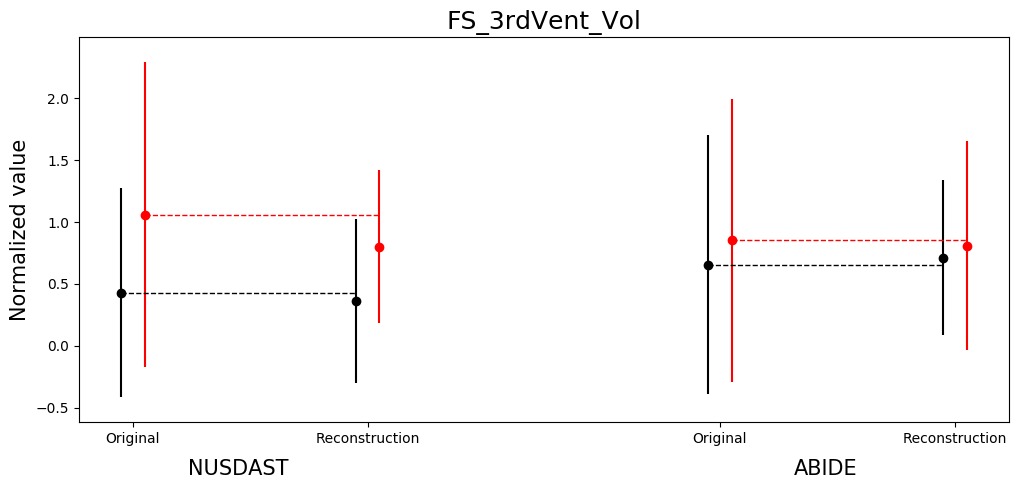

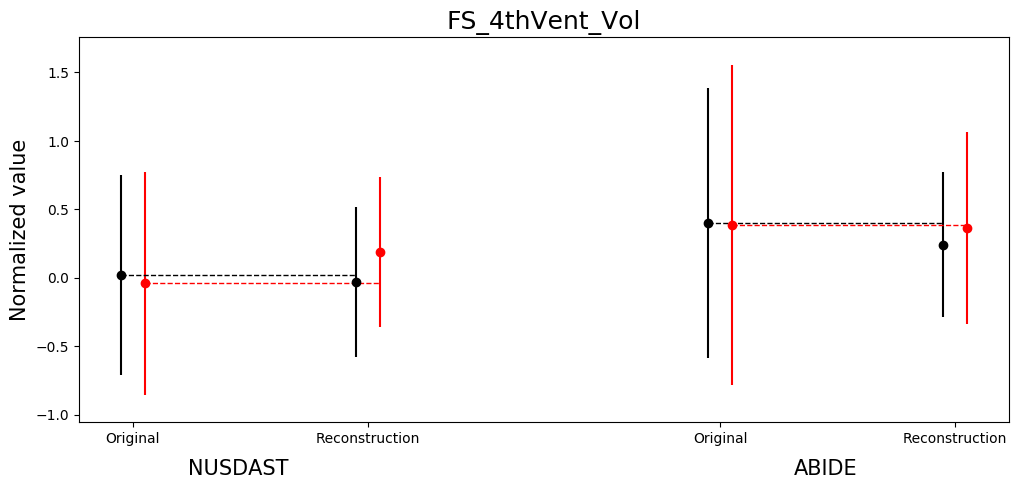

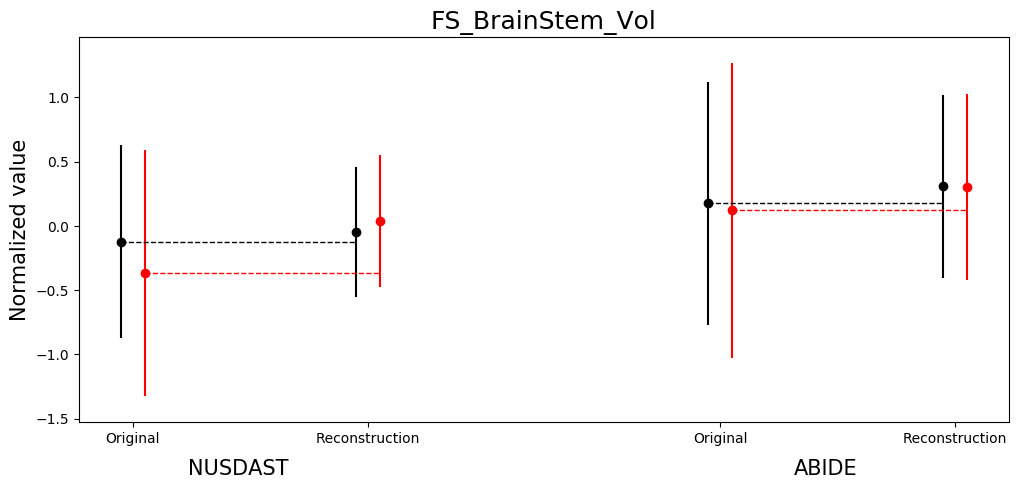

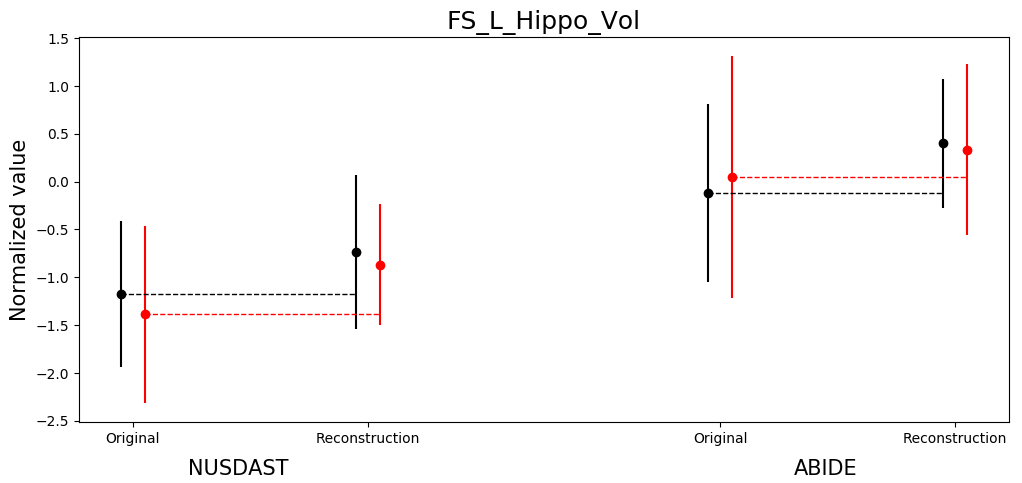

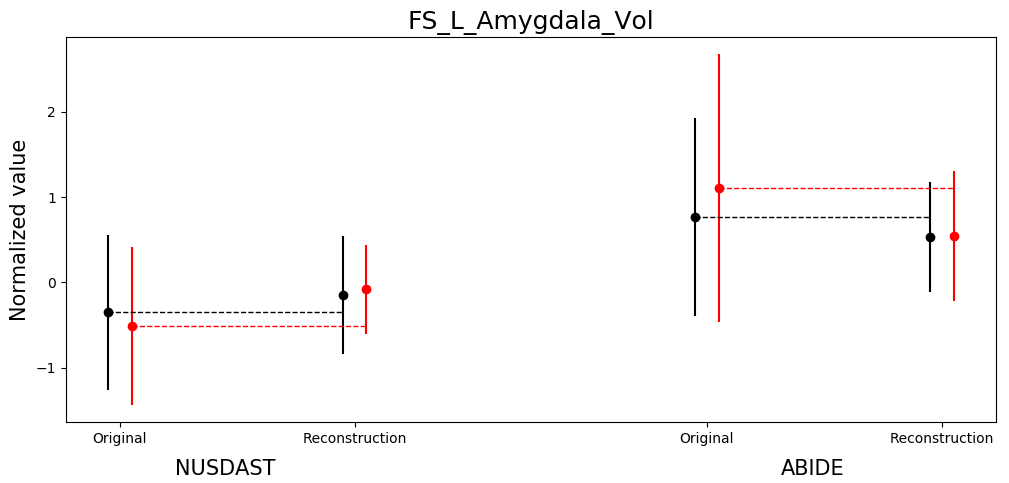

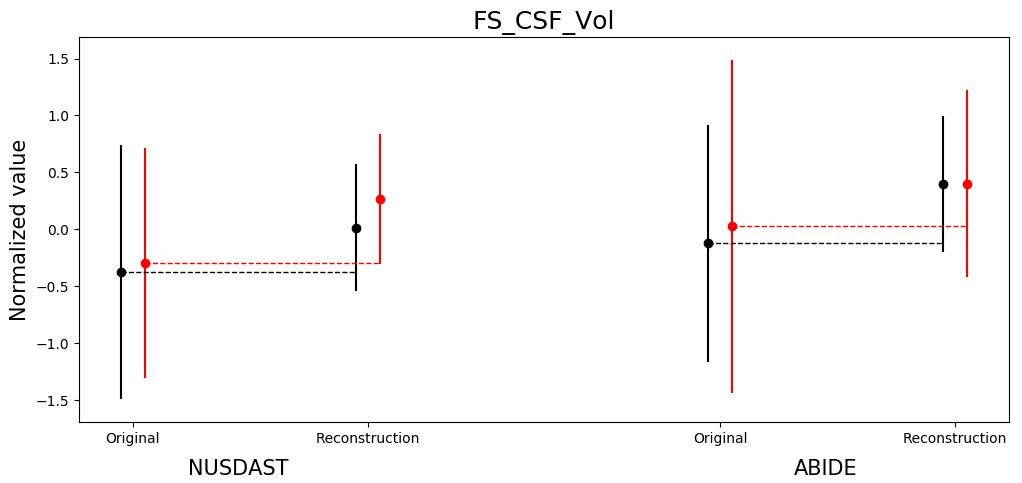

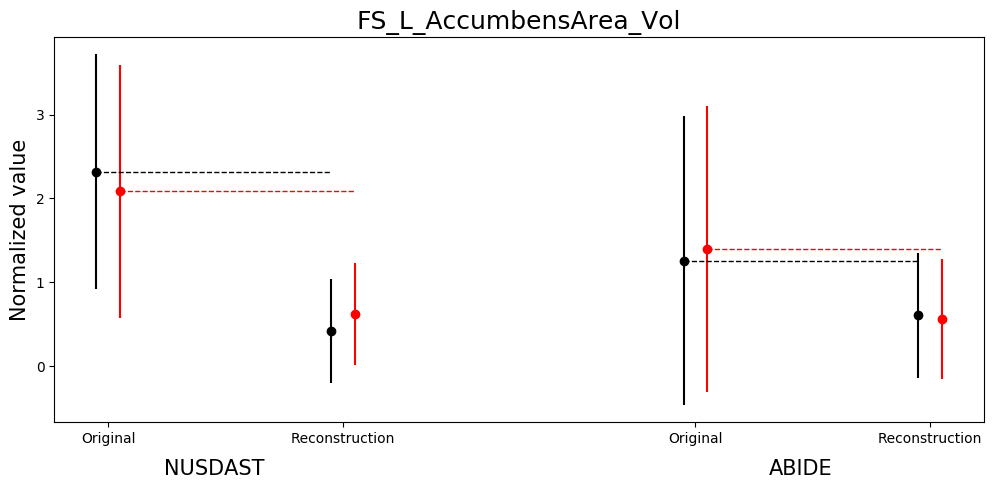

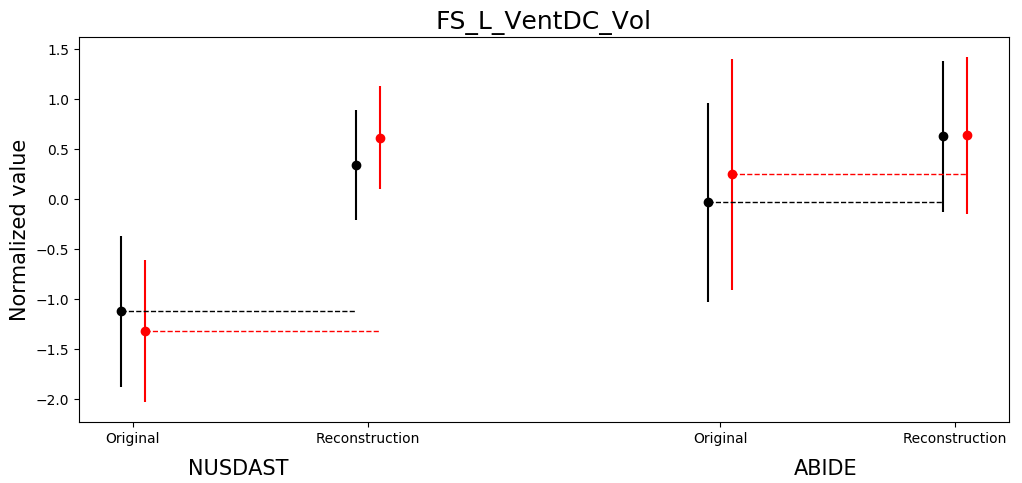

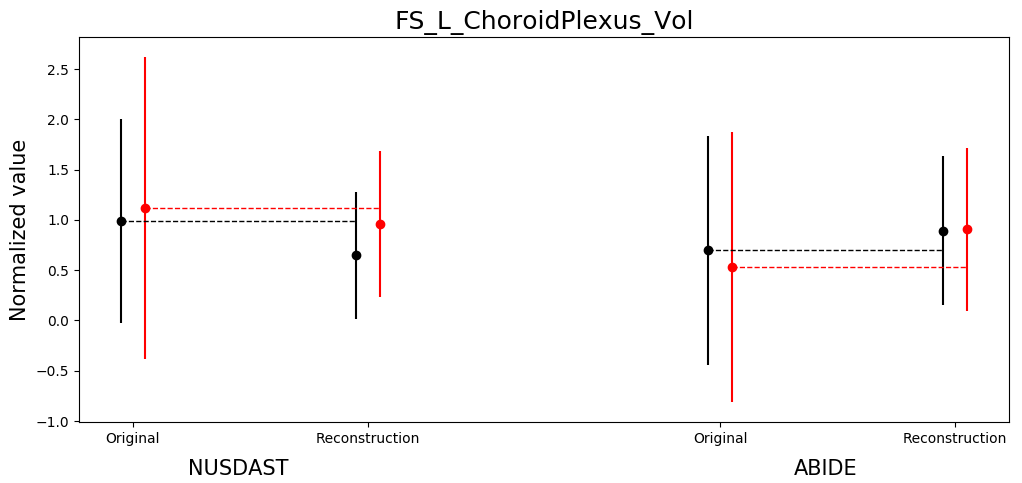

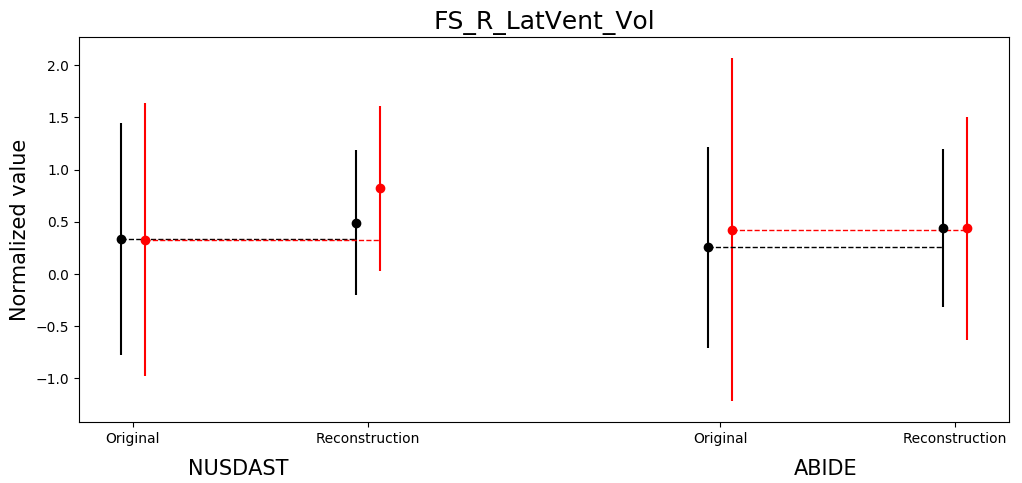

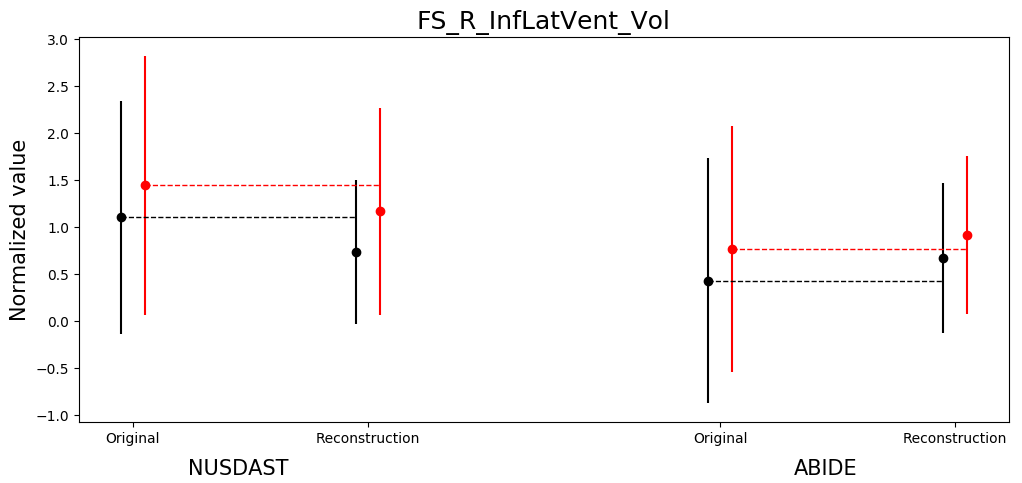

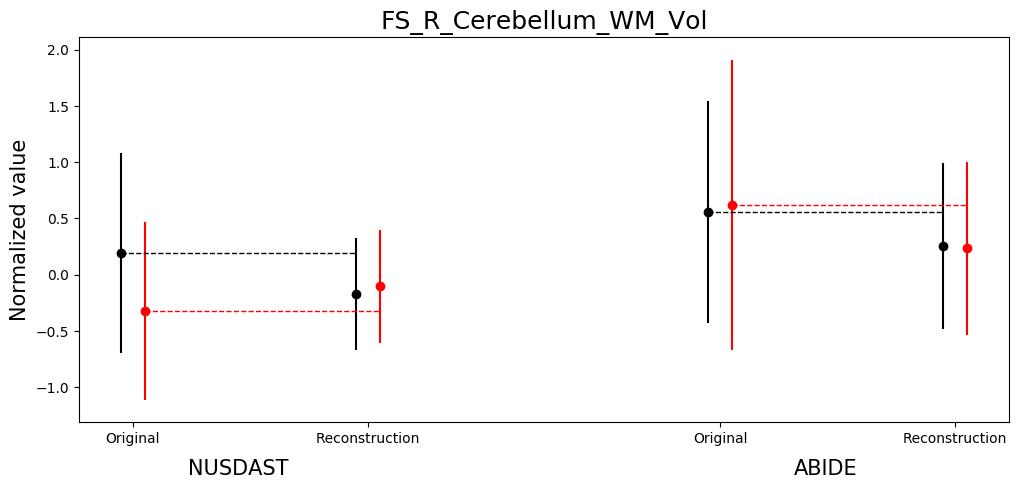

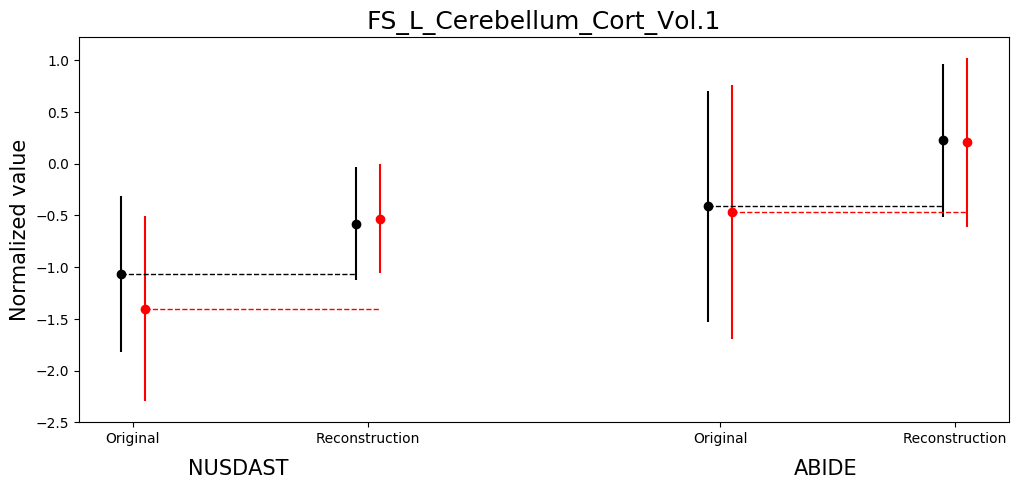

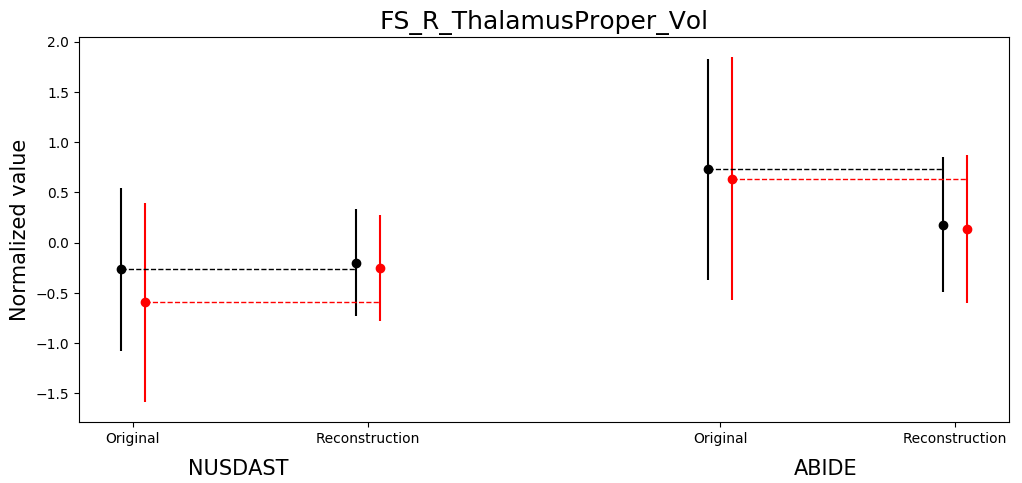

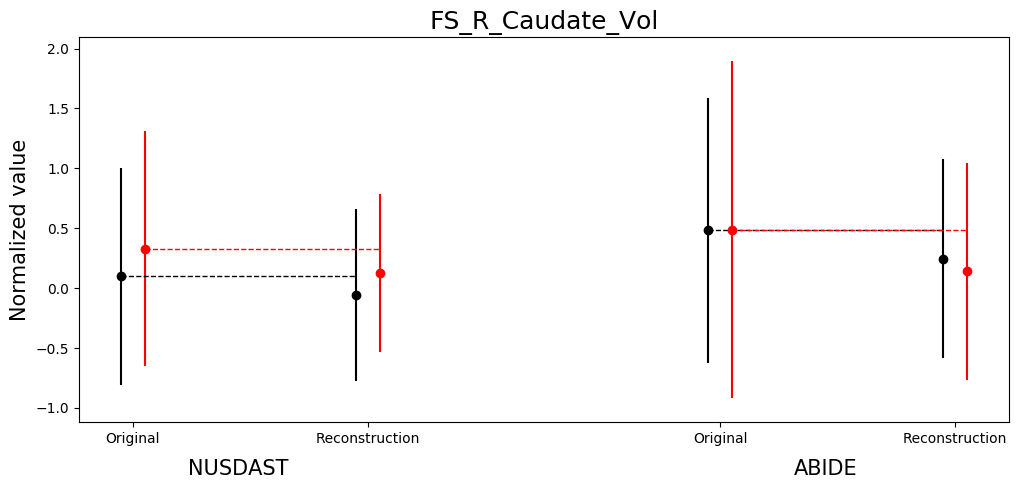

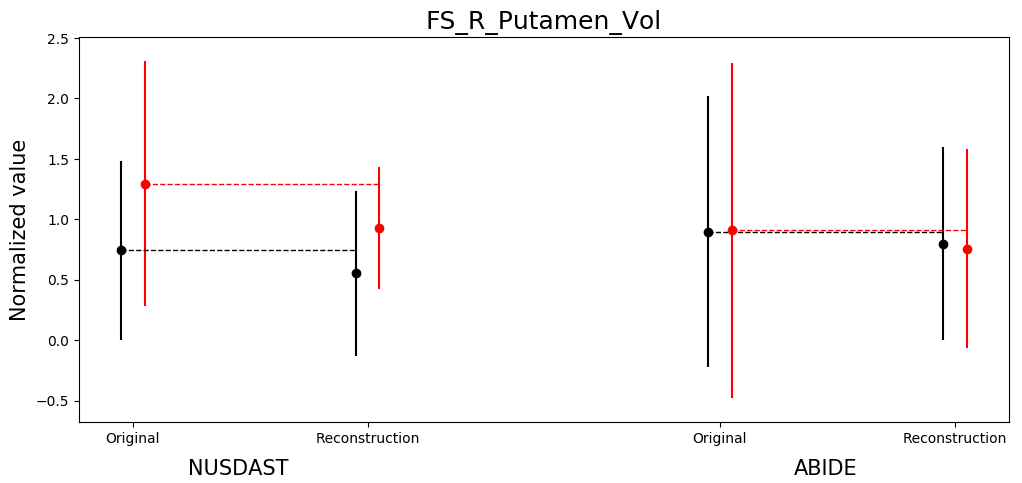

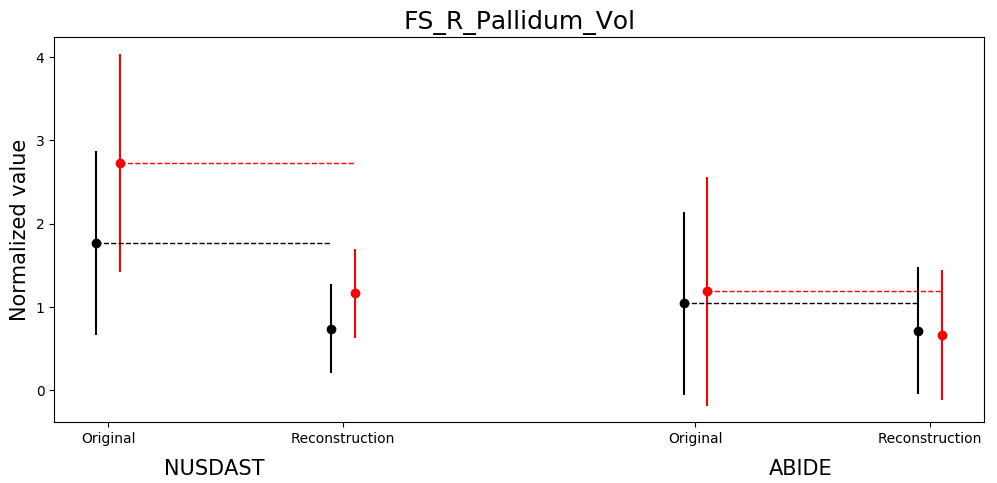

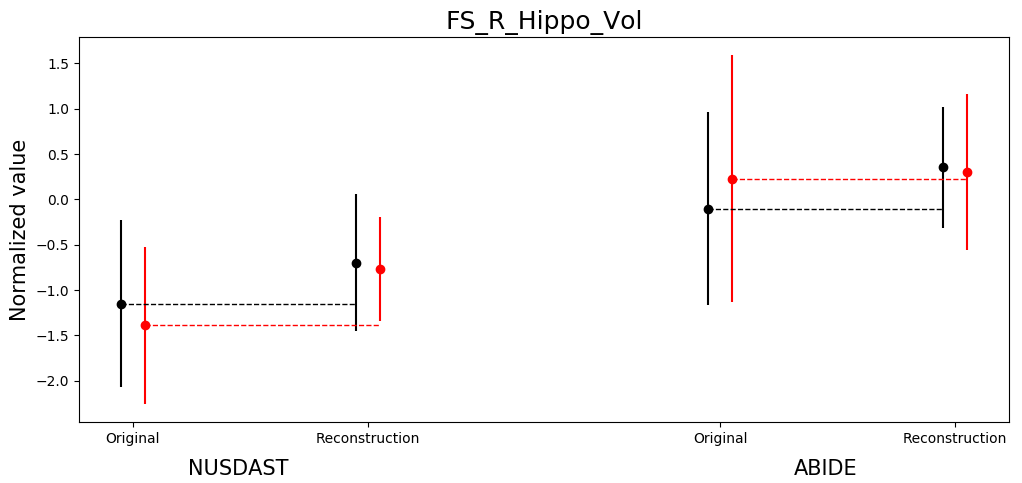

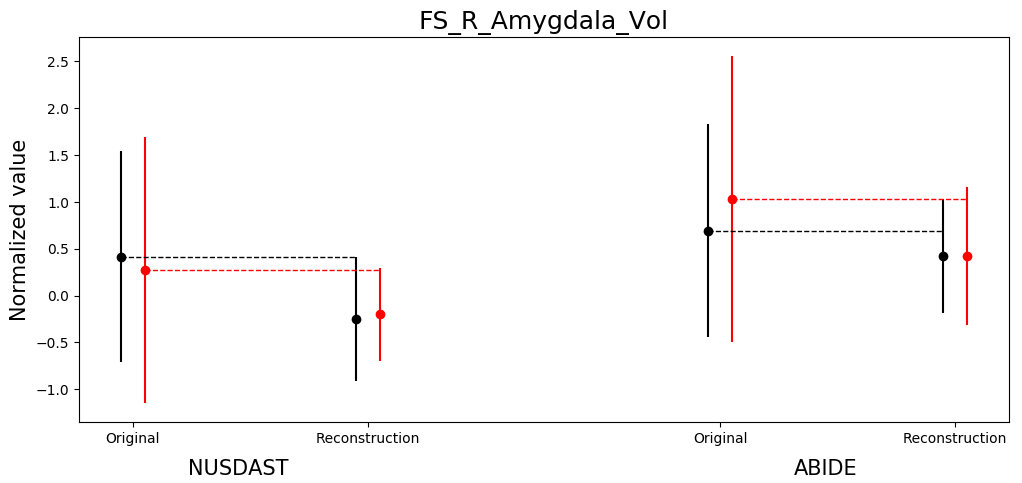

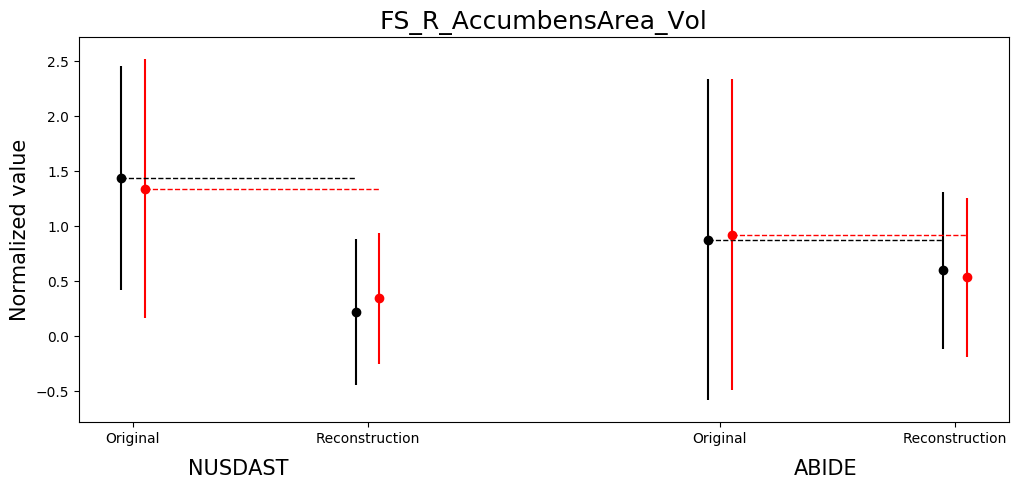

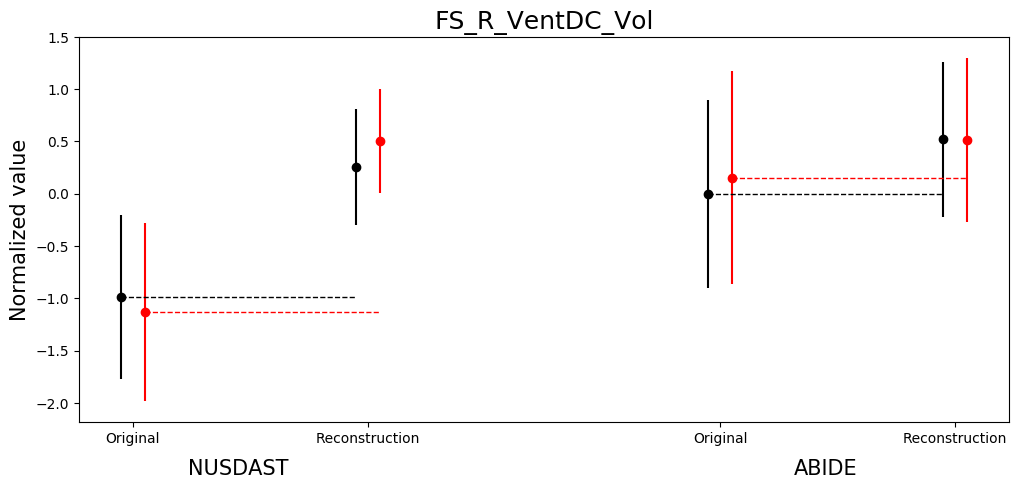

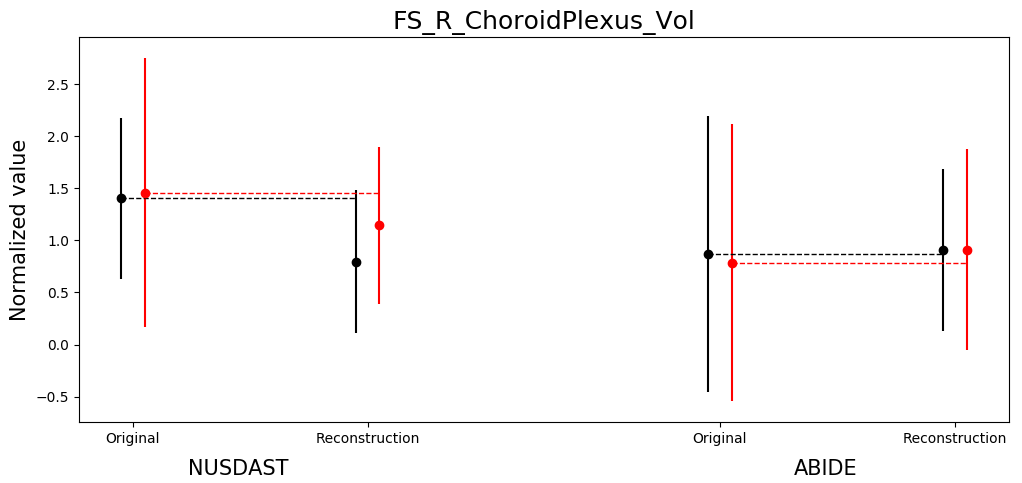

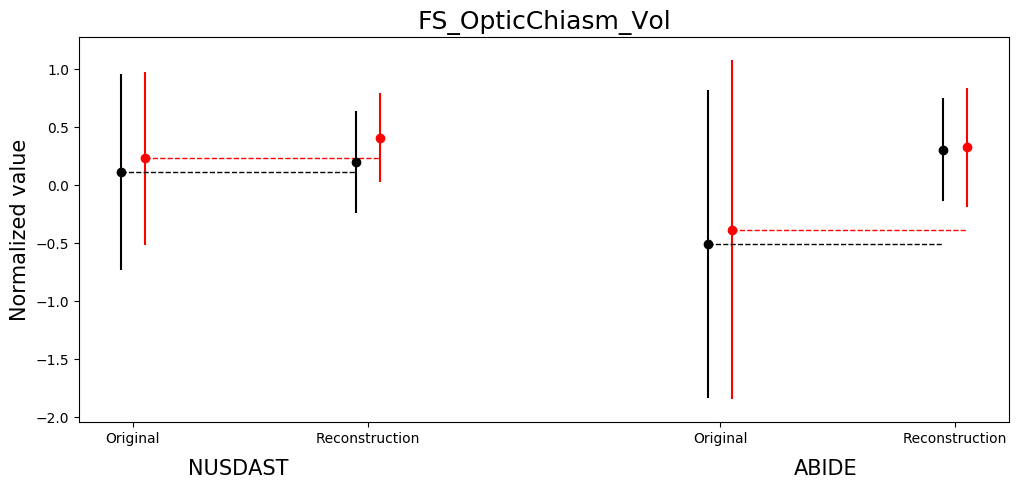

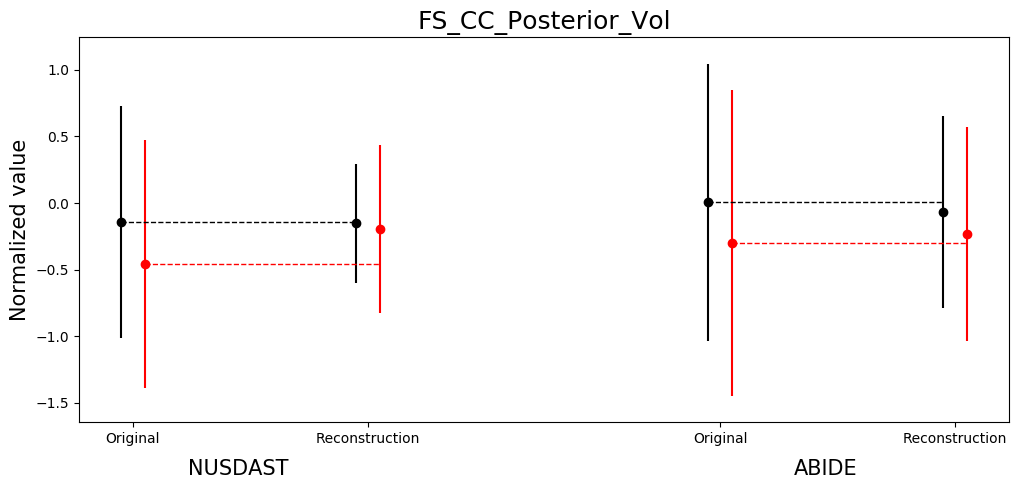

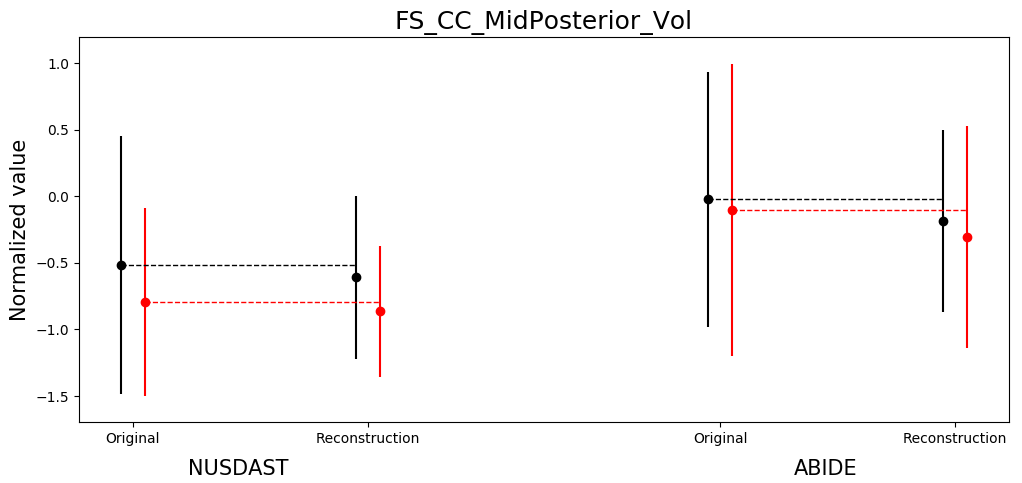

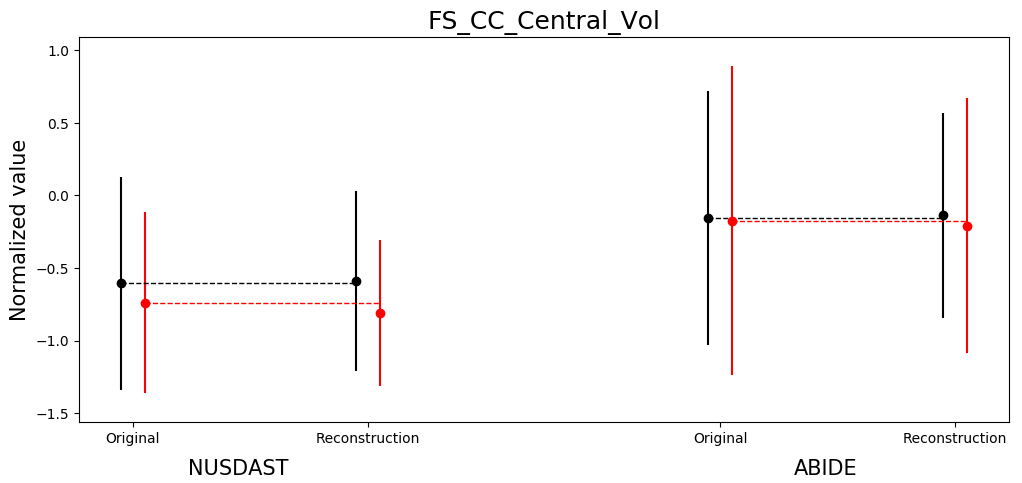

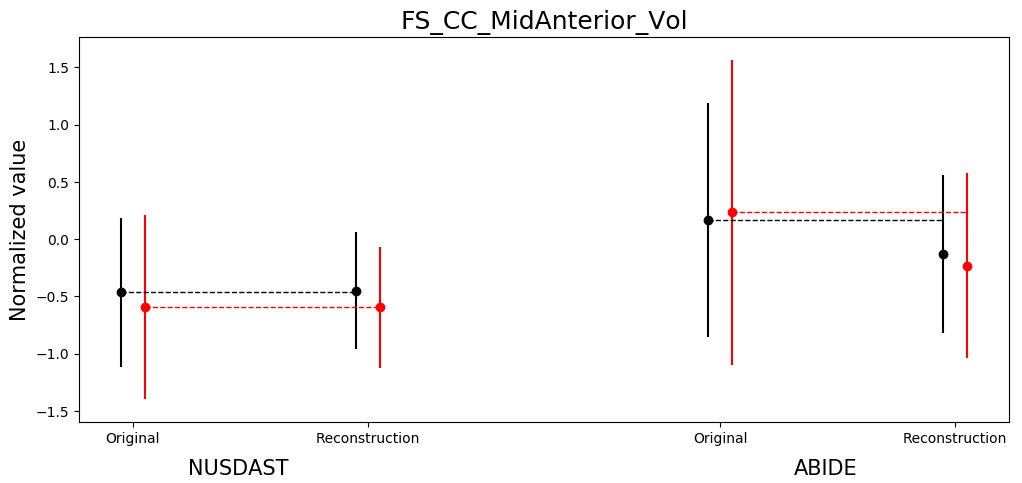

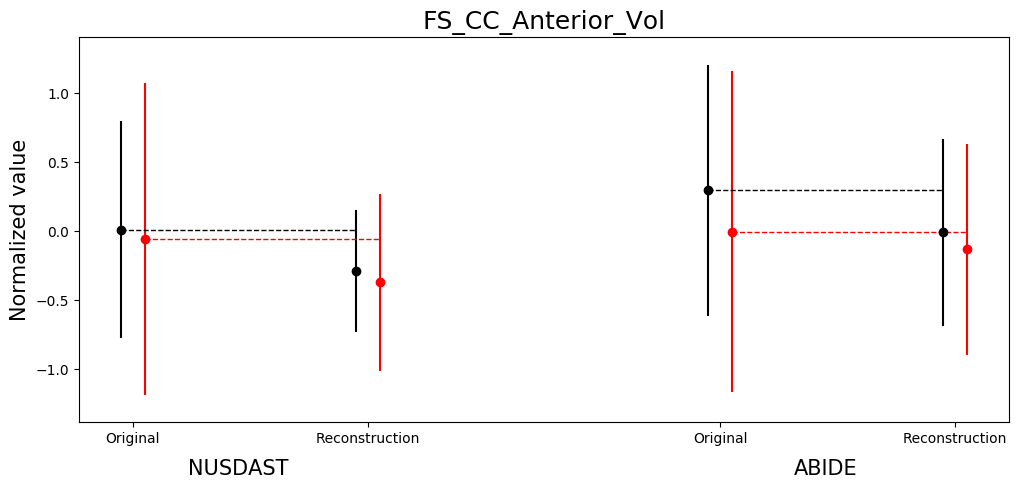

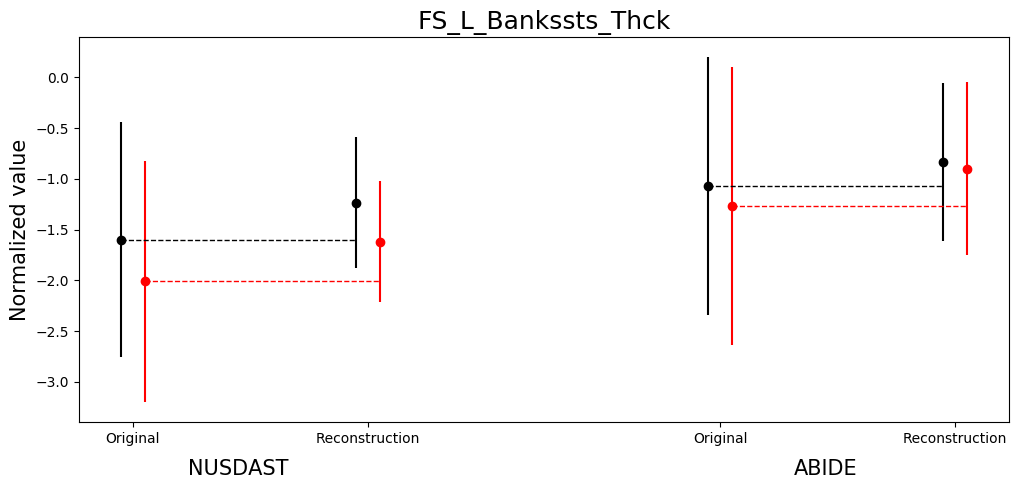

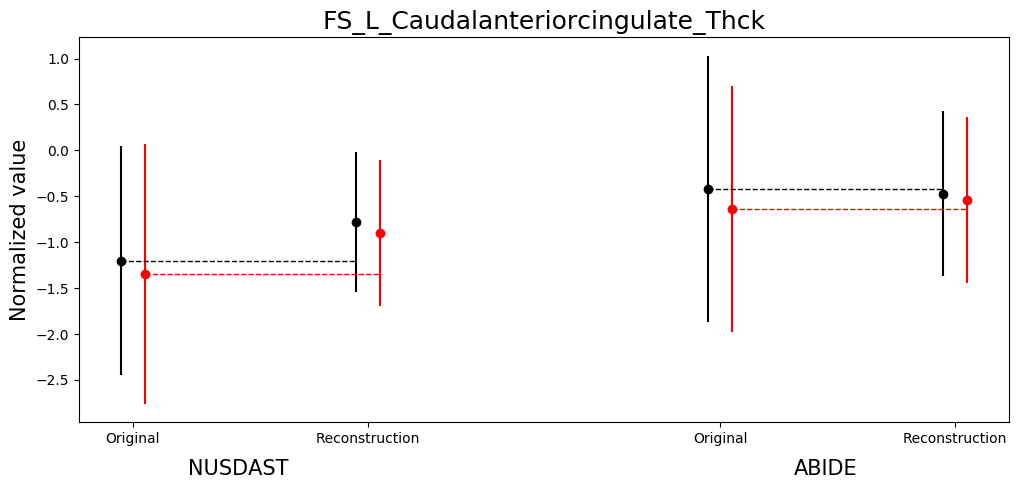

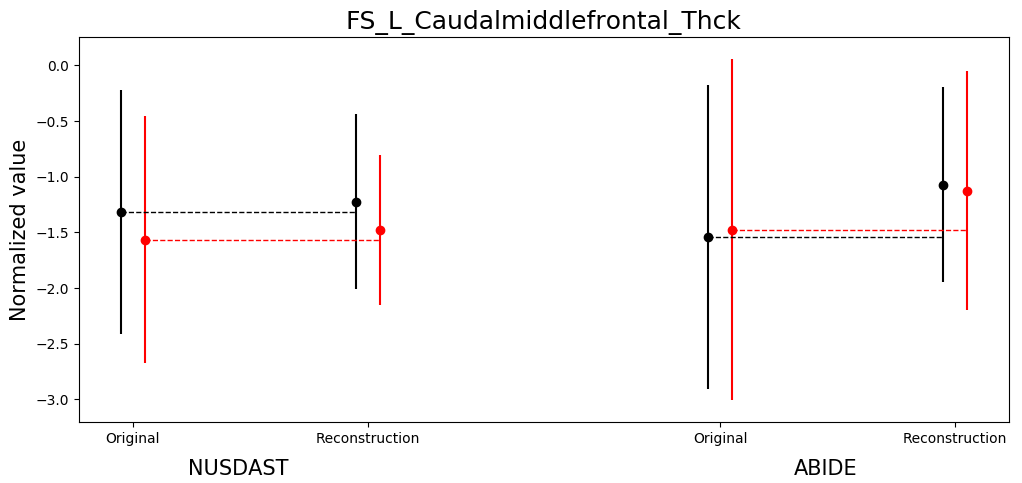

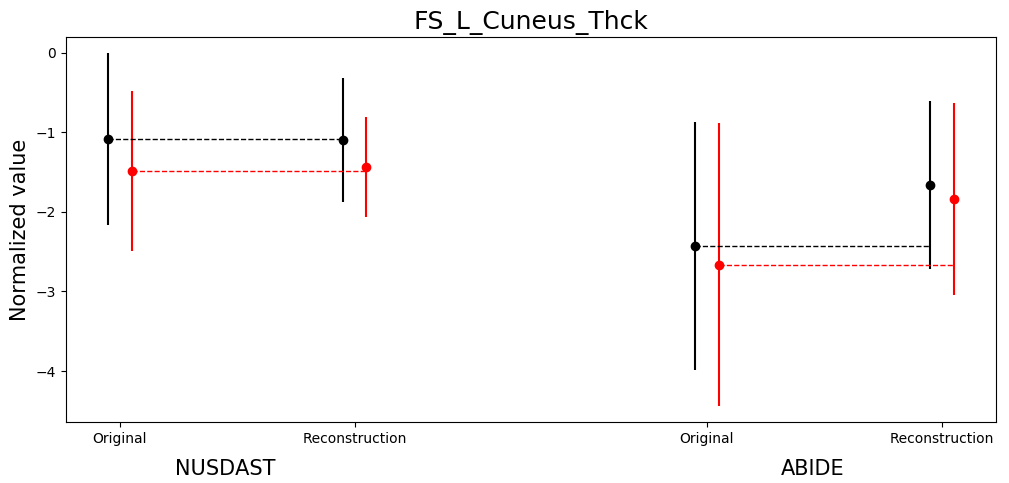

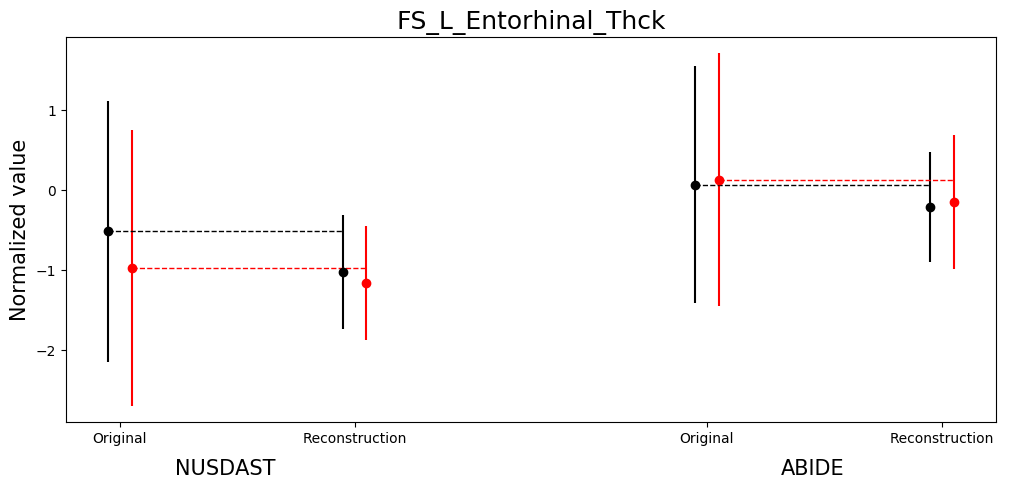

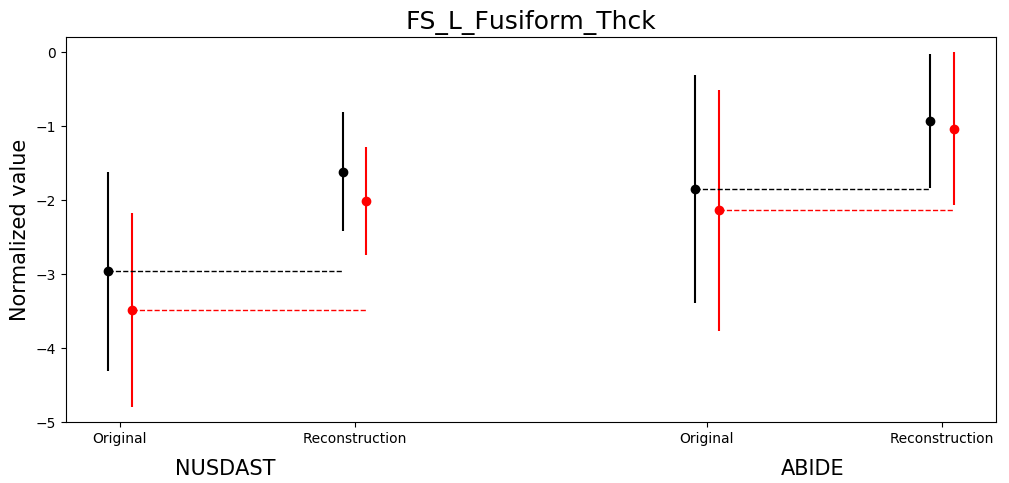

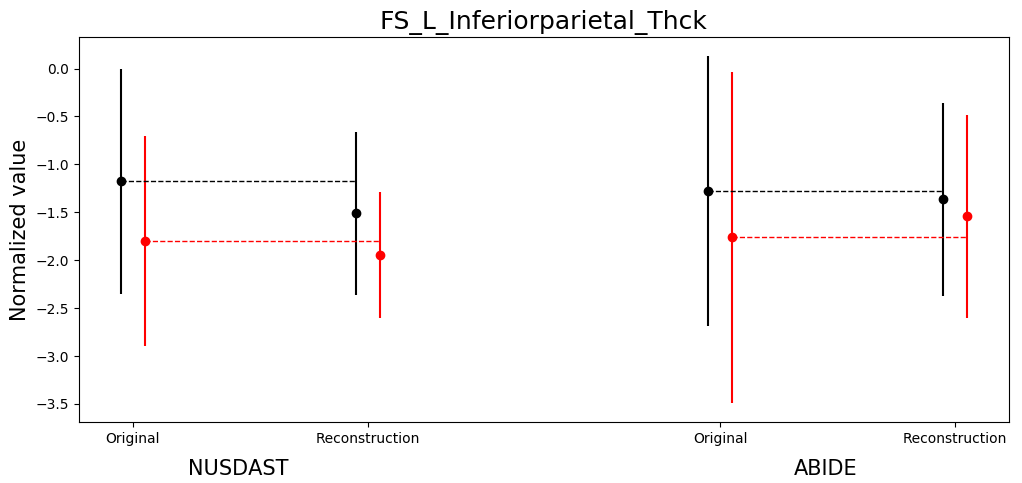

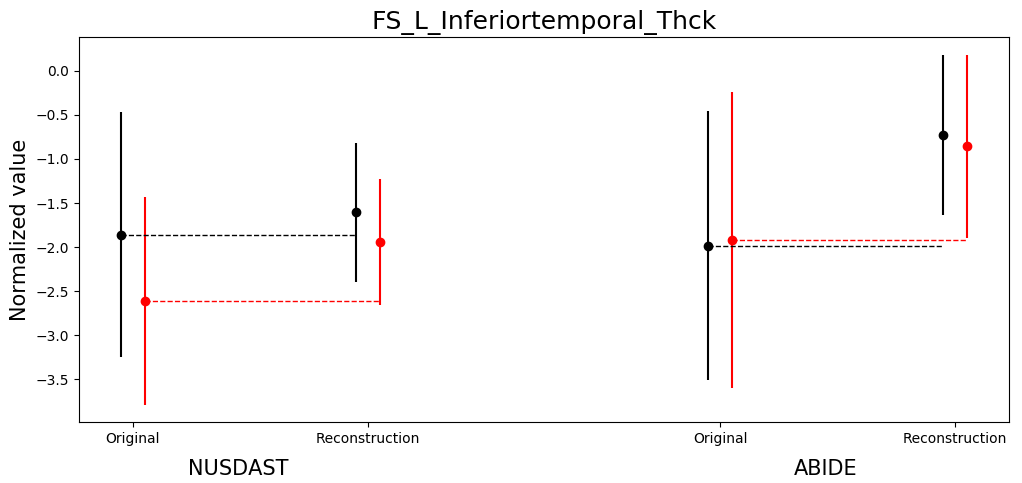

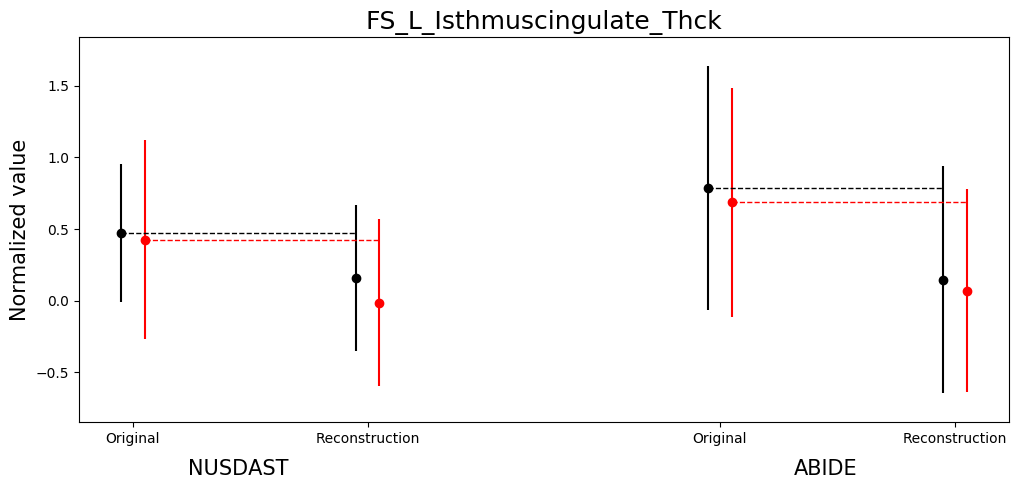

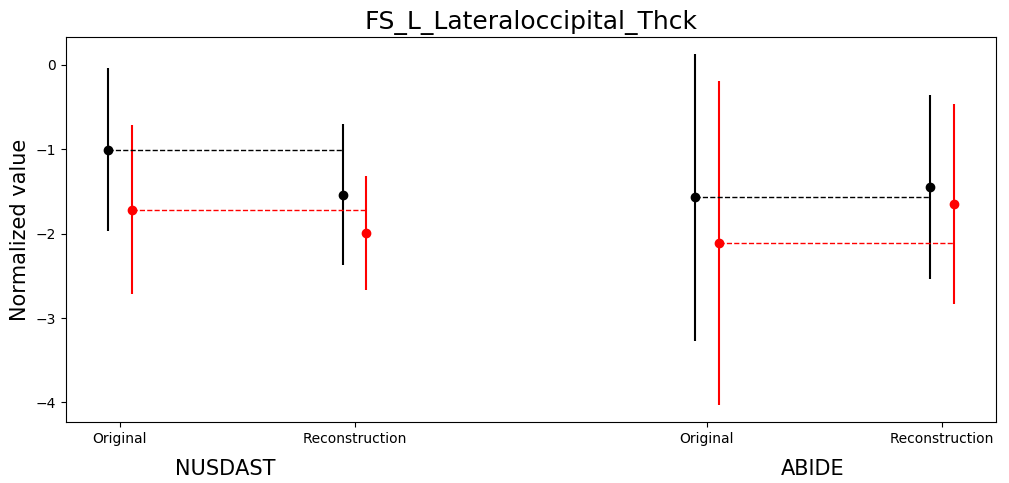

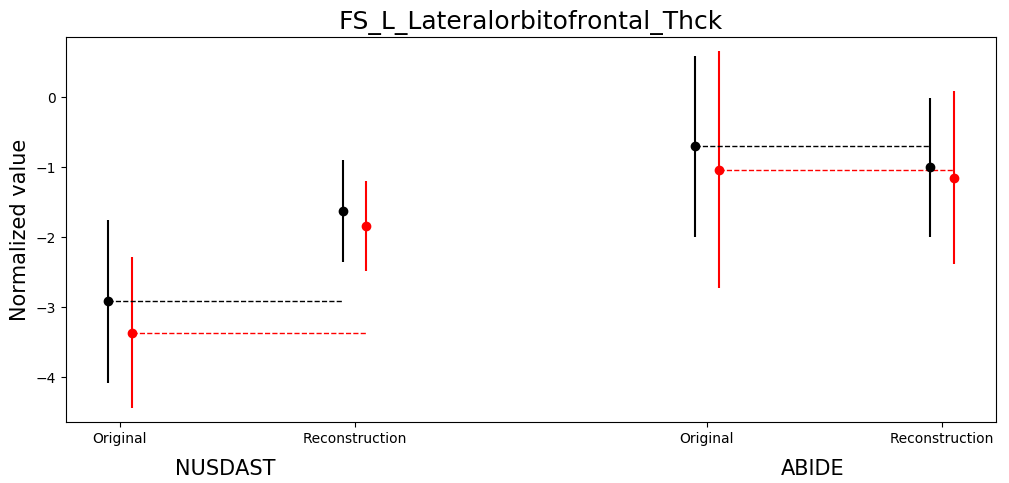

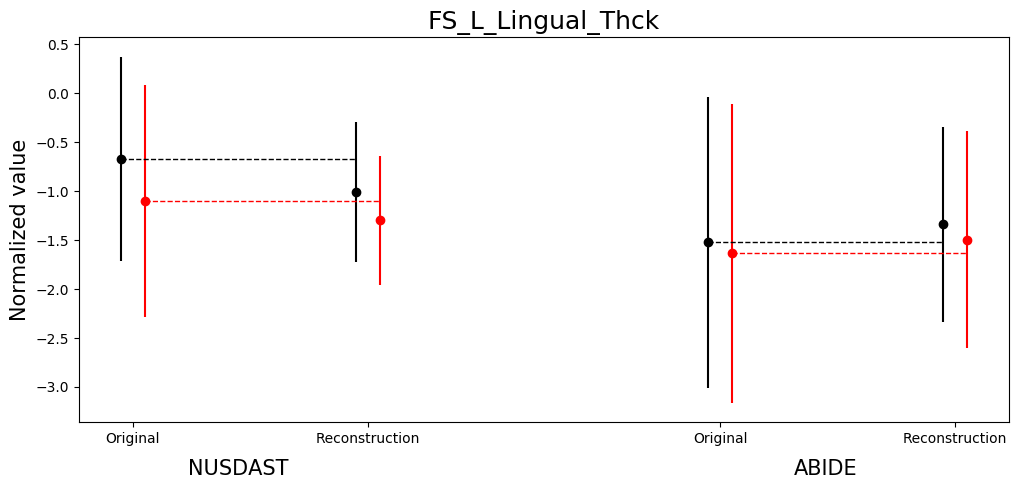

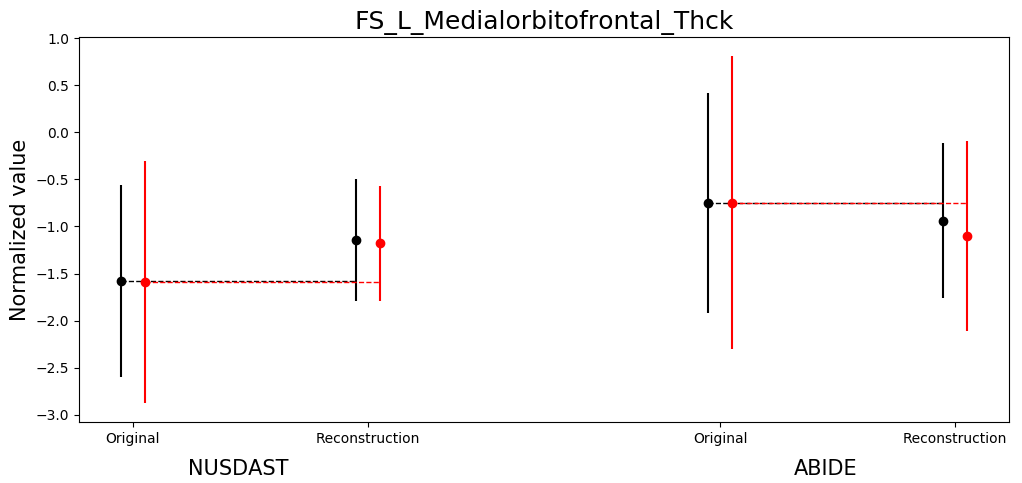

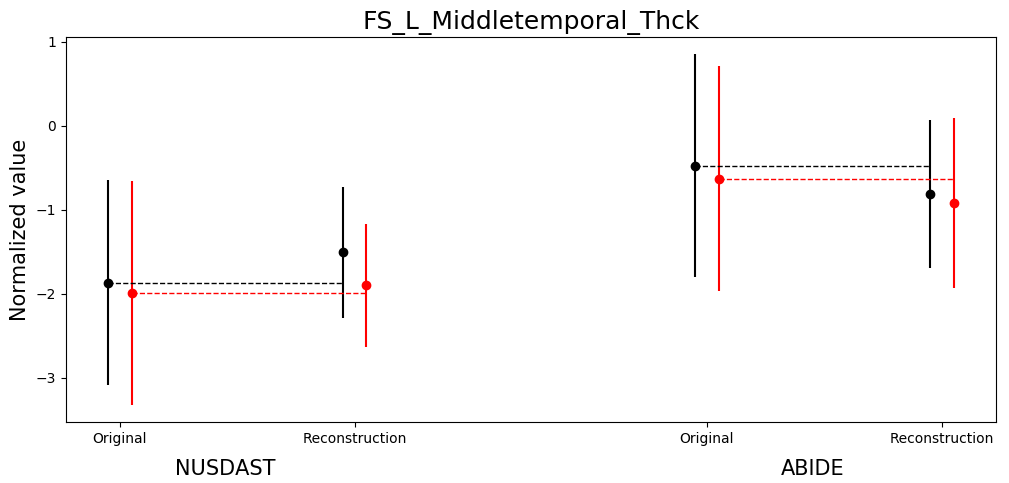

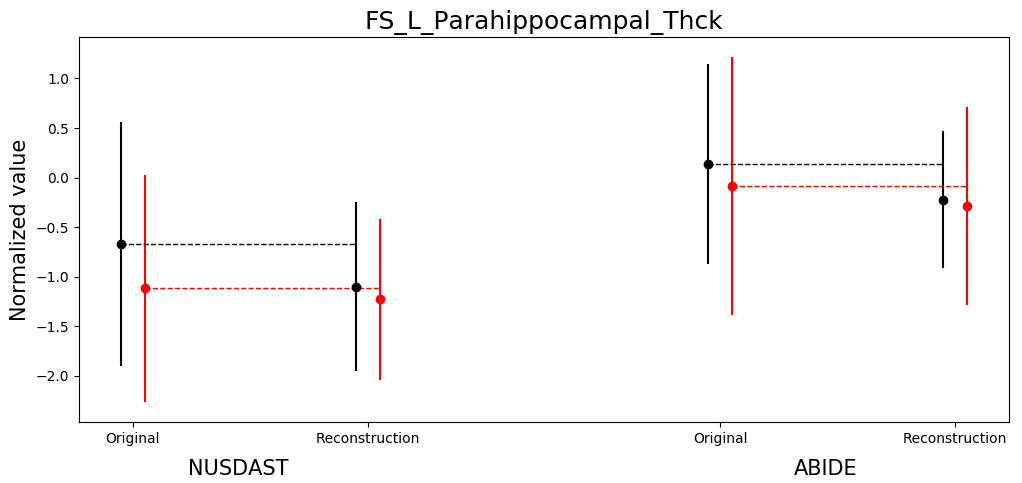

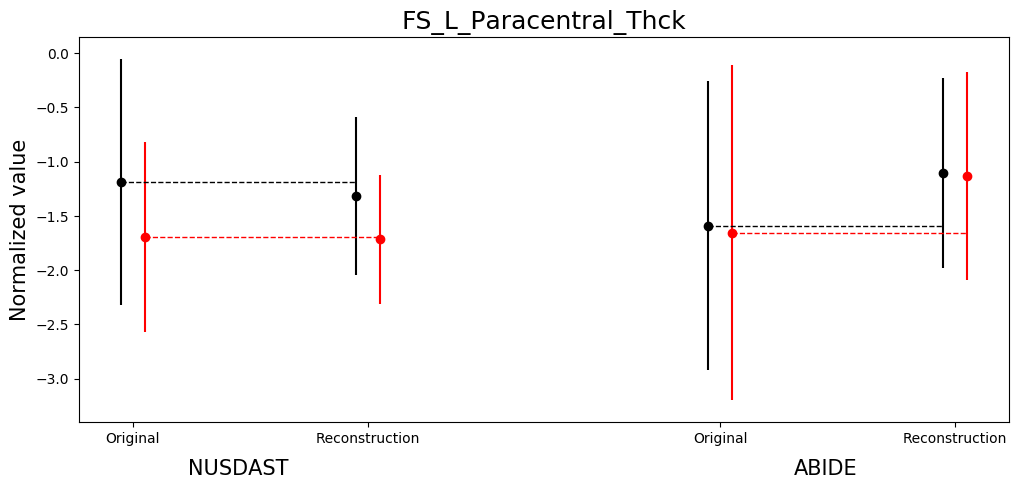

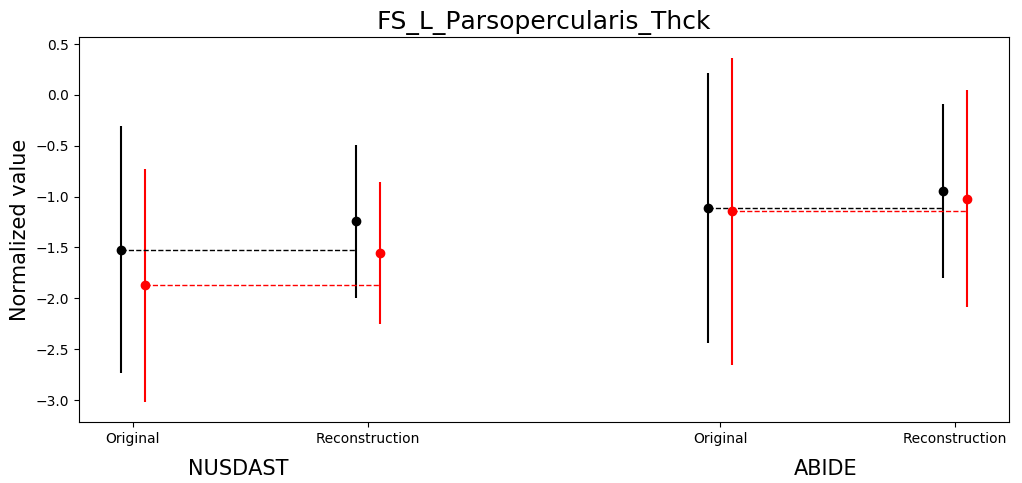

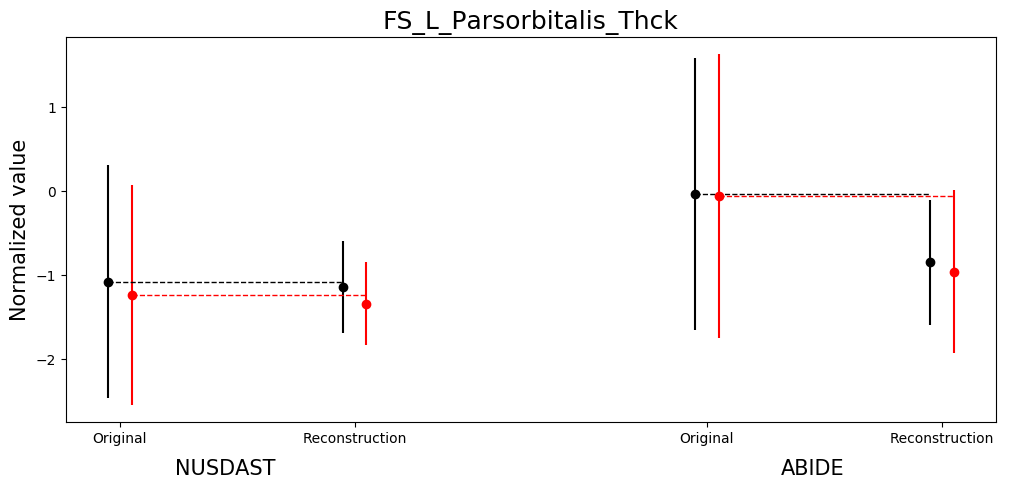

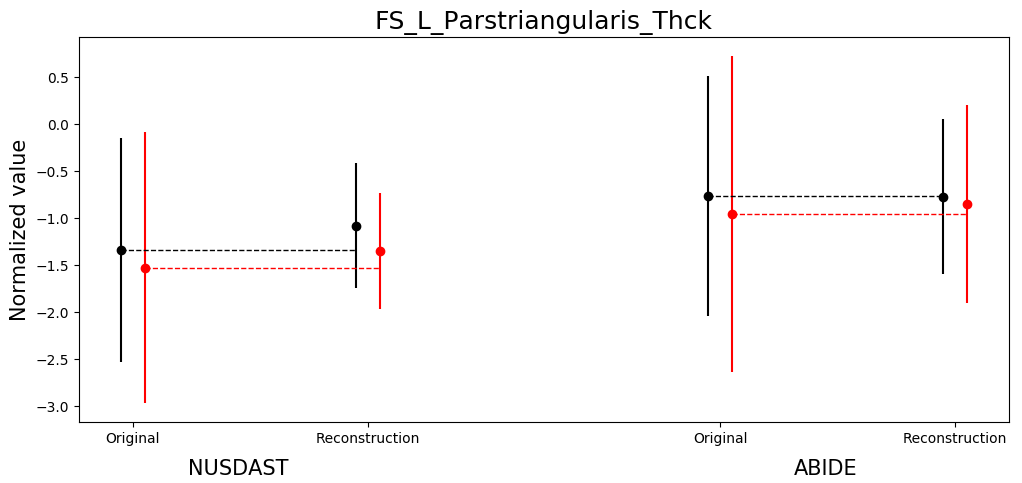

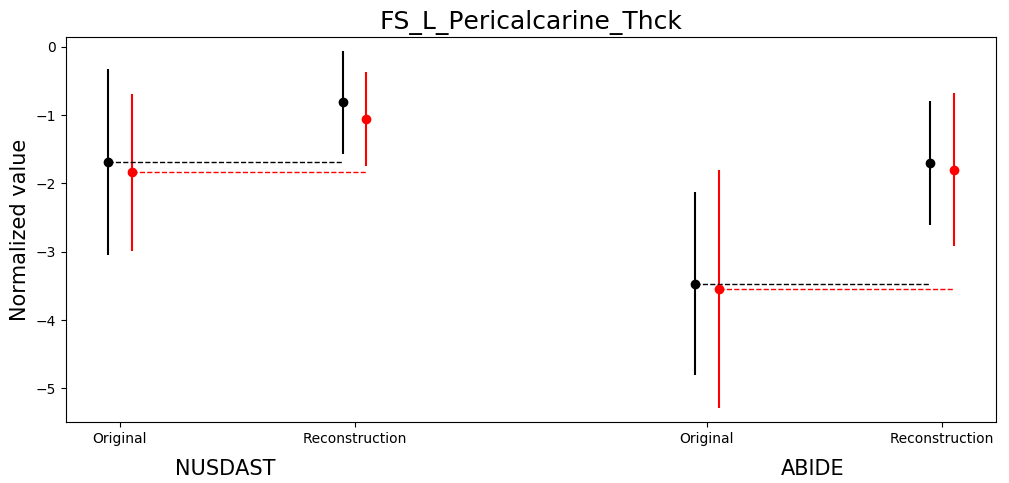

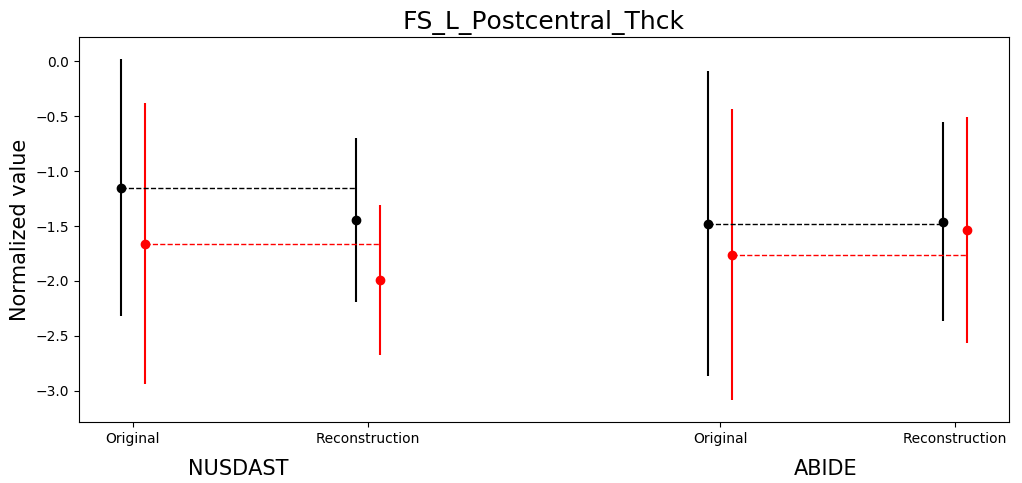

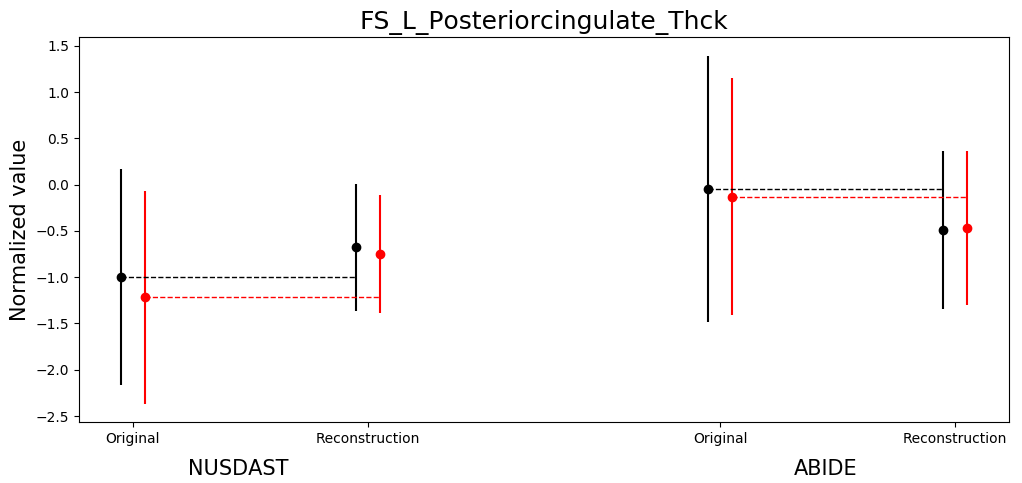

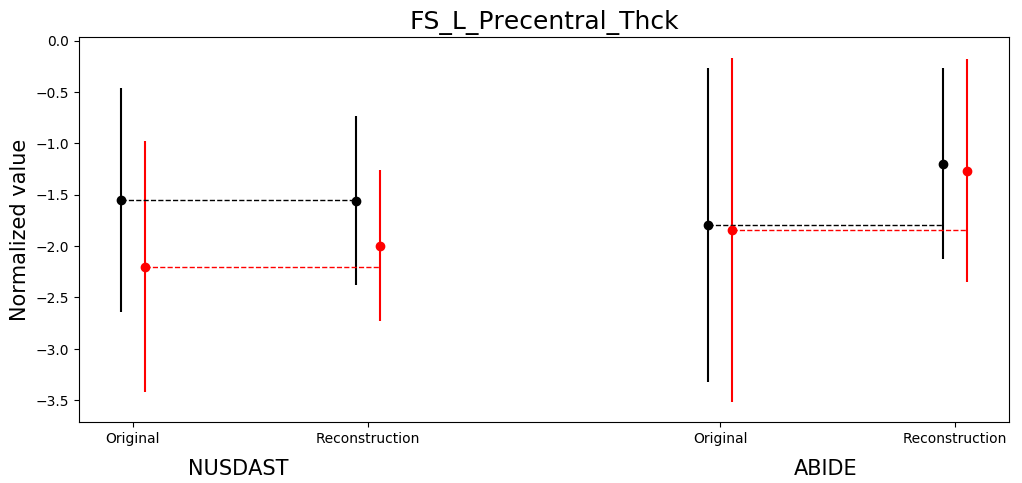

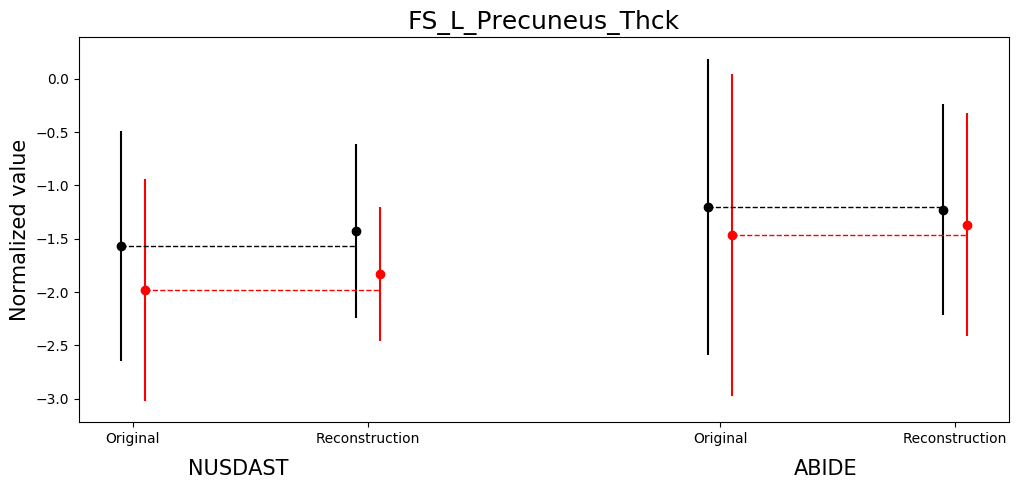

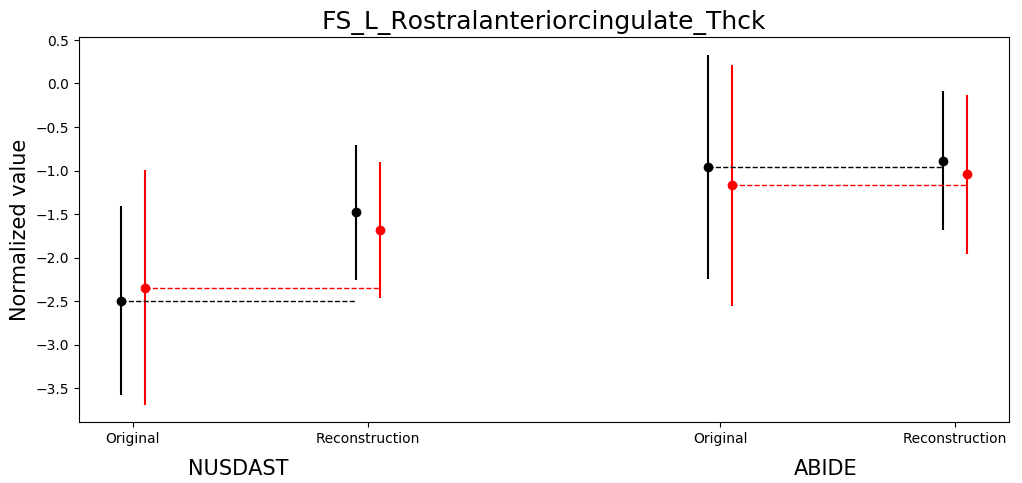

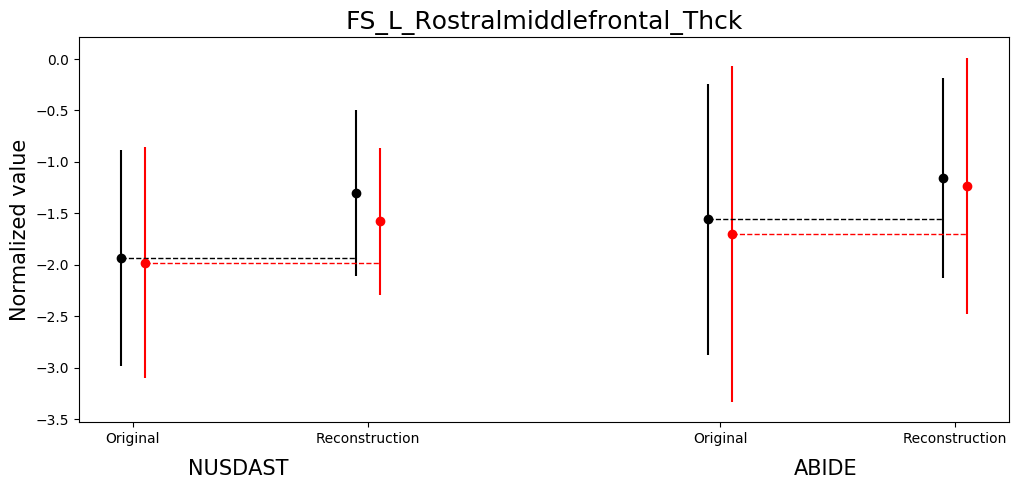

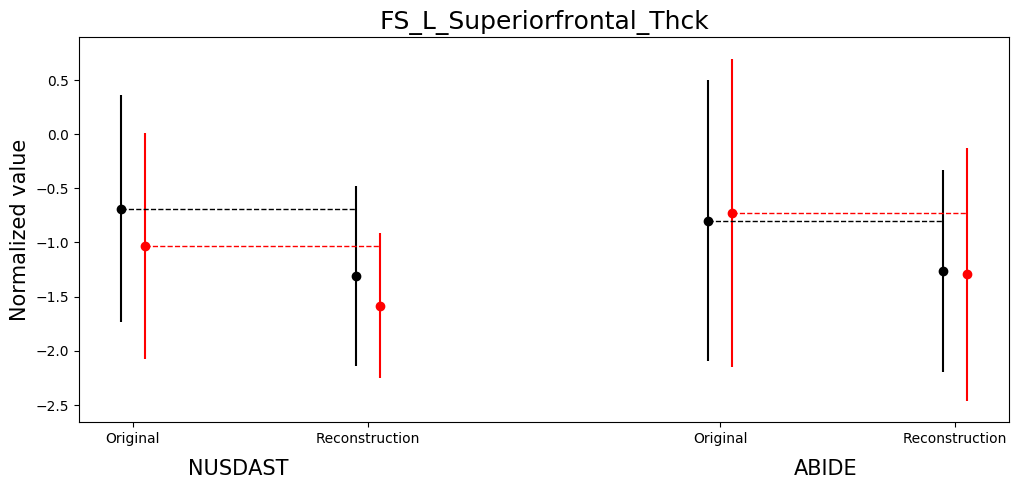

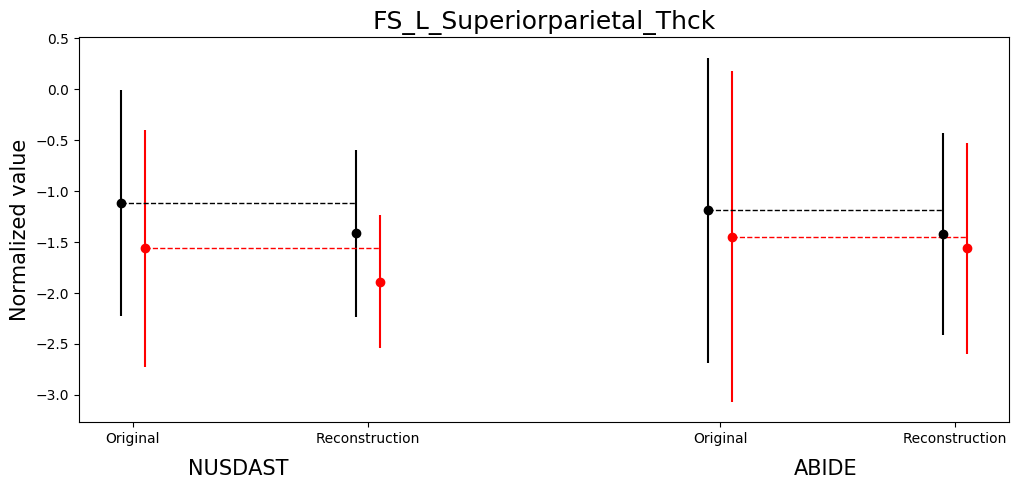

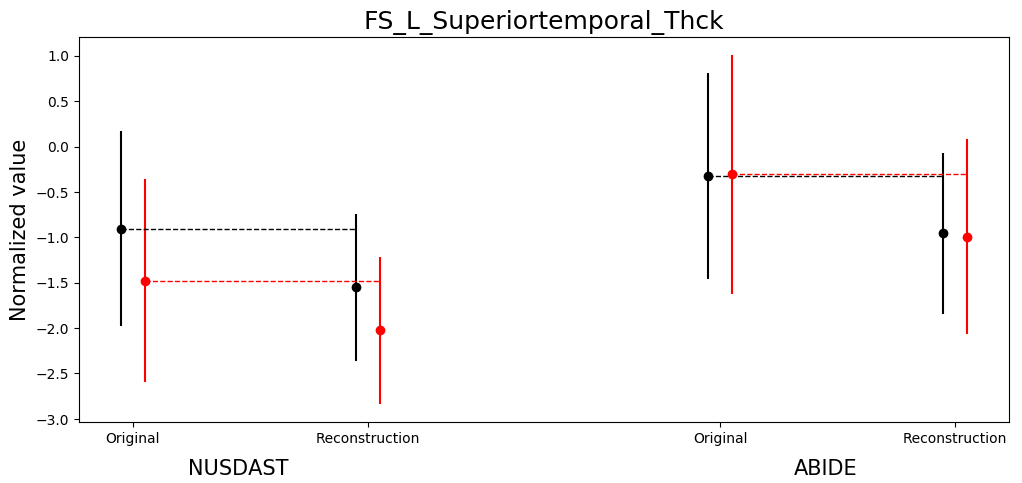

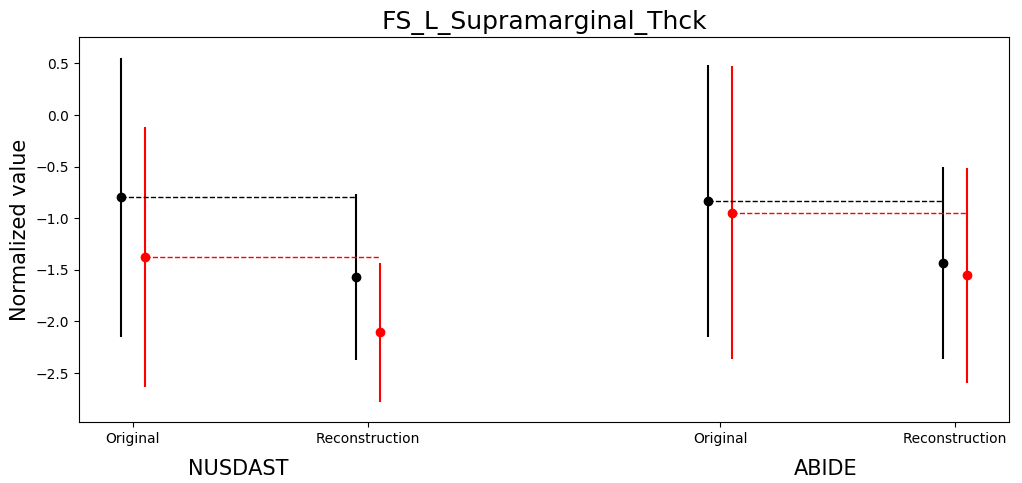

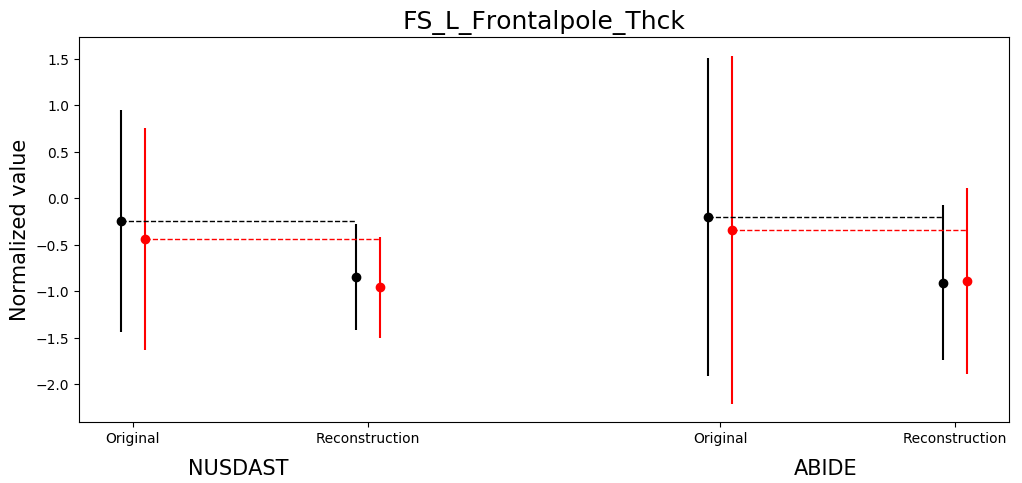

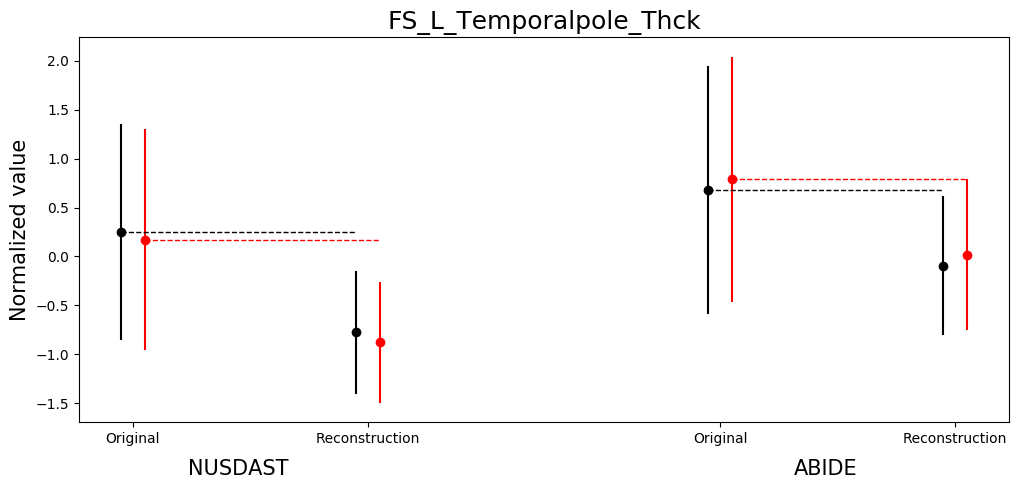

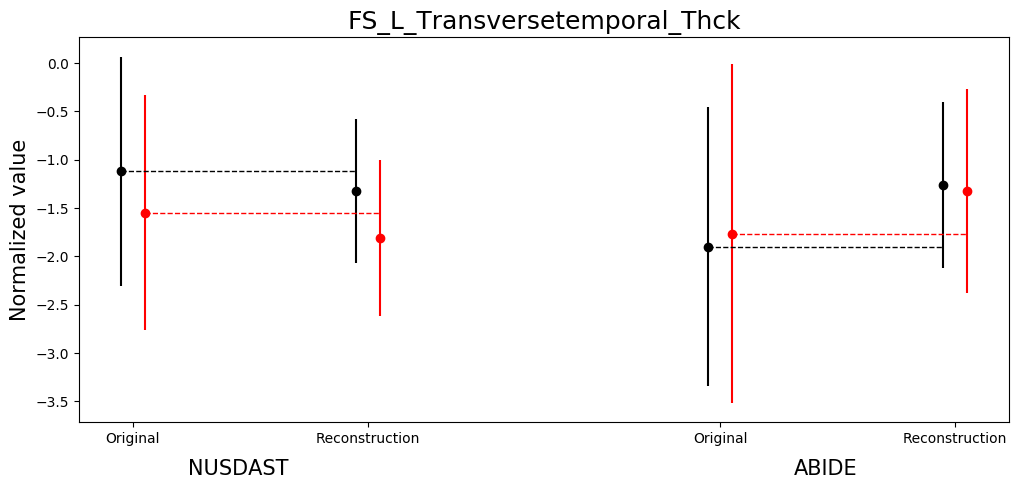

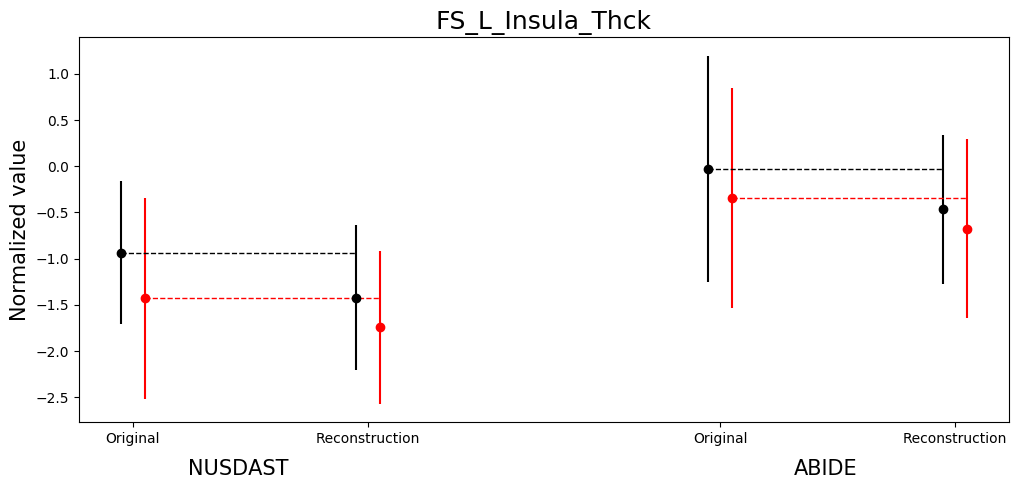

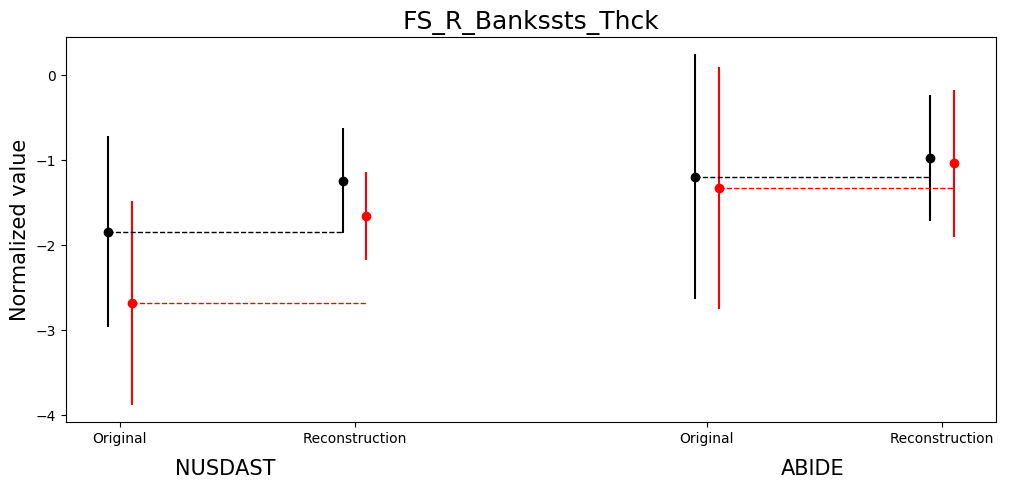

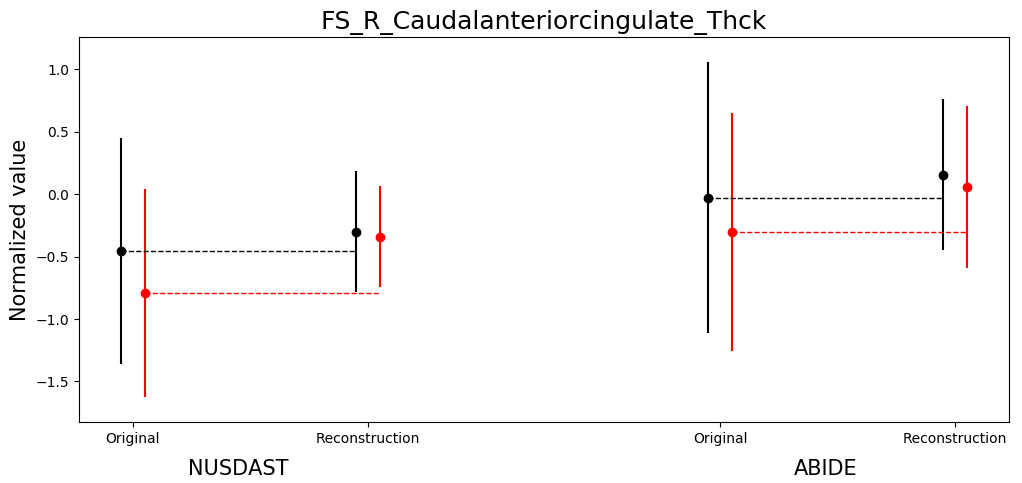

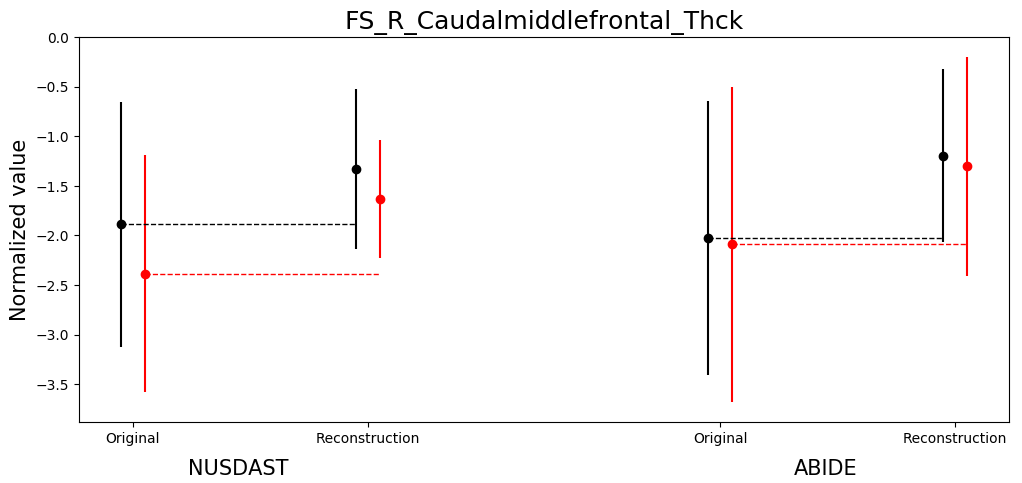

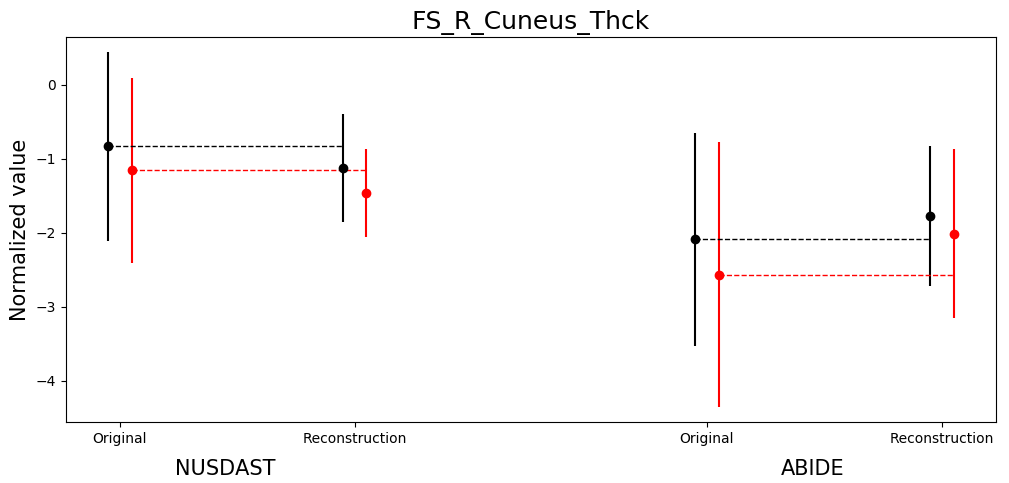

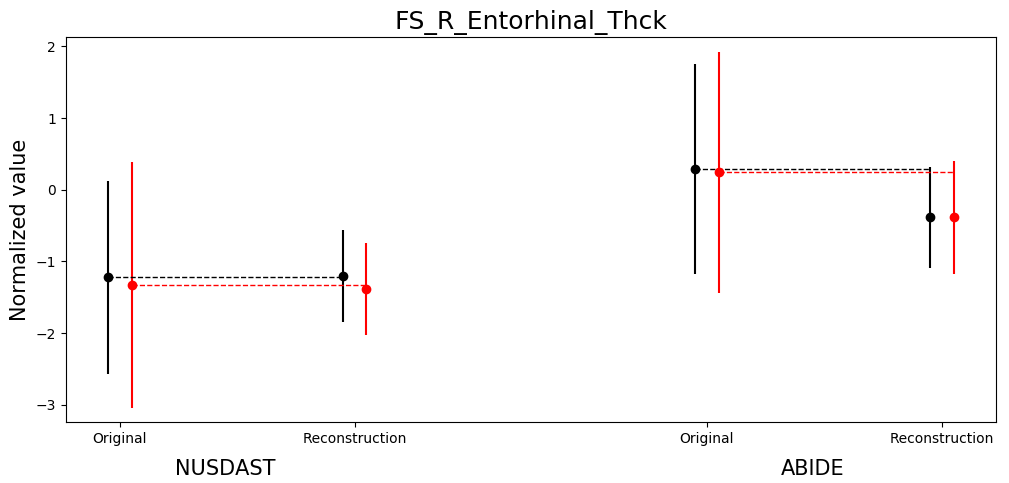

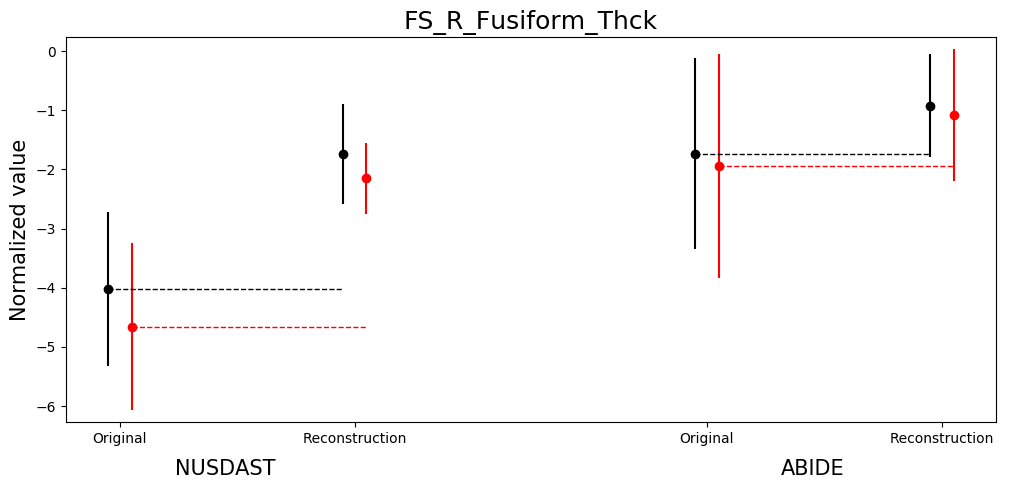

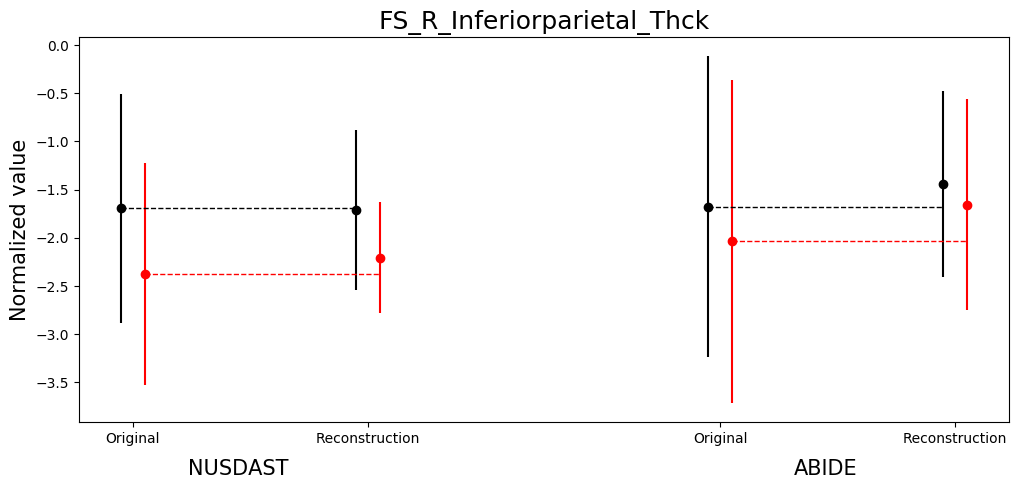

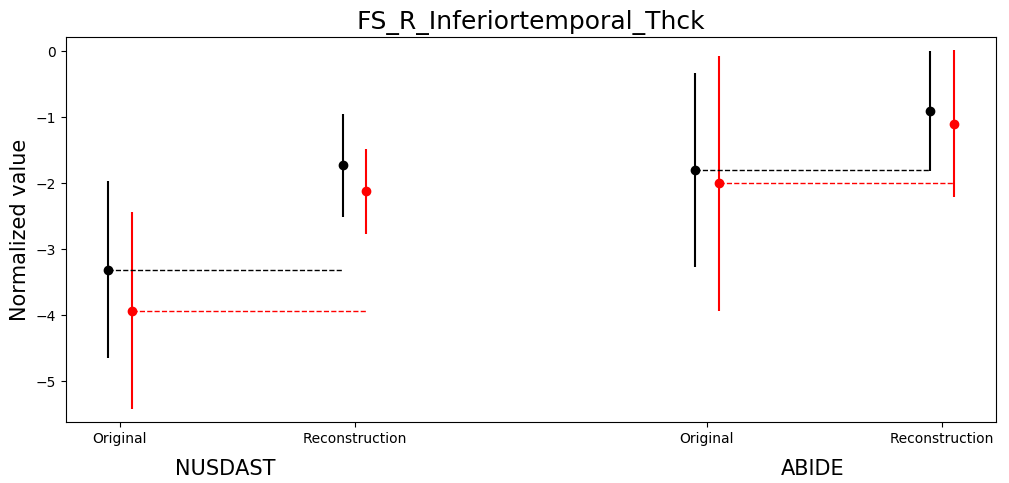

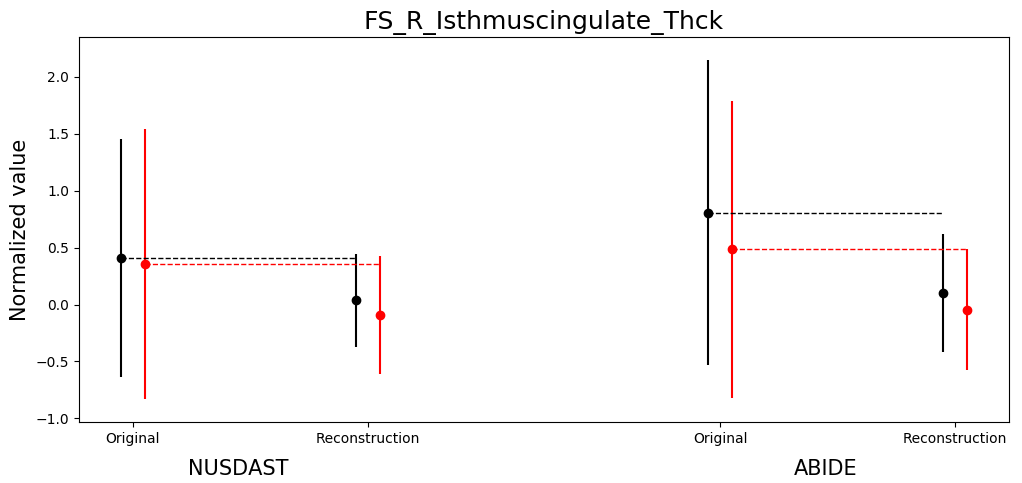

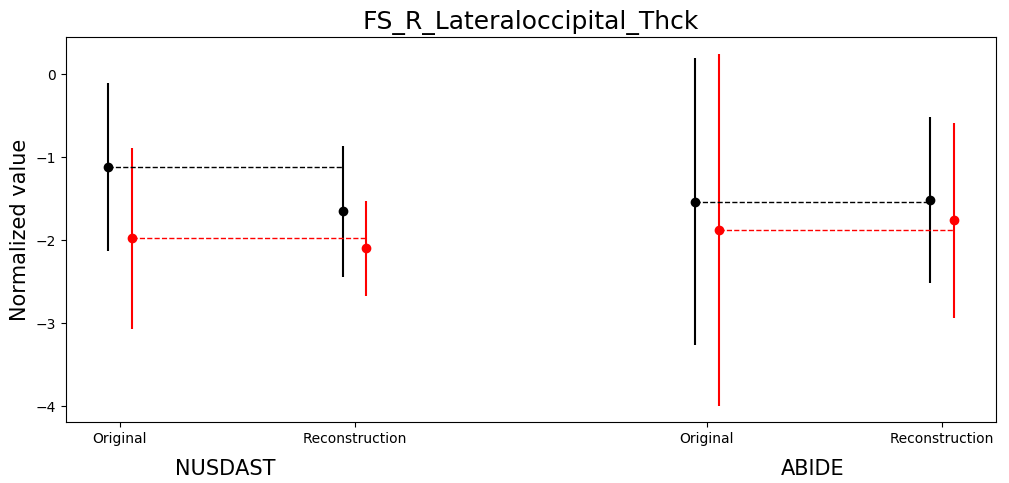

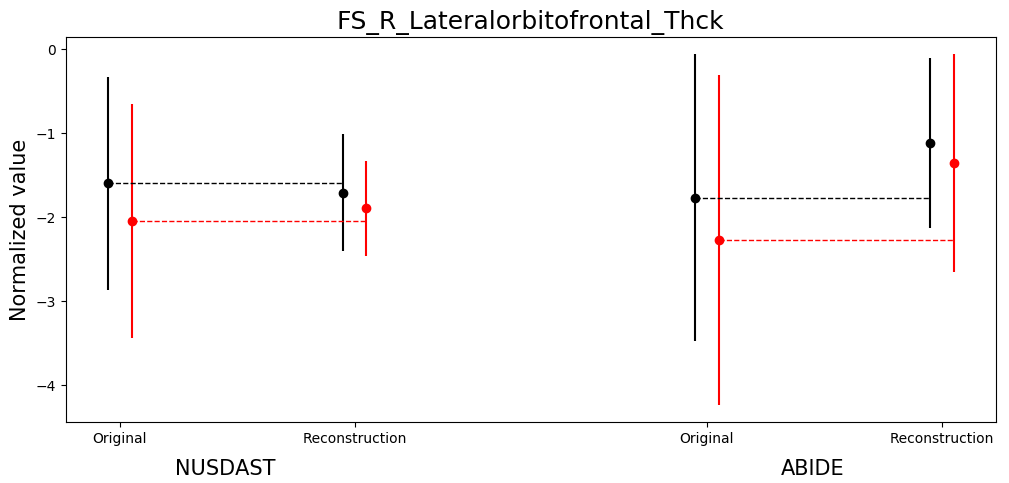

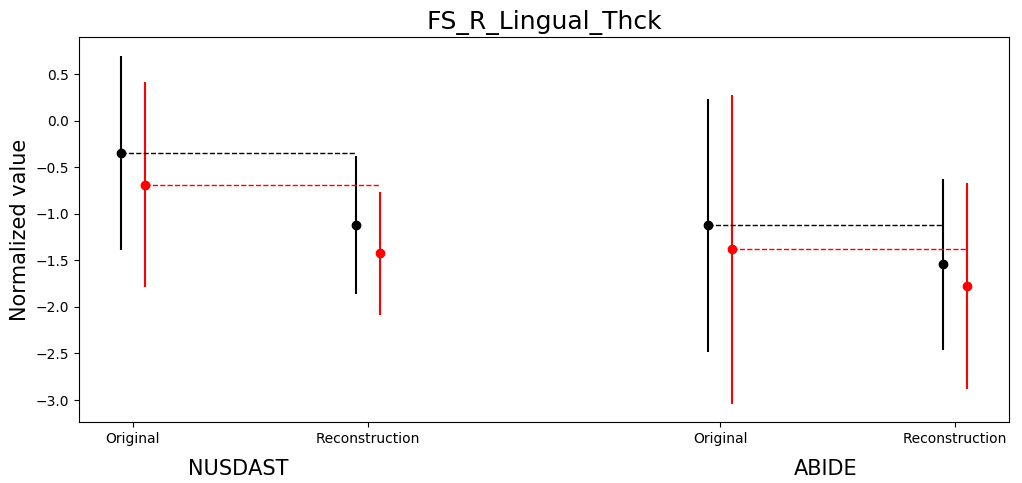

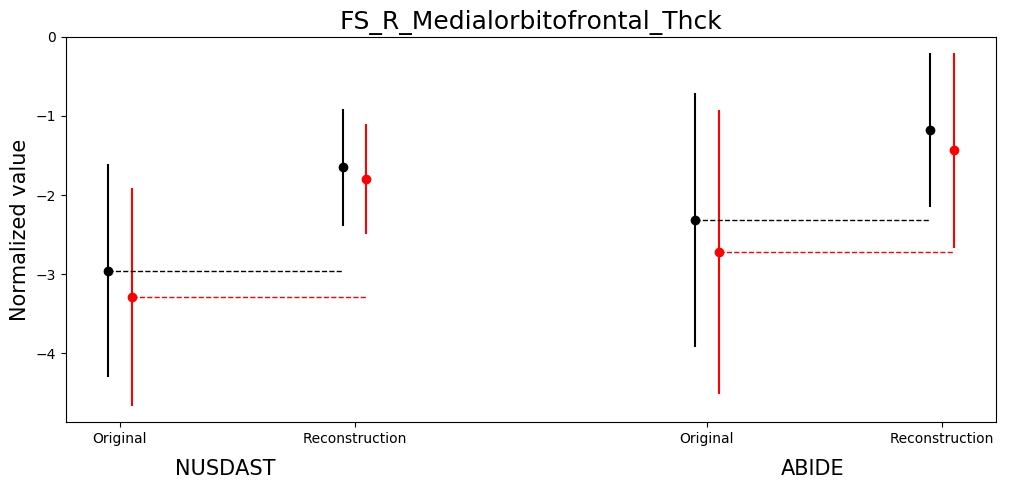

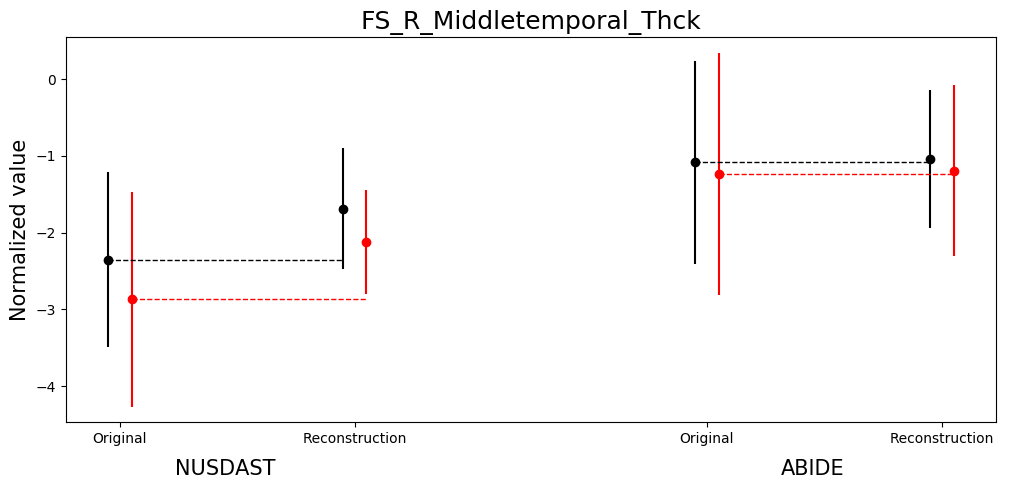

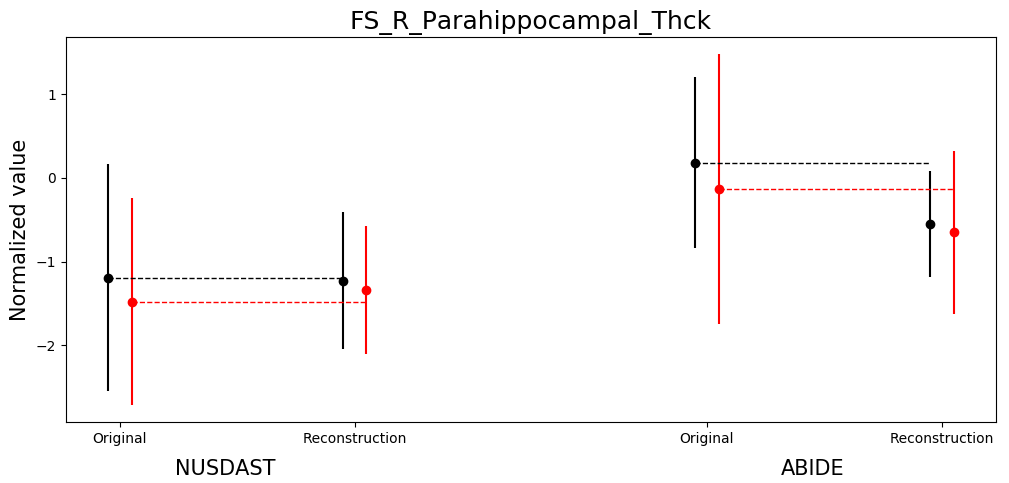

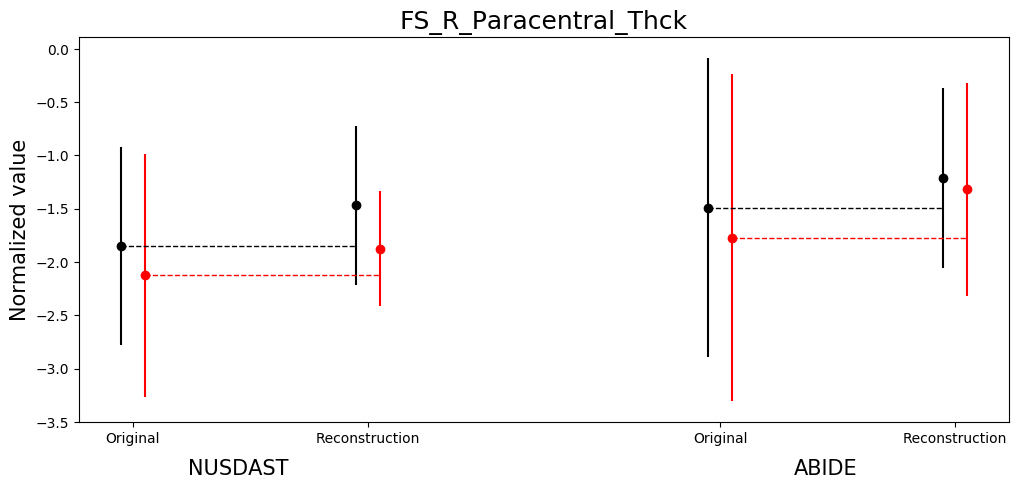

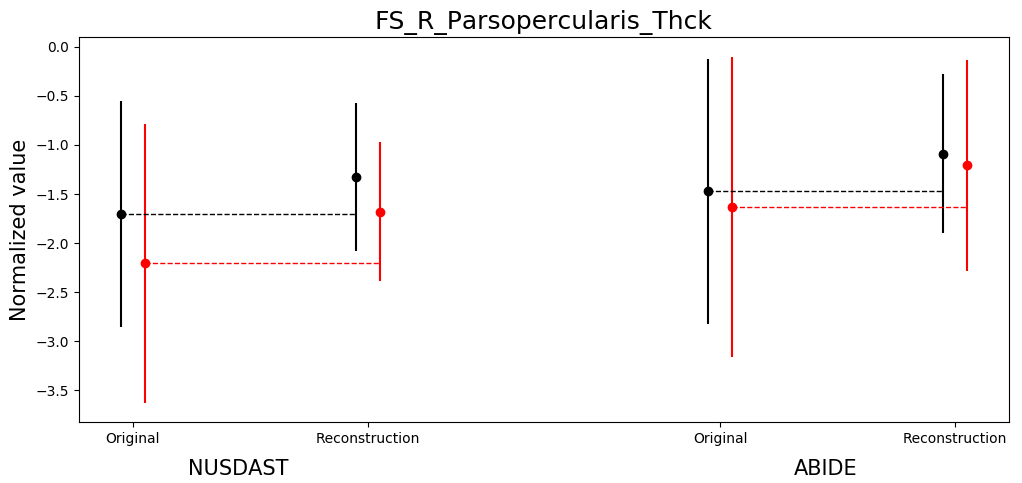

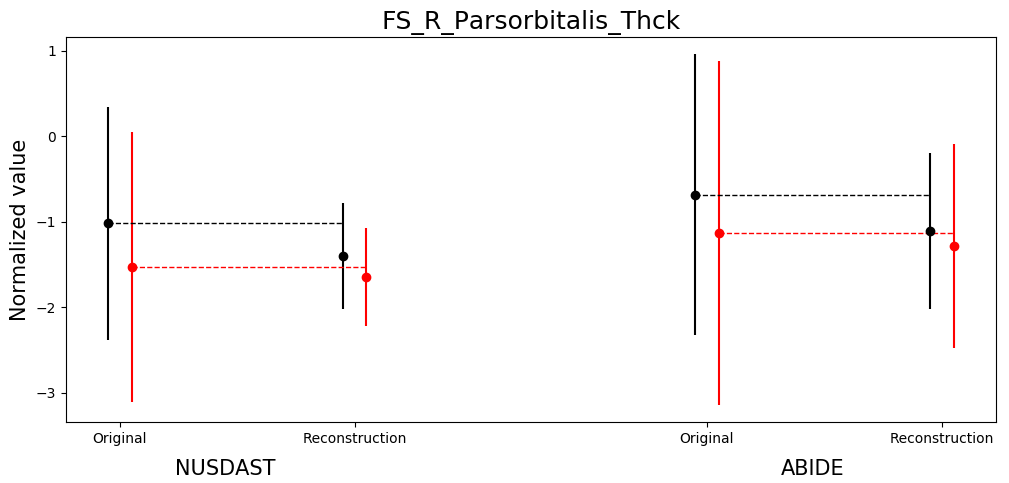


# Mass-univariate analysis of the NUSDAST dataset

Table 4 – Mass univariate analysis of the NUSDAST dataset performed using Mann-Whitney U test and effect size measured by Cliff’s delta value based on the original thickness and volume of each brain region. The significant regions (alpha >= 0.01) are highlighted in bold. Negative values indicate higher values on the patient group.

| **Regions** | **p-value** | **Effect size** | **Regions** | **p-value** | **Effect size** |
| --- | --- | --- | --- | --- | --- |
| Left-Lateral-Ventricle | .3491 | 0.0529 | lh_parsopercularis | .0879 | 0.1829 |
| Left-Inf-Lat-Vent | .0829 | -0.1871 | lh_parsorbitalis | .3336 | 0.0586 |
| Left-Cerebellum-White-Matter | .0210 | 0.2743 | lh_parstriangularis | .3933 | 0.0371 |
| Left-Cerebellum-Cortex | .1041 | 0.1700 | lh_pericalcarine | .3014 | 0.0707 |
| Left-Thalamus-Proper | .0171 | 0.2857 | lh_postcentral | .0472 | 0.2257 |
| Left-Caudate | .2335 | -0.0986 | lh_posteriorcingulate | .4056 | 0.0329 |
| Left-Putamen | .0483 | -0.2243 | **lh_precentral** | **.0091** | **0.3186** |
| **Left-Pallidum** | **.0002** | **-0.4786** | lh_precuneus | .0605 | 0.2093 |
| **3rd-Ventricle** | **.0082** | **-0.3236** | lh_rostralanteriorcingulate | .3491 | -0.0529 |
| 4th-Ventricle | .2534 | 0.0900 | lh_rostralmiddlefrontal | .4451 | 0.0193 |
| Brain-Stem | .1041 | 0.1700 | lh_superiorfrontal | .0829 | 0.1871 |
| Left-Hippocampus | .1683 | 0.1300 | lh_superiorparietal | .0556 | 0.2150 |
| Left-Amygdala | .2534 | 0.0900 | lh_superiortemporal | .0273 | 0.2593 |
| CSF | .2672 | -0.0843 | lh_supramarginal | .0372 | 0.2407 |
| Left-Accumbens-area | .1290 | 0.1529 | lh_frontalpole | .2832 | 0.0779 |
| Left-VentralDC | .1358 | 0.1486 | lh_temporalpole | .4788 | 0.0079 |
| Left-choroid-plexus | .4180 | 0.0286 | lh_transversetemporal | .0521 | 0.2193 |
| Right-Lateral-Ventricle | .3650 | 0.0471 | **lh_insula** | **.0097** | **0.3150** |
| Right-Inf-Lat-Vent | .1552 | -0.1371 | **rh_bankssts** | **.0012** | **0.4100** |
| Right-Cerebellum-White-Matter | .0127 | 0.3014 | rh_caudalanteriorcingulate | .0813 | 0.1886 |
| Right-Cerebellum-Cortex | .0813 | 0.1886 | rh_caudalmiddlefrontal | .0339 | 0.2464 |
| Right-Thalamus-Proper | .0781 | 0.1914 | rh_cuneus | .1833 | 0.1221 |
| Right-Caudate | .1502 | -0.1400 | rh_entorhinal | .2083 | 0.1100 |
| Right-Putamen | .0114 | -0.3071 | rh_fusiform | .0205 | 0.2757 |
| **Right-Pallidum** | **.0018** | **-0.3914** | rh_inferiorparietal | .0103 | 0.3121 |
| Right-Hippocampus | .2238 | 0.1029 | rh_inferiortemporal | .0390 | 0.2379 |
| Right-Amygdala | .2367 | 0.0971 | rh_isthmuscingulate | .3892 | -0.0386 |
| Right-Accumbens-area | .4097 | 0.0314 | **rh_lateraloccipital** | **.0009** | **0.4221** |
| Right-VentralDC | .3374 | 0.0571 | rh_lateralorbitofrontal | .1013 | 0.1721 |
| Right-choroid-plexus | .2832 | -0.0779 | rh_lingual | .0658 | 0.2036 |
| Optic-Chiasm | .2977 | -0.0721 | rh_medialorbitofrontal | .0751 | 0.1943 |
| CC_Posterior | .0665 | 0.2029 | rh_middletemporal | .0638 | 0.2057 |
| CC_Mid_Posterior | .0611 | 0.2086 | rh_parahippocampal | .1478 | 0.1414 |
| CC_Central | .3221 | 0.0629 | rh_paracentral | .0957 | 0.1764 |
| CC_Mid_Anterior | .2052 | 0.1114 | rh_parsopercularis | .0948 | 0.1771 |
| CC_Anterior | .4979 | 0.0000 | rh_parsorbitalis | .1301 | 0.1521 |
| lh_bankssts | .0403 | 0.2357 | rh_parstriangularis | .2191 | 0.1050 |
| lh_caudalanteriorcingulate | .4514 | 0.0171 | rh_pericalcarine | .3912 | 0.0379 |
| lh_caudalmiddlefrontal | .1527 | 0.1386 | **rh_postcentral** | **.0032** | **0.3671** |
| lh_cuneus | .0499 | 0.2221 | rh_posteriorcingulate | .2450 | 0.0936 |
| lh_entorhinal | .1100 | 0.1657 | **rh_precentral** | **.0093** | **0.3171** |
| lh_fusiform | .0477 | 0.2250 | rh_precuneus | .0351 | 0.2443 |
| lh_inferiorparietal | .0129 | 0.3007 | rh_rostralanteriorcingulate | .1130 | 0.1636 |
| **lh_inferiortemporal** | **.0093** | **0.3171** | rh_rostralmiddlefrontal | .2254 | 0.1021 |
| lh_isthmuscingulate | .4242 | 0.0264 | rh_superiorfrontal | .0462 | 0.2271 |
| **lh_lateraloccipital** | **.0027** | **0.3750** | rh_superiorparietal | .0112 | 0.3079 |
| lh_lateralorbitofrontal | .0359 | 0.2429 | rh_superiortemporal | .0287 | 0.2564 |
| lh_lingual | .0343 | 0.2457 | rh_supramarginal | .0109 | 0.3093 |
| lh_medialorbitofrontal | .4619 | 0.0136 | rh_frontalpole | .2400 | 0.0957 |
| lh_middletemporal | .4118 | 0.0307 | rh_temporalpole | .4263 | 0.0257 |
| lh_parahippocampal | .0446 | 0.2293 | rh_transversetemporal | .0158 | 0.2900 |
| lh_paracentral | .0624 | 0.2071 | rh_insula | .2160 | 0.1064 |

# Mass-univariate analysis of the ABIDE dataset

Table 5 - Mass univariate analysis of the ABIDE dataset performed using Mann-Whitney U test and effect size measured by Cliff’s delta value based on the original thickness and volume of each brain region. The significant regions (alpha >= 0.01) are highlighted in bold. Negative values indicate higher values on the patient group.

| **Regions** | **p-value** | **Effect size** | **Regions** | **p-value** | **Effect size** |
| --- | --- | --- | --- | --- | --- |
| Left-Lateral-Ventricle | .0630 | -0.1302 | lh_parsopercularis | .4367 | 0.0137 |
| **Left-Inf-Lat-Vent** | **.0002** | **-0.3020** | lh_parsorbitalis | .3059 | 0.0433 |
| Left-Cerebellum-White-Matter | .4688 | -0.0068 | lh_parstriangularis | .0862 | 0.1161 |
| Left-Cerebellum-Cortex | .1279 | 0.0967 | lh_pericalcarine | .4314 | 0.0148 |
| Left-Thalamus-Proper | .3116 | -0.0419 | lh_postcentral | .0608 | 0.1317 |
| Left-Caudate | .4860 | -0.0031 | lh_posteriorcingulate | .3398 | 0.0352 |
| Left-Putamen | .1776 | -0.0787 | lh_precentral | .4871 | 0.0029 |
| Left-Pallidum | .3212 | -0.0396 | lh_precuneus | .0370 | 0.1520 |
| 3rd-Ventricle | .0912 | -0.1135 | lh_rostralanteriorcingulate | .1557 | 0.0862 |
| 4th-Ventricle | .2809 | 0.0495 | lh_rostralmiddlefrontal | .2067 | 0.0697 |
| Brain-Stem | .2577 | 0.0554 | lh_superiorfrontal | .4463 | 0.0116 |
| Left-Hippocampus | .1481 | -0.0889 | lh_superiorparietal | .0610 | 0.1316 |
| Left-Amygdala | .0969 | -0.1106 | lh_superiortemporal | .4108 | -0.0193 |
| CSF | .3232 | -0.0391 | lh_supramarginal | .2660 | 0.0532 |
| Left-Accumbens-area | .2683 | -0.0527 | lh_frontalpole | .3126 | 0.0417 |
| Left-VentralDC | .0600 | -0.1323 | lh_temporalpole | .3543 | -0.0319 |
| Left-choroid-plexus | .2025 | 0.0709 | lh_transversetemporal | .1522 | -0.0874 |
| Right-Lateral-Ventricle | .4892 | 0.0024 | lh_insula | .0608 | 0.1317 |
| Right-Inf-Lat-Vent | .0157 | -0.1830 | rh_bankssts | .3638 | 0.0297 |
| Right-Cerebellum-White-Matter | .3822 | -0.0256 | rh_caudalanteriorcingulate | .0681 | 0.1268 |
| Right-Cerebellum-Cortex | .3339 | 0.0366 | rh_caudalmiddlefrontal | .2298 | 0.0630 |
| Right-Thalamus-Proper | .3092 | 0.0425 | rh_cuneus | .0215 | 0.1721 |
| Right-Caudate | .4346 | 0.0141 | rh_entorhinal | .4887 | -0.0025 |
| Right-Putamen | .4293 | -0.0153 | rh_fusiform | .4272 | 0.0157 |
| Right-Pallidum | .3623 | -0.0301 | rh_inferiorparietal | .0449 | 0.1443 |
| Right-Hippocampus | .0322 | -0.1573 | rh_inferiortemporal | .3300 | 0.0375 |
| Right-Amygdala | .1123 | -0.1034 | rh_isthmuscingulate | .0774 | 0.1211 |
| Right-Accumbens-area | .4193 | -0.0174 | rh_lateraloccipital | .2499 | 0.0575 |
| Right-VentralDC | .1942 | -0.0734 | rh_lateralorbitofrontal | .0414 | 0.1476 |
| Right-choroid-plexus | .2951 | 0.0459 | rh_lingual | .1920 | 0.0741 |
| Optic-Chiasm | .2322 | -0.0623 | rh_medialorbitofrontal | .0812 | 0.1189 |
| CC_Posterior | .0332 | 0.1562 | rh_middletemporal | .1762 | 0.0792 |
| CC_Mid_Posterior | .3373 | 0.0358 | rh_parahippocampal | .0903 | 0.1139 |
| CC_Central | .4219 | 0.0169 | rh_paracentral | .0757 | 0.1221 |
| CC_Mid_Anterior | .4618 | 0.0083 | rh_parsopercularis | .1738 | 0.0800 |
| CC_Anterior | .0213 | 0.1725 | rh_parsorbitalis | .0135 | 0.1881 |
| lh_bankssts | .1593 | 0.0849 | rh_parstriangularis | .1115 | 0.1037 |
| lh_caudalanteriorcingulate | .0966 | 0.1107 | rh_pericalcarine | .3433 | 0.0344 |
| lh_caudalmiddlefrontal | .4469 | -0.0115 | rh_postcentral | .1928 | 0.0739 |
| lh_cuneus | .1077 | 0.1055 | rh_posteriorcingulate | .2205 | 0.0656 |
| lh_entorhinal | .3931 | -0.0232 | rh_precentral | .2410 | 0.0599 |
| lh_fusiform | .1797 | 0.0780 | **rh_precuneus** | **.0036** | **0.2289** |
| lh_inferiorparietal | .0205 | 0.1738 | rh_rostralanteriorcingulate | .1042 | 0.1071 |
| lh_inferiortemporal | .4293 | -0.0153 | rh_rostralmiddlefrontal | .1194 | 0.1003 |
| lh_isthmuscingulate | .1128 | 0.1032 | rh_superiorfrontal | .2905 | 0.0470 |
| lh_lateraloccipital | .0289 | 0.1614 | rh_superiorparietal | .0786 | 0.1204 |
| lh_lateralorbitofrontal | .0888 | 0.1147 | rh_superiortemporal | .2478 | 0.0581 |
| lh_lingual | .4667 | 0.0072 | rh_supramarginal | .1348 | 0.0940 |
| lh_medialorbitofrontal | .4298 | 0.0151 | rh_frontalpole | .2397 | 0.0602 |
| lh_middletemporal | .2129 | 0.0678 | rh_temporalpole | .4415 | -0.0126 |
| lh_parahippocampal | .1351 | 0.0939 | rh_transversetemporal | .0990 | 0.1096 |
| lh_paracentral | .3275 | 0.0381 | rh_insula | .0463 | 0.1431 |

# Performance of the SVM classifiers

Table 6 - Performance of the linear SVM classifiers in the NUSDAST and ABIDE datasets. These performances were obtain using a bootstrap resampling method using 10,000 repetitions. These metrics were obtained in the task of classification between healthy controls and patients.

| Dataset | Balance accuracy | Sensitivity | Specificity | Error rate |
| --- | --- | --- | --- | --- |
| NUSDAST | .588 [.462, .766] | .529 [.222, .824] | .650 [.400, .875] | .415 [.282, .543] |
| ABIDE | .552 [.460, .63] | .476 [.308, .659] | .621 [.453, .778] | .443 [.352, .535] |

*Values presented as median estimate [95% confidence interval].

# References

Dray S (2008): On the number of principal components: A test of dimensionality based on measurements of similarity between matrices. Comput Stat Data Anal 52:2228–2237.

Van Essen DC, Smith SM, Barch DM, Behrens TEJ, Yacoub E, Ugurbil K (2013): The WU-Minn Human Connectome Project: An overview. Neuroimage 80:62–79. http://dx.doi.org/10.1016/j.neuroimage.2013.05.041.

Grün F, Rupprecht C, Navab N, Tombari F (2016): A Taxonomy and Library for Visualizing Learned Features in Convolutional Neural Networks. arXiv Prepr arXiv160607757.

Di Martino A, Yan C-G, Li Q, Denio E, Castellanos FX, Alaerts K, Anderson JS, Assaf M, Bookheimer SY, Dapretto M (2014): The autism brain imaging data exchange: towards a large-scale evaluation of the intrinsic brain architecture in autism. Mol Psychiatry 19:659–667.

Milham MP, Fair D, Mennes M, Mostofsky SHMD (2012): The ADHD-200 consortium: a model to advance the translational potential of neuroimaging in clinical neuroscience. Front Syst Neurosci 6:62.

Selvaraju RR, Das A, Vedantam R, Cogswell M, Parikh D, Batra D (2016): Grad-cam: Why did you say that? visual explanations from deep networks via gradient-based localization. arXiv Prepr arXiv161002391.

Smilkov D, Thorat N, Kim B, Viégas F, Wattenberg M (2017): SmoothGrad: removing noise by adding noise. arXiv Prepr arXiv170603825.

Springenberg JT, Dosovitskiy A, Brox T, Riedmiller M (2014): Striving for simplicity: The all convolutional net. arXiv Prepr arXiv14126806.

Valle S, Li W, Qin SJ (1999): Selection of the number of principal components: the variance of the reconstruction error criterion with a comparison to other methods. Ind Eng Chem Res 38:4389–4401.

Wang L, Alpert KI, Calhoun VD, Cobia DJ, Keator DB, King MD, Kogan A, Landis D, Tallis M, Turner MD, Potkin SG, Turner JA, Ambite JL (2016): SchizConnect: Mediating neuroimaging databases on schizophrenia and related disorders for large-scale integration. Neuroimage 124:1155–1167. http://dx.doi.org/10.1016/j.neuroimage.2015.06.065.
